# Effect of Androgen Suppression on Clinical Outcomes in Hospitalized Men With COVID-19: The HITCH Randomized Clinical Trial

**DOI:** 10.1001/jamanetworkopen.2022.7852

**Published:** 2022-04-19

**Authors:** Nicholas G. Nickols, Zhibao Mi, Ellen DeMatt, Kousick Biswas, Christina E. Clise, John T. Huggins, Spyridoula Maraka, Elena Ambrogini, Mehdi S. Mirsaeidi, Ellis R. Levin, Daniel J. Becker, Danil V. Makarov, Victor Adorno Febles, Pooja M. Belligund, Mohammad Al-Ajam, Muthiah P. Muthiah, Robert B. Montgomery, Kyle W. Robinson, Yu-Ning Wong, Roger J. Bedimo, Reina C. Villareal, Samuel M. Aguayo, Martin W. Schoen, Matthew B. Goetz, Christopher J. Graber, Debika Bhattacharya, Guy Soo Hoo, Greg Orshansky, Leslie E. Norman, Samantha Tran, Leila Ghayouri, Sonny Tsai, Michelle Geelhoed, Mathew B. Rettig

**Affiliations:** 1 Radiation Oncology Service, VA Greater Los Angeles Healthcare System, Los Angeles, California; 2 Department of Radiation Oncology, University of California, Los Angeles; 3 Department of Urology, University of California, Los Angeles; 4 VA Cooperative Studies Program Coordinating Center, Perry Point, Maryland; 5 VA Cooperative Studies Program Clinical Research Pharmacy Coordinating Center, Albuquerque, New Mexico; 6 Pulmonary and Critical Care Medicine, Ralph H. Johnson VA Medical Center, Charleston, South Carolina; 7 Division of Pulmonary, Critical Care, Allergy and Sleep Medicine, Medical University of South Carolina, Charleston; 8 Medicine Service, Central Arkansas Veterans Healthcare System, Little Rock; 9 Division of Endocrinology and Metabolism, University of Arkansas for Medical Sciences, Little Rock; 10 Division of Pulmonary, Critical Care and Sleep, College of Medicine–Jacksonville, University of Florida, Jacksonville; 11 Division of Endocrinology, Long Beach VA Medical Center, Long Beach, California; 12 Division of Endocrinology, Department of Medicine, University of California, Irvine; 13 Division of Hematology and Oncology VA New York Harbor Healthcare System, Manhattan Campus, New York; 14 Perlmutter Cancer Center, NYU Langone Medical Center, New York, New York; 15 VA New York Harbor Healthcare System, Manhattan Campus, New York; 16 NYU Grossman School of Medicine, New York, New York; 17 VA New York Harbor Healthcare System, Brooklyn Campus, Brooklyn; 18 Veterans Affairs Medical Center, Memphis, Tennessee; 19 University of Tennessee Health Science Center, Memphis; 20 Division of Hematology and Oncology, VA Puget Sound Health Care System, Seattle, Washington; 21 Division of Medical Oncology, Department of Medicine, University of Washington, Seattle; 22 Department of Hematology and Oncology, Corporal Michael J. Crescenz VA Medical Center, Philadelphia, Pennsylvania; 23 Division of Hematology-Oncology, Perelman School of Medicine, University of Pennsylvania, Philadelphia; 24 VA North Texas Health Care System, Dallas; 25 UT Southwestern Medical Center, School of Medicine, Dallas, Texas; 26 Michael E. DeBakey VA Medical Center, Houston, Texas; 27 Pulmonary and Critical Care Medicine, Phoenix VA Health Care System, Phoenix, Arizona; 28 John Cochran Veterans Affairs Medical Center, St Louis, Missouri; 29 Department of Medicine, Saint Louis University School of Medicine, St Louis, Missouri; 30 Infectious Diseases Section, VA Greater Los Angeles Healthcare System, Los Angeles, California; 31 Department of Medicine, University of California, Los Angeles; 32 Pulmonary, Critical Care and Sleep Section, VA Greater Los Angeles Healthcare System, Los Angeles, California; 33 Clinical Informatics, VA Greater Los Angeles Healthcare System, Los Angeles, California; 34 Division of Hematology-Oncology, Department of Medicine, VA Greater Los Angeles Healthcare System, Los Angeles, California; 35 Departments of Medicine and Urology, University of California, Los Angeles

## Abstract

**Importance:**

SARS-CoV-2 entry requires the TMPRSS2 cell surface protease. Antiandrogen therapies reduce expression of TMPRSS2.

**Objective:**

To determine if temporary androgen suppression induced by degarelix improves clinical outcomes of inpatients hospitalized with COVID-19.

**Design, Setting, and Participants:**

The Hormonal Intervention for the Treatment in Veterans With COVID-19 Requiring Hospitalization (HITCH) phase 2, placebo-controlled, double-blind, randomized clinical trial compared efficacy of degarelix plus standard care vs placebo plus standard care on clinical outcomes in men hospitalized with COVID-19 but not requiring invasive mechanical ventilation. Inpatients were enrolled at 14 Department of Veterans Affairs hospitals from July 22, 2020, to April 8, 2021. Data were analyzed from August 9 to October 15, 2021.

**Interventions:**

Patients stratified by age, history of hypertension, and disease severity were centrally randomized 2:1 to degarelix, (1-time subcutaneous dose of 240 mg) or a saline placebo. Standard care included but was not limited to supplemental oxygen, antibiotics, vasopressor support, peritoneal dialysis or hemodialysis, intravenous fluids, remdesivir, convalescent plasma, and dexamethasone.

**Main Outcomes and Measures:**

The composite primary end point was mortality, ongoing need for hospitalization, or requirement for mechanical ventilation at day 15 after randomization. Secondary end points were time to clinical improvement, inpatient mortality, length of hospitalization, duration of mechanical ventilation, time to achieve a temperature within reference range, maximum severity of COVID-19, and the composite end point at 30 days.

**Results:**

The trial was stopped for futility after the planned interim analysis, at which time there were 96 evaluable patients, including 62 patients randomized to the degarelix group and 34 patients in the placebo group, out of 198 initially planned. The median (range) age was 70.5 (48-85) years. Common comorbidities included chronic obstructive pulmonary disorder (15 patients [15.6%]), hypertension (75 patients [78.1%]), cardiovascular disease (27 patients [28.1%]), asthma (12 patients [12.5%]), diabetes (49 patients [51.0%]), and chronic respiratory failure requiring supplemental oxygen at baseline prior to COVID-19 (9 patients [9.4%]). For the primary end point, there was no significant difference between the degarelix and placebo groups (19 patients [30.6%] vs 9 patients [26.5%]; *P* = .67). Similarly, no differences were observed between degarelix and placebo groups in any secondary end points, including inpatient mortality (11 patients [17.7%] vs 6 patients [17.6%]) or all-cause mortality (11 patients [17.7%] vs 7 patents [20.6%]). There were no differences between degarelix and placebo groups in the overall rates of adverse events (13 patients [21.0%] vs 8 patients [23.5%) and serious adverse events (19 patients [30.6%] vs 13 patients [32.4%]), nor unexpected safety concerns.

**Conclusions and Relevance:**

In this randomized clinical trial of androgen suppression vs placebo and usual care for men hospitalized with COVID-19, degarelix did not result in amelioration of COVID-19 severity.

**Trial Registration:**

ClinicalTrials.gov Identifier: NCT04397718

## Key Points

**Question:**

Does androgen suppression improve clinical outcomes in hospitalized men with COVID-19?

**Findings:**

In this randomized clinical trial including 96 men, androgen suppression with the addition of degarelix vs placebo plus standard care did not show reduction of the composite end point of mortality, ongoing hospitalization, or requirement for mechanical ventilation at day 15 after randomization.

**Meaning:**

This randomized clinical trial found that androgen suppression did not improve outcomes in men hospitalized for COVID-19.

## Teaser

This randomized clinical trial assesses the effect of androgen suppression induced by degarelix vs placebo plus standard care for clinical outcomes among men hospitalized with COVID-19.

## Introduction

The predominant mechanism for entry of SARS-CoV-2 into host cells requires recognition of the host cell angiotensin converting enzyme 2 (ACE2) receptor by the viral spike protein and proteolytic activation of the viral spike protein by the host cell transmembrane protease 2 (TMPRSS2).^1^ Pharmacologic targeting of TMPRSS2 or ACE2 might reduce SARS-CoV-2 viral entry and severity of COVID-19. *TMPRSS2* gene expression, initially characterized within the prostate gland, is regulated by the androgen receptor (AR). The AR is activated by circulating androgens, which trigger translocation of the AR to the nucleus and transcription of target genes.^2^ AR, TMPRSS2, and ACE2 are expressed together in human lung epithelial cells.^3,4^ Androgens enrich AR binding at the TMPRSS2 enhancer and upregulate expression of *TMPRSS2*. In mice, androgen suppression reversibly reduces both TMPRSS2 and ACE2 in lung bronchial cells.^3,4^ Moreover, the highly variable expression pattern of *TMPRSS2* suggests a provocative and plausible, although unproven, explanation for the wide range in disease severity for individuals infected by SARS-Cov-2, as well as the higher rate of severe infections among men and the reduced rate and severity of infection in prepubertal children. In cultured human cells, pharmacologic targeting of the AR reduces SARS-CoV-2 infection.^4^ Retrospective analyses have supported a potential protective role for suppression of the AR against COVID-19 incidence and severity,^5,6^ although this has not been consistently observed.^7,8,9^ Clinical trials of AR antagonists in the outpatient setting report accelerated viral clearance and reduced rates of hospitalization.^10,11^ The recently published Phase II Enzalutamide Treatment in COVID-19 (COVIDENZA) trial tested the effect of the AR antagonist enzalutamide in hospitalized patients with COVID-19 and enrolled contemporaneous to the Hormonal Intervention for the Treatment in Veterans With COVID-19 Requiring Hospitalization (HITCH) trial.^12^ No effect from enzalutamide was observed.^12^ Suppression of AR transcriptional activity can be achieved either via direct interference in androgen-AR binding via an AR antagonist or by lowering circulating androgens.

Degarelix is a prostate cancer therapy approved by the US Food and Drug Administration (FDA) and a luteinizing hormone releasing hormone antagonist. Its immediate onset of action, binding to the gonadotropin-releasing hormone receptors in the pituitary gland, rapidly suppresses luteinizing and follicle-stimulating hormone secretion, thereby decreasing testosterone production within the testes and rapidly reducing circulating androgen levels.^13^ One loading dose of 240 mg subcutaneously serves as a 30-day depot; castrate levels of serum testosterone (<50 ng/dL; to convert to nanomoles per liter, multiply by 0.0347) are achieved within 72 hours in more than 90% of patients. The primary objective of this study was to determine if temporary androgen suppression induced by degarelix improves clinical outcomes of men who are hospitalized on an acute care ward owing to COVID-19 as defined by a reduction in mortality, ongoing need for hospitalization, or requirement for mechanical ventilation. Secondary objectives of this study were to determine if temporary androgen suppression by degarelix reduces time to clinical improvement, inpatient mortality, length of hospitalization, duration of intubation for mechanical ventilation, time to achieve a temperature within reference range, or the maximum severity of COVID-19 illness. We report final data analysis from the HITCH trial, a phase II, placebo-controlled, double-blind randomized clinical trial of degarelix plus standard care vs placebo plus standard care in male veterans hospitalized owing to COVID-19 after a planned interim analysis resulted in termination of the trial owing to futility.

## Methods

The HITCH randomized clinical trial was approved by the Department of Veterans Affairs (VA) Central Institutional Review Board and monitored by the VA Clinical Science Research and Development Data Monitoring Committee. Informed consents were obtained from all patients by study investigators or approved study personnel at each participating VA medical center. Both oral and written consents were allowed. This report follows the Consolidated Standards of Reporting Trials (CONSORT) reporting guideline.

### Study Design

The HITCH Trial was a multicenter, phase II, randomized clinical trial of standard care plus degarelix compared with standard care plus placebo to improve the clinical outcomes of male veterans who had been hospitalized owing to COVID-19.^14^ The trial was conducted at 14 VA medical centers and funded by the VA Office of Research and Development. Patients were enrolled from July 22, 2020, to April 8, 2021. The VA Cooperative Studies Program Coordinating Center (CSPCC) at Perry Point, Maryland, provided statistical and administrative support. The trial protocol, statistical analysis plan, and trial amendments are detailed in Supplement 1.

### Participants

Inclusion and exclusion criteria were reported previously,^14^ and updated in eTable 1 in Supplement 2. In brief, the trial enrolled men, aged 18 years and older who had a disease severity from COVID-19 that warranted hospitalization for supportive care but not invasive mechanical ventilation. Male veterans who were hospitalized or were in the process of being admitted to an acute care ward from the outpatient setting or emergency department and had a positive test result for SARS-CoV-2 on nasopharyngeal swab based on an approved reverse transcription–polymerase chain reaction assay were consented and screened. Participants completed screening within 72 hours of informed consent. Race and ethnicity was obtained from the electronic health record and included per VA policy for randomized clinical trials.

### Interventions and Randomization

Participants were centrally randomized 2:1 to degarelix plus standard care vs placebo plus standard care. Randomization was stratified by age (<65 years vs ≥65 years), history of hypertension, and disease severity score (3 vs 4-5) on the modified 7-category ordinal scale of clinical status of hospitalized patients with influenza. Patients with severity scores less than 3 or greater than 5 were excluded. Participants, investigators, and treating physicians were blinded to treatment assignment. Unblinding for emergency situations was managed through a 24-hour a day emergency call service by the VA CSPCC; no unblinding occurred during the trial. Participants assigned to the degarelix group received 1 dose of degarelix 240 mg subcutaneously in the periumbilical area. Participants in the placebo group received an equal volume of saline. The degarelix or placebo were administered within 24 hours of randomization. Standard care included all treatments that would be applied irrespective of patient enrollment and included supplemental oxygen, antibiotics, vasopressor support, peritoneal dialysis or hemodialysis, intravenous fluids, remdesivir, convalescent plasma (through expanded access programs), and dexamethasone. Off-label use of other agents or interventions was allowed, but formal enrollment to another investigational study was prohibited.

Degarelix was selected among the numerous FDA-approved drugs that target the AR owing to its rapid effect on circulating testosterone, safety profile, reversibility, and availability. Most patients achieve a reduction in circulating testosterone to less than 50 ng/dL within 48 hours after a loading dose of degarelix.^15^ A rapid suppression of the AR transcriptional output was desirable for this trial in hospitalized patients. In contrast, the FDA-approved potent AR antagonists (eg, enzalutamide, apalutamide, darolutamide) achieve steady state concentrations in the serum over 1 to 4 weeks and thus seemed inappropriate, given the discordance between the speed of viral multiplication during the acute phase of infection and the time needed for downregulation of TMPRSS2 proposed to reduce viral entry and severity of COVID-19.

### Study Procedures

There was no dose adjustment to degarelix since it was a single, 1-time subcutaneous administration. Degarelix or placebo was administered within 60 minutes of reconstitution. Electrocardiogram was performed at the time of screening to exclude patients with corrected QT interval prolongation at baseline. Laboratory studies for the purposes of safety assessments were performed at the local VA laboratories and included routine complete blood counts, blood chemistry, liver function, cardiac function, and inflammatory laboratory tests. Clinical status was evaluated daily. Total serum testosterone was checked at screening and, if still hospitalized, at days 8, 15, and 30. Adverse events (AEs) were assessed daily during hospitalization, and after discharge, at days 30 and 60. Data for participants at each site were collected locally and entered into an electronic data capture system maintained and managed by the VA CSPCC.

### Outcome Measures

The primary end point was a composite of mortality, need for ongoing hospitalization, or requirement for mechanical ventilation (including extracorporeal membrane oxygenation) at day 15 after randomization. Secondary end points were the composite end point at 30 days after randomization, time to clinical improvement (as defined by a decline of 2 categories or more from the baseline on the modified 7-category ordinal scale of clinical status of hospitalized influenza patients) or discharge, inpatient mortality, duration of hospitalization from time of randomization, duration of intubation for mechanical ventilation, time to achieve a temperature within reference range, and the maximum severity of COVID-19 illness.

### Sample Size

The sample size for the study was estimated based on a superiority trial design with an effect size for the primary end point of 42%. To achieve 90% power of detecting the expected 42% reduction between degarelix and placebo groups, using a 2-sided 2 proportions test with a significance level of .05, required a total of 186 evaluable participants (ie, 124 evaluable patients in the degarelix group and 62 evaluable patients in the placebo group). Based on an assumed 5% attrition rate, 198 participants were required (ie, 132 in the degarelix group and 66 in the placebo group) to achieve statistical significance at α = .05 and 90% power. A midterm interim analysis of the primary end point was planned when approximately half of the required participants would complete their trial participation. If the planned interim analysis of the primary end point indicated that the null hypothesis could be rejected with a boundary value of 2.77 (standardized *Z* > 2.77 or <−2.77) at an α = .006 or accepted with a boundary value of 0.44 (−0.44 ≤ standardized *Z* ≤ 0.44) based on O’Brien and Fleming criteria,^16^ the study would be recommended for trial termination either for efficacy or for futility, respectively.

### Data Safety Monitoring

The VA Clinical Sciences Research and Development centralized Data Monitoring Committee monitored this study. AEs of special interest include cardiac arrhythmias and thromboembolic events that may result from androgen suppression. Specifically, these were cardiac arrhythmias and thromboembolic complications of grades 3 to 5 (Common Terminology Criteria for Adverse Events version 5.0). Nonserious AEs related to the study intervention were reported through the electronic data capture system. Expedited reporting of AEs of special interest (thromboembolic complications or cardiac arrhythmias of grades 3-5) and serious AEs underwent daily review and reports were generated for regular planned weekly teleconference calls among the study investigators. Stopping rules were to be applied if unbalanced toxic effects signals were detected at 25% threshold in the active group at an α = .01.

### Statistical Analysis

The primary analysis was performed to test the null hypothesis of no difference in composite outcome of mortality, need for ongoing hospitalization, and mechanical ventilation at 15 days after randomization between treatment groups according to assigned treatment (intention to treat analysis). Statistical tests were 2-sided, and the primary outcome was tested at 5% level of significance. Secondary end points included in the data analysis were the composite end point at 30 days after randomization, time to clinical improvement, inpatient mortality, length of hospital stay, length of intubation for mechanical ventilation, time to temperature within reference range, and the maximum severity of COVID-19. The secondary end point analyses were adjusted for multiplicity with a α = .0071 for each end point. Time to clinical improvement was defined by the time required for a decline of 2 categories or more from the baseline on the modified 7-category ordinal scale of clinical status of hospitalized patients with influenza or hospital discharge, whichever came first. Survival analysis techniques were used to analyze the time-to-event data for this end point. Patients whose conditions worsened or died or withdrew from the study without clinical improvement were censored. For mortality end point data analysis, the treatment effect was analyzed initially with the Pearson χ^2^ test and later by logistic regression by taking the predefined potential prognostic factors (eg, age, history of hypertension, and history of chronic obstructive pulmonary disorder [COPD]) into account. The length of hospital stay data was analyzed as medians (IQRs), and Wilcoxon tests, a nonparametric method, was performed to compare the medians of the length of hospital stay between treatment groups. In addition, a quantile regression was used to test the treatment effect on the time until the clinical event adjusted for prognostic factors. For the length of intubation for mechanical ventilation data analysis, similar nonparametric analyses were performed as for the length of hospital stay outcome. Time to temperature within reference range was analyzed using log-rank test and Cox proportional hazard model. For the maximum severity of COVID-19 illness data analysis, Pearson χ^2^ test was performed. Given the end point is also an ordinal variable, Cochran-Armitage testing was performed to test the ordinal trend tendency. In addition to the frequency analysis, proportional odds logistic regression was also performed, by adjusting for age, hypertension, COPD, and the baseline influenza scale. SAS statistical software version 9.4 (SAS Institute) was used to conduct all the statistical analyses. Data were analyzed from August 9 to October 15, 2021.

## Results

### Participants

In the HITCH trial, a total of 2154 potential participants were assessed for eligibility at 14 VA medical centers. Of those screened, 130 veterans provided consent for the trial and 101 consented veterans were found to be eligible for randomization (Figure; eTable 2 in Supplement 2). Finally, after 5 eligible participants declined to be randomized, 96 veterans were randomized, in 2 treatment arms at a 2:1 ratio, with 62 veterans in the degarelix and 34 veterans in the placebo group. The median (range) age was 70.5 (48-85) years (Table 1). A total of 68 veterans (70.8%) were 65 years or older. There were 1 American Indian or Alaska Native veteran (1.0%), 2 Asian veterans (2.1%), 38 Black veterans (39.6%), 1 Pacific Islander veteran (1.0%), and 45 White veterans (46.9%), and 14 veterans (14.6%) identified as Hispanic or Latino. Baseline comorbidities included COPD (15 veterans [15.6%]), hypertension (75 veterans [78.1%]), cardiovascular disease (27 veterans [28.1%]), asthma (12 veterans [12.5%]), and diabetes (49 veterans [51.0%]). For all participants, the mean (SD) testosterone level at the baseline was 159.5 (152.1) ng/dL. Regarding the baseline disease severity, 20 veterans (20.8%) were at level 3 (requiring hospitalization but not supplemental oxygen), 59 veterans (51.0%) were at level 4 (requiring supplemental oxygen), and 27 veterans (28.1%) were at level 5 (requiring high-flow oxygen and or noninvasive mechanical ventilation). Fever at baseline was uncommon among the randomized participants (11 veterans [11.5%]). Most participants lived at home prior to admission (91 veterans [94.8%]). Few veterans lived in retirement homes (3 veterans [3.1%]) or nursing homes (1 veteran [1.0%]).

**Figure.**
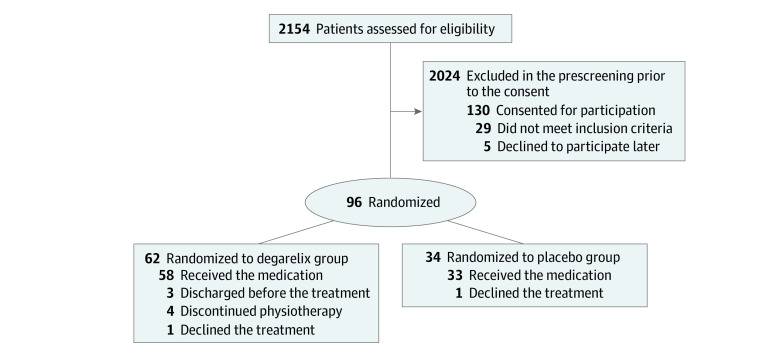
Patient Recruitment Flowchart Complete reasons for exclusion are in eTable 2 in Supplement 2.

**Table 1.** Demographic and Baseline Characteristics

Characteristic	Patients, No. (%)		
	Degarelix (n = 62)	Placebo (n = 34)	Total (N = 96)
Age, y			
Mean (SD)	68.8 (8.6)	68.1 (8.2)	68.5 (8.4)
≥65	45 (72.6)	23 (67.6)	68 (70.8)
Married, in a civil union, or partnered	37 (59.7)	17 (50.0)	54 (56.3)
Hispanic or Latino ethnicity	12 (19.4)	2 (5.9)	14 (14.6)
Race			
American Indian or Alaskan Native	1 (1.6)	0	1 (1.0)
Asian American	2 (3.2)	0	2 (2.1)
Black	23 (37.1)	15 (44.1)	38 (39.6)
Pacific Islander	1 (1.6)	0	1 (1.0)
White	28 (45.2)	17 (50.0)	45 (46.9)
Other^a^	7 (11.3)	2 (5.9)	9 (9.4)
Fever	9 (14.5)	2 (5.9)	11 (11.5)
Testosterone, mean (SD), ng/dL	165.3 (174.7)	148.6 (98.3)	159.5 (152.1)
LOS prior to randomization, median (IQR), d	3 (2-5)	3 (2-5)	3 (2-5)
Severity score at baseline			
3: Hospitalization, not requiring supplemental o_2_	13 (21.0)	7 (20.6)	20 (20.8)
4: Hospitalization, requiring supplemental o_2_	28 (45.2)	21 (61.8)	49 (51.0)
5: Hospitalization, requiring nasal high-flow o_2_ therapy and/or noninvasive mechanical ventilation	21 (33.9)	6 (17.6)	27 (28.1)
Preadmission residence			
Retirement home	3 (4.8)	0	3 (3.1)
Nursing home	0	1 (2.9)	1 (1.0)
Own residence	59 (95.2)	32 (94.1)	91 (94.8)
Other	0	1 (2.9)	1 (1.0)
Medical history			
COPD	11 (17.7)	4 (11.8)	15 (15.6)
Hypertension	49 (79.0)	26 (76.5)	75 (78.1)
Cardiovascular disease	19 (30.6)	8 (23.5)	27 (28.1)
Asthma	7 (11.3)	5 (14.7)	12 (12.5)
Diabetes	35 (56.5)	14 (41.2)	49 (51.0)
Use of angiotensin converting enzyme inhibitors	14 (22.6)	14 (41.2)	28 (29.2)
Use of supplemental o_2_	7 (11.3)	2 (5.9)	9 (9.4)

Abbreviations: COPD, chronic obstructive pulmonary disease; LOS, length of stay; o_2_, oxygen.

SI conversion factor: To convert testosterone to nanomoles per liter, multiply by 0.0347.

^a^ Other race is reported as noted in electronic medical records, and no further information was given.

### Primary Outcome

There was no statistically significant difference between groups for the primary composite end point. At 15 days after randomization, 19 veterans (30.6%) in the degarelix group were either still hospitalized, had died, or required mechanical ventilation, compared with 9 veterans (26.5%) in the placebo group (adjusted odds ratio [aOR], 1.19; 95% CI, 0.46-3.06; *P* = .67) (Table 2).

**Table 2.** Effect of Degarelix Treatment on Clinical Outcomes Among Hospitalized Patients With COVID-19

Outcome	No. (%)		Adjusted effect estimate (95% CI)^a^	*P* value^b^
	Degarelix (n = 62)	Placebo (n = 34)		
Primary				
Composite end point at day 15^c^	19 (30.6)	9 (26.5)	1.19 (0.46 to 3.06)	.67
Secondary				
Composite end point at day 30^c^	15 (24.2)	7 (20.6)	1.22 (0.44 to 3.42)	.69
All-cause mortality	11 (17.7)	7 (20.6)	0.78 (0.27 to 2.31)	.73
All-cause mortality prior to discharge	11 (17.7)	6 (17.6)	0.95 (0.31 to 2.92)	.99
Length of hospital stay, median (IQR), d	6 (3-9)	5 (5-8)	0.00 (−2.03 to 4.11)	.84
Duration of mechanical ventilation, median (IQR), d^d^	0 (0-0)	0 (0-0)	NA	.75
Use mechanical ventilation	13 (21.0)	6 (17.6)	NA	.70
Time to clinical improvement, median (IQR), d	7 (4-15)	5.5 (5-15)	0.93 (0.58 to 1.49)	.88
Time to temperature within reference range, median (IQR), d^e^	3 (1-3)	5 (2-18)	2.30 (0.72 to 7.39)	.10
Maximum disease severity				
3: Hospitalization, not requiring supplemental o_2_	8 (12.9)	4 (11.8)	0.82 (0.33 to 2.00)	.43
4: Hospitalization, requiring supplemental o_2_	26 (41.9)	20 (58.8)		
5: Hospitalization, requiring nasal high-flow o_2_ therapy and/or noninvasive mechanical ventilation	14 (22.6)	4 (11.8)		
6: Hospitalization, requiring extracorporeal membrane oxygenation and/or invasive mechanical ventilation	3 (4.8)	0		
7: Death	11 (17.7)	6 (17.6)		

Abbreviations: NA, not applicable; o_2_, oxygen.

^a^ Treatment effects of Degarelix estimated from multiple regressions adjusted for age, history of hypertension, and history of COPD. Regression model for Maximum Severity outcome also adjusted for baseline severity.

^b^ *P* values from unadjusted analysis.

^c^ The composite end point was mortality, ongoing need for hospitalization, or mechanical ventilation.

^d^ Length of mechanical ventilation imputed to maximum length (50 days) for patients who died while receiving mechanical ventilation or who were receiving mechanical ventilation but date removed was unknown. Length of mechanical ventilation imputed to 0 for patients who never received mechanical ventilation. Four Patients who died but who had not received mechanical ventilation prior to death were excluded.

^e^ Patients with temperature within reference range at randomization were excluded from the analysis.

### Secondary and Other Outcomes

At 30 days after randomization, the composite end point of ongoing hospitalization, mortality, or having required mechanical ventilation was met by 15 veterans (24.2%) in the degarelix group, compared with 7 veterans (20.6%) in the placebo group (aOR, 1.22; 95% CI, 0.44-3.42; *P* = .69) (Table 2). Additionally, 11 veterans (17.7%) in the degarelix group and 6 veterans (17.6%) in the placebo group died before discharge (aOR, 0.95; 95% CI, 0.31-2.92; *P* = .99). One additional patient in the placebo group died after discharge but within 30 days of randomization. The maximum disease severity after randomization did not differ between degarelix and placebo groups (aOR, 0.82; 95% CI, 0.33-2.00; *P* = .43) (Table 2). The median (IQR) length of stay was 6 (3-9) days for the degarelix group and 5 (5-8) days for the placebo group (*P* = .84). There was no difference in time to temperature within reference range between the treatment groups. For patients hospitalized at day 8, mean (SE) serum testosterone levels were 40.4 (13.18) ng/dL in the degarelix group and 119.6 (23.36) ng/dL in the placebo group (eTable 3 in Supplement 2).

### Adverse Events

There were no differences between the degarelix and placebo groups in the overall rates of AEs (13 veterans [21.0%] vs 8 veterans [23.5%]) or serious AEs (19 veterans [30.6%] vs 13 veterans [32.4%]). There were no differences in cardiovascular AEs between the groups (2 veterans [3.2%] vs 3 veterans [8.8%]). Moreover, there were no statistically significant different rates of any specific AE between groups. The most common AEs were related to worsening of COVID-19. No worrisome safety signals were noted during the data monitoring (eTable 4 and eTable 5 in Supplement 2).

## Discussion

This randomized clinical trial tested the effect of testosterone suppression on outcomes among men hospitalized with COVID-19. Importantly, there were no significant differences in any clinical end points or AEs between degarelix and placebo groups. This study found that medical castration did not reduce the severity of COVID-19 among hospitalized men, and the phase 2 trial was stopped for futility.

Given the evidence in support of the underlying hypothesis, why did suppression of serum testosterone fail to improve clinical outcomes of hospitalized men with COVID-19? First, suppression of testosterone is itself a physiologic response to acute, critical illness,^17,18^ and serum testosterone has indeed been reported to be markedly reduced in patients who are severely ill with COVID-19,^19^ although it is not clear if associations between low testosterone and incidence, severity, and mortality from COVID-19 are associative or causative. In the HITCH study, total testosterone decreased in both treatment groups, though markedly more so in the degarelix group. Of note, baseline testosterone was low in both groups. It is plausible that the reduction of testosterone in the placebo group in response to COVID-19 illness reached a threshold to affect physiology. Accordingly, the additional testosterone reduction by degarelix may not have yielded further inhibition of viral coreceptor expression, although we did not directly analyze TMPRSS2 or ACE2 expression in this trial.

Second, the timing of downregulation of TMPRSS2 may have limited the effect of androgen deprivation therapy on the severity of illness. The stage of the disease at which point patients enrolled in the HITCH study may no longer have been dependent on ongoing viral infection but rather on a hyperactivated immune response that results in end-organ damage. If this were the case, then reduction of the expression of the viral coreceptors would not be expected to have had an impact on the course of the disease.

Third, androgens are immunosuppressive^20^ and have been shown to inhibit innate and adaptive immunity, including T-cells.^21,22^ As such, heightened suppression of serum testosterone could have further activated an already hyperactivated immune system, which could counter any beneficial effect that may have been mediated by suppression of viral coreceptors.

Fourth, in men, serum estrogen is derived from testosterone, and, consequently, testosterone suppression is expected to result in lower absolute concentrations of serum estrogen. Estrogens have been reported to suppress the expression of viral coreceptors.^23,24^ In the context of medical castration, reduced estrogen concentrations could counterbalance the effects of reduced testosterone on viral coreceptor expression. In designing the HITCH trial, a third group evaluating estrogen therapy was considered during the design phase but ultimately abandoned owing to potential thromboembolic complications.

Finally, despite preclinical data, it is also possible that androgens do not regulate TMPRSS2 and ACE2 in relevant tissues to an extent that is targetable by antiandrogen therapy to ameliorate severity of COVID-19 in patients. Additionally, the timing of androgen suppression after the diagnosis and subsequent hospitalization for COVID-19 may be too late to affect the outcomes of patients. It is possible that earlier use of androgen-directed therapies during the initial infection and viral replication may be a more effective strategy.

Although medical castration and AR antagonists both reduce transcriptional output of the AR and would be expected to reduce expression of the viral coreceptors, the downstream physiologic effects of these 2 AR-targeting strategies differ. AR antagonists result in an increase in circulating testosterone and estrogen in men.^25^ The increase in testosterone could in principle have an immunosuppressive effect, which may have a salutary effect in patients with established COVID-19 infection. Moreover, the rise in estrogen concentrations may further suppress viral coreceptor expression.^23,24^ Repurposed estrogenic drugs are now in trials as potential therapy for COVID-19. As such, discordant efficacy between luteinizing hormone releasing hormone antagonists and AR antagonists for COVID-19 is conceivable.

Interestingly, a study by McCoy et al^10^ reported results of a clinical trial testing the AR antagonist proxalutamide vs placebo in outpatients with COVID-19. In that trial, men with confirmed COVID-19 but not requiring hospitalization were randomized to a 7-day course of proxalutamide or placebo. At 30 days, fewer patients in the proxalutamide group than the placebo group were hospitalized owing to COVID-19. A separate study by Cadegiani et al^11^ reported that proxalutamide accelerated viral clearance compared with placebo in outpatients. The efficacy of proxalutamide on amelioration of COVID-19 severity in hospitalized patients has yet to be reported in detail.

### Limitations

This study has some limitations. While no signal of efficacy for degarelix was observed in this trial and the trial was terminated based on prespecified conditions for futility, we cannot exclude the possibility of a small effect size below the limit of detection. A study by McCoy et al^10^ reported that antiandrogen therapy reduces hospitalization for COVID-19. We cannot exclude the possibility that androgen suppression could potentially be active against COVID-19 in patients prior to hospitalization. Another limitation is that standard care for COVID-19 in both groups was determined by the treating physicians; thus, it is possible that differences in standard care over the course of the trial enrollment period and between enrollment sites could influence outcomes.

## Conclusions

The HITCH randomized clinical trial demonstrated that androgen suppression via temporary medical castration did not improve the clinical outcome of hospitalized men with COVID-19. Further clinical investigation of androgen suppression in this specific clinical setting is not warranted.

## Notes

Supplement 1.
Trial Protocol, Statistical Analysis Plan, and Protocol Amendments

Supplement 2. **eTable 1.** Inclusion and Exclusion Criteria **eTable 2.** Reasons for Study Exclusion **eTable 3.** Testosterone Levels After Degarelix Treatment **eTable 4.** Adverse Events for Randomized Participants by Treatment Groups **eTable 5.** Serious Adverse Events for Randomized Participants by Treatment Groups

Supplement 3. Data Sharing Statement
