# Supplementary material for: Effect of Androgen Suppression on Clinical Outcomes in Hospitalized Men With COVID-19: The HITCH Randomized Clinical Trial
Source: JAMA Netw Open. 2022 Apr 19;5(4):e227852. doi: 10.1001/jamanetworkopen.2022.7852 (PMC9020208; doi:10.1001/jamanetworkopen.2022.7852)
Supplement: Supplement 1. — Trial Protocol, Statistical Analysis Plan, and Protocol Amendments [file jamanetwopen-e227852-s001.pdf]

Hormonal **I**ntervention for the **T**reatment in Veterans with **C**OVID-19 Requiring **H**ospitalization **(HITCH)**: A Multicenter, Phase 2 Randomized Controlled Trial of Best Supportive Care (BSC) vs BSC plus Degarelix

Principal Investigator:

Matthew B. Rettig, M.D.
Chief, Hematology/Oncology, VA Greater Los Angeles Healthcare System (GLA)
Professor of Medicine and Urology
Medical Director, Prostate Cancer Program
Institute of Urologic Oncology
David Geffen School of Medicine at UCLA

Study Drugs: Degarelix

Funding Agency: VA CSR&D

Protocol Version: 5.5., February 9, 2021

Proprietary and Confidential

The information in this document is considered privileged and confidential, and may not be disclosed to others except to the extent necessary to obtain Institutional Review Board approval and informed consent, or as required by Federal and State laws. Persons to whom this information is disclosed should be informed that this information is privileged and confidential and should not be further disclosed.

PRINCIPAL INVESTIGATOR APPROVAL OF PROTOCOL

The HITCH Trial will be carried out in accordance with the protocol, the principles of good clinical practice and in accordance to Veteran's Affairs Medical Centers (VAMC), Research & Development (R&D) guidelines and regulations as well as national regulatory requirements.

The undersigned has read and approve this protocol and agree to its content:

Principal Investigator:

Matthew Rettig, MD____	____________________	____________________
Name	*Signature*	*Date*

PROTOCOL SITE INVESTIGATOR SIGNATURE SHEET

I have read the HITCH Trial protocol, including all appendices, and I agree that it contains all necessary details for my staff and I to conduct this protocol as described. I will personally oversee protocol conduct as outlined herein.

I will provide all study personnel under my supervision with copies of the protocol and access to all information provided for the study.

Before commencement of this study at my clinical facility, the Central Institutional Review Board (IRB)/ethics committee must approve this protocol. I agree to make all reasonable efforts to adhere to the HITCH Trial protocol.

I, or my designee, agree to be present at all site visits and Investigator meetings. I will ensure the presence of relevant study personnel under my supervision at these visits and meetings. I will conduct the study according to the procedures specified herein, and according to the principles of Good Clinical Practice (GCP), VAMC research and development guidelines and regulations:

Signature Site PI: ___________________________

Printed Name of Site PI: _________________________

Site Location: _________________________

Date: _________________________

HITCH Trial Protocol Version and Amendment Tracking

Version Number	Date
HITCH Protocol, v1.0, 20200407	April 7, 2020
HITCH Protocol, v2.0, 20200420	April 20, 2020
HITCH Protocol, v2.0, 20200423	April 23, 2020
HITCH Protocol, v2.0, 20200427	April 27, 2020
HITCH Protocol, v3.0, 20200505	May 5, 2020
HITCH Protocol, v4.0, 20200525	May 25, 2020
HITCH Protocol, v4.1, 20200525	May 27, 2020
HITCH Protocol, v5.1, 20200618	June 18, 2020
HITCH Protocol, v5.2, 20200714	July 14, 2020
HITCH Protocol, v5.3, 20200804	August 4, 2020
HITCH Protocol, v5.4, 20201103	November 3, 2020
HITCH Protocol, v.5.4.5, 20210115	January 15, 2021
HITCH Protocol, v.5.5, 20210209	February 9, 2021

STUDY TEAM ROSTER

GLA

Principal Investigator:

Matthew Rettig, M.D.
Chief, Division of Hematology-Oncology, GLA
Professor of Medicine and Urology
David Geffen School of Medicine at UCLA
VA Greater Los Angeles HCS
11301 Wilshire Blvd
Building 500, Room 4237
Los Angeles, CA 90073
Ph: 310-268-3622; mobile: 310-569-1767 (best contact)
e-mail: matthew.rettig@va.gov; mrettig@mednet.ucla.edu
UCLA pager: 310-825-6301 (pager 14320)

GLA Co-Investigators:

Nicholas Nickols, MD, PhD
Staff Physician, Radiation Oncology
Associate Professor
Department of Radiation Oncology
David Geffen School of Medicine at UCLA
Ph: 310-268-3390
Cell: 818-421-5077
Nicholas.nickols@va,gov; nnickols@mednet.ucla.edu
VA pager: 310-478-3711 (pager 55053)

Matthew Goetz, MD
Chief, Infectious Diseases, GLA
Professor of Medicine
David Geffen School of Medicine at UCLA
Ph: 310-478-3711 x 44716
Matthew.goetz@va.gov

Christopher Graber, MD
Staff Physician, Division of Infectious Diseases, GLA
Assistant Professor Medicine
David Geffen School of Medicine at UCLA
Ph: 310-478-3711 x40275
Cell: 310-295-8376
Christopher.Graber@va.gov

Debika Bhattacharya, MD
Staff Physician, Division of Infectious Diseases, GLA
Assistant Clinical Professor
David Geffen School of Medicine at UCLA

Ph: 310-925-0137
DebikaB@mednet.ucla.edu

Guy Soo Hoo, MD
Chief, Pulmonary and Critical Care, GLA
Professor of Medicine
David Geffen School of Medicine at UCLA
Ph: (310)268-3021
Mobile: (310)729-3273
guysoohoo@va.gov; gsoohoo@ucla.edu

Greg Orshansky, MD, FACP
Chief, Clinical Informatics and Data Analytics
Chief Health Informatics Officer
VA Greater Los Angeles Healthcare Systems
Ph: 310 478-3711 x43988
Greg.Orshansky@va.gov

GLA Study Coordinators

Samantha Tran
VA GLAHS
11301 Wilshire Blvd
Building 304, 2nd Floor, Room 2-304Ph: 310-478-3711 x44917
Samantha.Tran@va.gov

Leila Ghayouri
VA GLAHS
11301 Wilshire Blvd
Building 304, 2nd Floor, Room 2-304
Los Angeles, CA 90073
Ph: 310-478-3711 x44386

Leila@brentwoodresearch.orgMichelle Geelhoed, PA
VA GLAHS
11301 Wilshire Blvd
Building 500, 4 East, Room 4237
Los Angeles, CA 90073
Ph: 310-478-3711 x40373
Michelle.geelhoed@va.gov

Sonny Tsai
VA GLAHS
11301 Wilshire Blvd
Building 304, 2nd Floor, Room 2-304
Los Angeles, CA 90073
Ph: 310-478-3711 x44567

Sonny.Tsai@va.gov

Manhattan VA

Manhattan Principal Investigator:

Daniel Becker, MD, MPH
Section Chief, Hematology-Oncology, VA-NYHHS, Manhattan Campus
Assistant Professor of Medicine
NYU School of Medicine
Daniel.Becker@nyulangone.org
Daniel.Becker2@va.gov
212-686-7500 x3303

Manhattan Co-Investigator:

Jun-Chieh (James) Tsay, MD, MS
Staff Physician, Division of Pulmonary and Critical Care, VA-NYHHS, Manhattan Campus
Assistant Professor of Medicine
NYU School of Medicine
Jun-Chieh.Tsay@nyulangone.org

Manhattan Study Coordinators:

Melissa Diamond
Program Manager
Ph: 22-686-7500 x3924
Cell: 917-821-9106 (best contact number)
Melissa.diamond@va.gov

Asha George
Research Coordinator
212-686-7500 x3922
Asha.George@va.gov

Brooklyn VA

Brooklyn Principal Investigator:

Daniel Becker, MD, MPH
Section Chief, Hematology-Oncology, VA-NYHHS, Manhattan Campus
Assistant Professor of Medicine
NYU School of Medicine
212-686-7500 x3303
Daniel.Becker@nyulangone.org
Daniel.Becker2@va.gov

Brooklyn Co-Investigators:

Mohammad Al-Ajam, MD, FCCP
Section Chief, Pulmonary/Critical Care/Sleep Simulation
Department of Medicine

Ph: 718-630-3722
Cell: 917-859-9604
Mohammad.Al-Ajam@va.gov

Pooja Belligund, MD
Attending Physician, Pulmonary/Critical Care
Department of Medicine
Ph 718-836-6600 x6080
Pooja.Belligund@va.gov

Puget Sound VA

Puget Sound Principal Investigator

Bruce Montgomery, MD
Attending Physician, Division of Hematology-Oncology
Ph: 206-598-0860
rbmontgo@uw.edu

Puget Sound Co-Investigator

Elahe Mostaghel, MD
Attending Physician, Division of Hematology-Oncology
Ph: 206-277-1657
emostagh@fredhutch.org
elahe.mostaghel@va.gov

Puget Sound Coordinator

Carlie Sulpizio
(206) 762-1010 Ext 6-7190
Carlie.Sulpizio@va.gov

Philadelphia VA Medical Center

Philadelphia Principal Investigator

Kyle Robinson, MD
Staff Physician, Hematology and Oncology
Ph: 267-239-4924
Kyle.Robinson3@va.gov

Charleston VA Medical Center

Charleston Principal Investigator

John Huggins, MD
Staff Physician, Pulmonology

Ph: 843-789-6392
John.Huggins@va.gov

Long Beach VA Medical Center

Long Beach Principal Investigator

Ellis Levin, MD
Chief, Endocrinology
Ph: 562-826-8000 x14147
Ellis.Levin@va.gov

Houston VA Medical Center

Houston Principal Investigator

Reina Villareal, MD
Staff Physician, Endocrinology
Ph: 713-794-7534
Reina.Villareal@va.gov

Phoenix VA Medical Center

Phoenix Principal Investigator

Samuel Aguayo, MD
Staff Physician, Endocrinology
Ph: 602-277-5551 x7808
Samuel.Aguayo@va.gov

Memphis VA Medical Center

Memphis Principal Investigator

Muthiah Muthiah, MD
Staff Physician, Pulmonology
Ph: 901-523-8990 x5050
Muthiah.Muthiah@va.gov

Miami VA Medical Center

Miami Principal Investigator

Mehdi S. Mirsaeidi, MD
Staff Physician, Pulmonology
Ph: 305-575-7000 x4594

Mirsaeidi.Mehdi@va.gov

North Texas VA Medical Center

North Texas Principal Investigator

Roger Bedimo, MD
Chief, Infectious Disease
Ph: 241-857-0397
Roger.Bedimo@va.gov

Palo Alto VA Medical Center

Palo Alto Principal Investigator

Brian T. Dietrich, MD
Staff Physician, Hematology
Ph: 650-493-5000
Brian.Dietrich2@va.gov

Cooperative Studies Program

Kousick Biswas, Ph.D.
Director
Cooperative Studies Program Coordinating Center
Office of Research and Development
U.S. Department of Veterans Affairs
VA Medical Center, Perry Point, MD 21902
Associate Professor
Department of Epidemiology and Public Health
School of Medicine, University of Baltimore
Baltimore, Maryland 21201
Kousick.Biswas@va.gov

Zhibao Mi, Ph.D.
Biostastician
Cooperative Studies Program Coordinating Center
Office of Research and Development
U.S. Department of Veterans Affairs
VA Medical Center, Perry Point, MD 21902
Zhibao.Mi@va.gov

Ellen De Matt, MA
Chief Statistical Programmer

Cooperative Studies Program Coordinating Center
Office of Research and Development
U.S. Department of Veterans Affairs
VA Medical Center, Perry Point, MD 21902
Ellen.DeMatt@va.gov

Joseph Tadalan, MS
Chief Database Programmer
Cooperative Studies Program Coordinating Center
Office of Research and Development
U.S. Department of Veterans Affairs
VA Medical Center, Perry Point, MD 21902
Joseph.Tadalan@va.gov

Erin Norman, MSW
Project Manager
Cooperative Studies Program Coordinating Center
Office of Research and Development
U.S. Department of Veterans Affairs
VA Medical Center, Perry Point, MD 21902
Leslie.Norman@va.gov

Daniel Briones, BA
Clinical Data Manager
Cooperative Studies Program Coordinating Center
Office of Research and Development
U.S. Department of Veterans Affairs
VA Medical Center, Perry Point, MD 21902
Daniel.Briones@va.gov

Christina E. Clise, Pharm.D.
Clinical Research Pharmacist
VA CSPCRPCC
Office of Research and Development
U.S. Department of Veterans Affairs
Albuquerque, NM 87106
Christina.Clise@va.gov

Zachary Taylor, BS
Computer Scientist
VA CSPCRPCC
Office of Research and Development
U.S. Department of Veterans Affairs
Albuquerque, NM 87106
Zachary.Taylor@va.gov

Jeffrey Huminik, BA
Project Manager
VA CSPCRPCC
Office of Research and Development
U.S. Department of Veterans Affairs
Albuquerque, NM 87106
Jeffrey.Huminik@va.gov

SYNOPSIS

Study Title:

Hormonal **I**nterventions for the **T**reatment in Veterans with **C**OVID-19 Requiring **H**ospitalization **(HITCH)**: A Multicenter Phase 2 Randomized Controlled Trial of Best Supportive Care (BSC) vs BSC plus Degarelix.

Rationale

SARS-CoV-2 entry into lung epithelium requires the TMPRSS2 cell surface receptor. Pre-clinical and correlative data in humans strongly suggest that anti-androgenic therapies can reduce the expression of TMPRSS2 on lung epithelium. Accordingly, we hypothesize that therapeutic targeting of androgen receptor signaling will suppress viral infection and thereby ameliorate the severity of symptomatic COVID-19.

Purpose:

The purpose of this study is to determine if temporary androgen suppression improves the clinical outcomes of Veterans who are hospitalized to an acute care ward due to COVID-19.

Target Population:

Male Veterans, ≥18 - ≤ 85 years old (patients > 85 can be enrolled if there is no history of chronic obstructive pulmonary disease (COPD), asthma, cardiovascular disease, hypertension, diabetes mellitus or active malignancy), who have been admitted to acute care ward due to COVID-19.

Objectives:

The primary objective of this study is to determine if temporary androgen suppression induced by degarelix improves the clinical outcomes of Veterans who are hospitalized to an acute care ward due to COVID-19 as defined by a reduction in mortality, ongoing need for hospitalization, or requirement for mechanical ventilation/extracorporeal membrane oxygenation (ECMO).

The secondary objectives of this study are to determine if temporary androgen suppression induced by degarelix reduces time to clinical improvement, inpatient mortality, length of hospitalization, duration of intubation for mechanical ventilation, time to achieve a normal temperature, or the maximum severity of COVID-19 illness.

Endpoints:

Primary Endpoint:

Determine if degarelix + best supportive care (BSC) as compared to placebo + BSC reduces the composite endpoint of mortality, ongoing need for hospitalization, or requirement for mechanical ventilation/extracorporeal membrane oxygenation (ECMO) at Day 15 after randomization.

Secondary Endpoints:

- Determine if degarelix + best supportive care (BSC) as compared to placebo + BSC reduces the composite endpoint of mortality, ongoing need for hospitalization, or requirement for mechanical ventilation/extracorporeal membrane oxygenation (ECMO) at Day 30 after randomization.
- Determine if degarelix + BSC as compared to placebo + BSC reduces time to clinical improvement as defined by a decline of 2 categories or more from the baseline on the modified 7-category ordinal scale of clinical status of hospitalized influenza patients (influenza scale, see Appendix A).[1] or hospital discharge, whichever comes first.
- Determine if degarelix + BSC as compared to placebo + BSC reduces inpatient mortality.
- Determine if degarelix + BSC as compared to placebo + BSC shortens the duration of hospitalization.
- Determine if degarelix + BSC as compared to placebo + BSC shortens the duration of intubation for mechanical ventilation.
- Determine if degarelix + BSC as compared to placebo + BSC reduces the time to normalization of temperature (T < 37.5℃ for ≥ 48 hours).
- Determine if degarelix + BSC as compared to placebo + BSC reduces the maximum severity of COVID-19 illness based on the influenza severity scale (see Appendix A).

Exploratory Endpoints

- Determine if age, use of angiotensin converting enzyme inhibitors, the duration of pre-hospitalization symptoms, or the presence or absence of hypertension, cardiovascular disease, asthma, diabetes mellitus, or COPD are prognostically associate with clinical outcome defined by the primary endpoint or any secondary endpoint.

- Determine if baseline, Day 8 or Day 15 values for D-dimer, IL-6, LDH, ferritin, total WBC, absolute neutrophil count (ANC), absolute lymphocyte count (ALC), or testosterone are predictive of clinical outcome defined by the primary endpoint or any secondary endpoint.

- Determine if degarelix + BSC as compared to placebo + BSC reduces nasopharyngeal (NP) epithelial or peripheral blood mononuclear cell (PBMC) TMPRSS2 expression, NP and plasma viral load, or serum cytokine (TNFα, IL-1β, IL-6) levels.

- Determine whether germline genomic factors are predictive of clinical outcome defined by the primary endpoint or any secondary endpoint. This analysis is optional and is contingent on obtaining adequate number of samples.

- To determine the temporal onset of anti-viral antibodies.

Design

This is a randomized phase 2, placebo-controlled, double blind clinical trial to compare the efficacy of degarelix + BSC to the placebo + BSC on improving the clinical outcomes of male Veterans who have been hospitalized due to COVID-19. Enrolled patients must have documented infection with SARS-Cov-2 based on a positive reverse transcriptase polymerase chain reaction (RT-PCR) result performed on a nasopharyngeal swab and have a severity of illness of level 3-5 (see Appendix A). Patients will be centrally randomized 2:1 (degarelix + BSC: placebo + BSC) to the study arms. BSC consists of supplemental oxygen, antibiotics, vasopressor support, peritoneal or hemodialysis, antibiotics, intravenous fluids, etc.

Statistical Considerations

Expected Treatment Effects

In a recent antiviral drug trial to treat adults hospitalized with severe COVID-19 published in the New England Journal of Medicine (NEJM) on March 18, 2020 (Cao *et al*. 2020), the results showed a mortality rate of 17%, hospital stay rate of 50%, and ECMO or mechanical intubation rate of 11% among the patients managed with BSC after two-week follow-up. In the current study, we propose a composite endpoint of mortality, hospital stay rate, and ECMO or mechanical intubation at 15 days after randomization. We are expecting to reduce the composite endpoint rate to 35% after the degarelix treatment plus BSC from 60% for patients treated placebo plus BSC.

Sample Size Calculation and Power Analysis

The sample size estimation and power analysis shown in Table 2 are based on the hypothesis testing of the primary endpoint, which is the composite endpoint of mortality, hospital stay rate, and ECMO or mechanical intubation at 15 days after randomization (Day 15).

Based on the composite primary endpoint, we assume that an effect size for the primary endpoint of 42% can be anticipated in the degarelix group. The other assumptions include a three-month accrual time and one-month follow-up time, and 2:1 sample allocation of degarelix: placebo treatment group. The sample size for the study is estimated based on a superiority trial design. To have 90% power of detecting the expected 42% reduction using a two-sided two proportion test with a significance level of 0.05 in the degarelix compared to the placebo group will require 186 evaluable patients total (i.e. 124 evaluable patients in the degarelix group and 62 evaluable patients in the

placebo group). Based on an assumed 5% attrition rate, 198 patients will be required (i.e. 132 in the degarelix group and 66 in the placebo group) to achieve actual statistical significance at alpha level of 0.05 and power of 90%.

Study Schematic

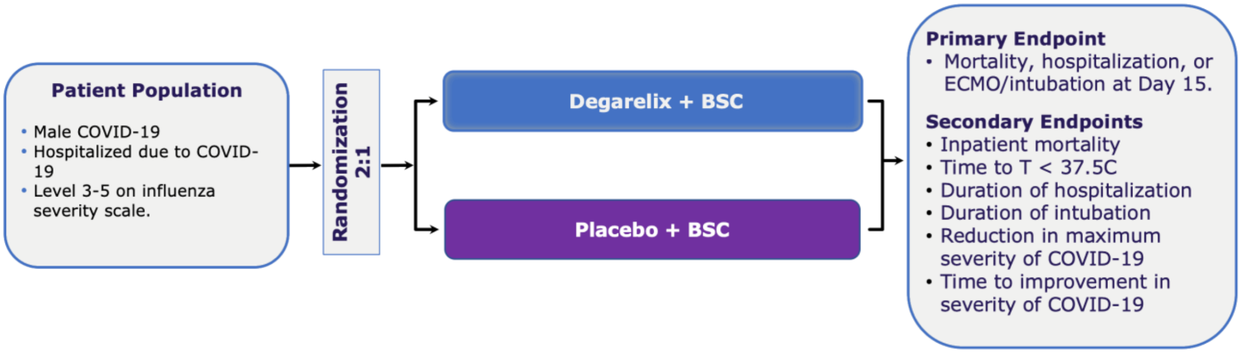

Study Treatments

Patients assigned to the degarelix arm will receive degarelix 240 mg subcutaneously (SQ) in the periumbilical area. The degarelix or matched placebo will be administered within 24 hours of randomization (i.e. within 24 hours of Day 1). Patient in the BSC alone arm will receive placebo. Patients, investigators, and treating physicians will be blinded to treatment assignment (i.e. double blinding).

Screening

Male Veterans who have been hospitalized or are in the process of being admitted to an acute care ward from the outpatient setting or emergency department (ED) and have a positive nasopharyngeal swab for SARS-Cov-2 based on an approved RT-PCR assay will be screened. Patients will complete screening within 72 hours of signing the informed consent form (ICF).

Eligibility Criteria

Inclusion Criteria

1. Male Veterans admitted to a VA hospital.
2. Age ≥ 18
3. Hospitalized on an acute care ward with a diagnosis of COVID-19 contributing to hospitalization.
4. Positive RT-PCR assay for SARS-CoV-2 on a nasopharyngeal swab sample.
5. Severity of COVID-19 illness of level 3, 4 or 5 on the influenza severity scale (see Appendix A) at the time of randomization.

6. The subject (or legally acceptable representative if applicable) must provide informed consent for the trial.

Exclusion Criteria

1. History of severe hypersensitivity to degarelix or any component of their respective formulation
2. History of congenital long QT syndrome or known history of prolonged QT interval OR Fridericia correction formula (QTcF) > 500 msec on electrocardiogram performed at screening.
3. Planned discharge within 24 hours of treatment initiation.
4. Subject is planning to conceive or father children within the projected duration of the study, starting with the screening visit through 120 days after the last dose of study treatment.
1. Ongoing usage of a Class IA or Class III antiarrhythmic agent (see Appendix D). At least 5 half-lives must elapse since any prior use of a Class IA or III antiarrhythmic agent prior to administration of study drug.
5. Baseline electrolyte abnormalities of Grade 3 or higher (based on CTCAE v5.0 criteria). Patients may be included if baseline electrolyte abnormalities are corrected to Grade 2 or lower prior to study drug administration.
6. Myocardial infarction in the past 6 months, severe or unstable angina, or New York Heart Association (NYHA) Class III or IV heart disease.
7. Enrollment in another investigational study within 30 days of Day 1.
8. Known psychiatric or substance abuse disorder that would interfere with the requirements of the trial.
9. Child-Pugh Class C liver disease.
10. Use of any of the following hormonal agents:
    - Androgen receptor antagonists or agonists within 4 weeks of study enrollment,
    - Ketoconazole or abiraterone acetate within 2 weeks of study enrollment,
    - Estrogens or progestins within 2 weeks of study enrollment,
    - Herbal products that contain hormonally active agents within 2 weeks of study enrollment.
    - Any prior use of an LHRH analogue unless a serum total testosterone measured within 30 days of study enrollment is $\geq$ 150 ng/dL.
    - Other hormonal agents listed in Appendix B within one day of study enrollment.
11. Unwilling or unable to comply with the study protocol.
12. Any condition, which in the opinion of the investigator, would preclude participation in the trial.

LIST OF ABBREVIATIONS

AE = adverse event
ALT = alanine aminotransferase
ANC = absolute neutrophil count
AR = androgen receptor
AST = aspartate aminotransferase
BSC = best supportive care
CBC = complete blood count
CDMS = clinical data management system
CFR = Code of Federal Regulations
COPD = chronic obstructive pulmonary disease
CRF = case report form
CSPCC = Cooperative Studies Program Coordinating Center
CTCAE v5.0 = Common Terminology Criteria for Adverse Events version 5.0
CT = computed-tomography
DSMB = Data Safety Monitoring Board
ECMO = extracorporeal membrane oxygenation
eCRF = electronic case report form
eDC = electronic data capture
FDA = Food and Drug Administration
Hgb = hemoglobin
ICF = informed consent form
IL-1β = interleukin-1 beta
IL-6 = interleukin-6
LAR = legally authorized representative
IRB = institutional review board
LDH = lactate dehydrogenase
LFTs= liver function tests
LHRH = luteinizing hormone releasing hormone
MRI = magnetic resonance imaging
NP = nasopharyngeal
PaO_2 = partial pressure of oxygen
QT = electrocardiogram interval from onset of the QRS complex to the end of the T wave representing duration of repolarization
QT_CF = electrocardiogram interval for QT interval corrected by the Fridericia correction formula
PI= principal investigator
SAE = serious adverse event
SaO_2 = oxygen saturation
SQ = subcutaneously
TNFα = tumor necrosis factor alpha

TABLE OF CONTENTS

Page

STUDY TEAM ROSTER 5

SYNOPSIS 13

LIST OF ABBREVIATIONS 18

1. INTRODUCTION 19
2. STUDY OBJECTIVES AND ENDPOINTS 24
3. STUDY POPULATION 26
4. STUDY ENROLLMENT PROCEDURES 28
5. STUDY DESIGN 33
6. STUDY TREATMENT 35
7. STUDY PROCEDURES
8. ADVERSE EVENTS AND SERIOUS ADVERSE EVENTS 43
9. SAFETY MONITORING 47
10. STATISTICAL CONSIDERATIONS 48
11. DATA COLLECTION AND QUALITY ASSURANCE 57
12. PATIENT RIGHTS AND CONFIDENTIALITY 60
13. FUNDING AND INSURANCE 61
14. PUBLICATION OF RESEARCH FINDINGS 61
15. REFERENCES 62
16. APPENDICES 66

1. INTRODUCTION

Background

A novel coronavirus, now termed Severe Acute Respiratory Syndrome Coronavirus 2 (SARS-CoV-2), arose late in 2019.[2] The first confirmed cases occurred in December in Wuhan, Hubei province, China. It now infects people on six continents,

spreading person to person. The World Health Organization (WHO) classified it as a global pandemic on March 11, 2020. As of April 6, 2020, there are more than 1.2 million confirmed cases and more than 70,000 deaths attributed to this virus. Every person on Earth, as well as every United States Veteran, is at risk. This is the emergent public health threat of our time.

SARS-CoV-2 is a singled stranded RNA virus[3] related to severe acute respiratory syndrome-related coronavirus (SARS-CoV-1).[4] SARS-CoV-2 is thought to be transmissible largely by respiratory droplets or direct contact,[5] but might also be transmitted through aerosolization.[6] SARS-CoV-2 disease severity ranges from no to minimal symptoms, mildly symptomatic with cough and dyspnea, to severe respiratory distress with multi-organ failure requiring admission to an intensive care unit and emergent ventilator support.[7] Although data are evolving, the severity of illness varies with age, co-existing comorbidities, and biological sex, with older age, people with pre-existing cardiovascular disease, and males manifesting greater disease severity.[8]

A worldwide effort is in place to contain and suppress human-to-human transmission.[9] These public-health strategies aim to slow the rate of spread and reduce the burden on critical care infrastructure. However, we also need effective therapeutics. Vaccine trials are underway but potential approvals are at least a year away.[10] Development of new drugs de novo to treat SARS2-CoV-2 will likely take even longer. Thus, the most expedient therapeutic strategy to confront this pandemic will repurpose existing FDA-approved therapeutics. One potential strategy targets viral components directly, using existing antivirals and anti-infectives currently used for other diseases. Such efforts include trials of hydroxychloroquine, remdesivir, and ribavirin.[11,12] Another strategy involves targeting the human proteins, rather than viral proteins, required for SARS CoV-2 entry and replication.

Study Rationale

SARS-CoV-2 recognizes host cell membrane proteins and relies upon their enzymatic activity to infect host cells. Like SARS-CoV-1, SARS-CoV-2 has four structural proteins: E (envelop), M (membrane), N (nucleocapsid), and S (spike).[13] The S protein recognizes and binds the ACE2 receptor expressed on target cells. Binding of S protein to ACE2 facilitates attachment of the virus to the host cell. However, entry of the virus into the host cell requires catalytic cleavage of the S protein (a process termed S protein priming) by the host cell membrane protein TMPRSS2.[14] Thus, TMPRSS2 is required for viral entry and infection. This mechanism is similar to that used by SARS-CoV-1.[14] SARS-CoV-2 and SARS-CoV-1 can also use the host membrane protein cathepsin B and L (CatB/L) for S protein priming *in vitro*. However, TMPRSS2 appears to be required for SARS-CoV priming in the infected host whereas CatB/L is not.[14,15]

TMPRSS2 is expressed in the aero-digestive tract. TMPRSS2 is expressed within the nasal mucosa, respiratory sinuses, buccal mucosa, tracheal epithelia, bronchial epithelia, lung type 2 pneumocytes, and alveolar macrophages.[16–18] In addition to the

aero-digestive tract, TMPRSS2 is highly expressed on prostate, kidney, and pancreatic epithelia.[18]

Inhibition of TMPRSS2 reduces SARS-CoV-2 entry into target cells. Camostat mesylate is a small molecule inhibitor of TMPRSS2 currently approved in Japan for pancreatitis. Pharmacologic inhibition of TMPRSS2 with camostat mesylate prevented SARS-CoV-2 entry into cultured human lung cells.[14] Importantly, effective abrogation of SARS-CoV-2 entry did not require co-targeting of CatB/L, supporting the notion that targeting TMPRSS2 alone is sufficient to block SARS-CoV-2. This is consistent with prior investigations that demonstrated pharmacologic inhibition of TMPRSS2 was sufficient to eliminate lethal SARS-CoV-1 infection in murine models, whereas pharmacologic inhibition of CatB/L was not.[15] Moreover, murine models deficient in TMPRSS2 (*tmprss2* knockout mice) were more resistant to SARS-CoV-1 infection than wildtype controls, exhibited reduced viral replication within lung tissue, and profoundly reduced lung immunopathology after infection, suggesting that loss of the *tmprss2 gene* in humans may be protective against SARS-CoV-2 infection.[19] The *tmprss2* knockout mice appeared normal, indicating loss of *tmprss2* by itself is not pathogenic.[19] Camostat mesylate is currently being evaluated in a randomized, double-blind study for hospitalized SARS-CoV-2+ patients (NCT04321096) in Denmark. In an entirely separate line of investigation, a chemical library screen of small molecule inhibitors of TMPRSS2 for an entirely unrelated purpose identified the drug bromhexine.[20] Bromhexine demonstrated submicromolar inhibition of TMPRSS2 and suppressed TMPRSS2 driven metastatic progression in murine models.[20] The mechanism of bromhexine is not entirely clear, but it is sold over the counter in other countries, available both by mouth and via inhalation for respiratory disorders.

In contrast, *ACE2* is considered loss-of-function intolerant in humans.[21] In addition, SARS-CoV-mediated down-regulation of *ACE2* is thought to contribute to the severity of lung pathologies, indicating that down-regulation of *ACE2* may further harm the lungs of infected patients.[22]

For all of the aforementioned reasons, it seems that transcriptional regulation of *TMPRSS2* is the highest priority for clinical evaluation.

Transcriptional regulation of *TMPRSS*2. The transcriptional regulation of the *TMPRSS2* gene has been extensively characterized, most rigorously within the prostate. The *TMPRSS2* gene is located on chromosome 21 and is under the control of the androgen receptor (AR). Binding of androgens (e.g. testosterone or dihydrotestosterone) to the AR results in homodimerization and translocation of the AR to the nucleus, where it binds to its cognate androgen response element in the regulatory regions of its target genes, and thereby regulates gene expression.[23,24] It is firmly established that suppression of AR transcriptional activity through reduction in circulating androgens or direct antagonism of AR-androgen binding using AR competitive antagonists reduces expression and protein levels of TMPRSS2 within the prostate, as well as prostate cancers.[25] Remarkably, expression of TMPRSS2 also appears to be hormonally regulated within the lung and bronchial cells. Notably, the AR is expressed in type II

pneumocytes and the bronchial epithelium.[26] Androgens enrich AR binding at the TMPRSS2 enhancer and upregulate expression of TMPRSS2 in human lung cells, in a fashion similarly to that found in the prostate.[26]

In addition, our query of publicly available gene expression databases demonstrated that TMPRSS2 appears to have a high variability of expression amongst individuals.[27] To identify the most promising therapeutic opportunities, we performed a literature-wide screen of RNA-seq datasets in the NCBI Sequence Read Archive (SRA) that incorporated keywords relating to drug treatments. We identified differentially expressed genes from 3,089 distinct case-control comparisons featuring a drug treatment. Notably, anti-androgenic compounds and estrogens were among the strongest and most consistent down-regulators of *TMPRSS2* expression, while androgens consistently led to up-regulated *TMPRSS2* gene expression.[27] In other words, these studies suggest that the AR *induces* TMPRSS2 expression, whereas ER transcriptional activity is associated with *suppression* of TMPRSS2 expression. However, there was an initial *surge* in TMPRSS2 expression after estrogen treatments, followed by a gradual decrease in TMPRSS2 expression. Moreover, the highly variable expression pattern of TMPRSS2 suggests a provocative and plausible, although unproven, explanation for the wide range in disease severity for patients infected by SARS-Cov-2, as well as the higher rate of severe infections among males and the reduced rate and severity of infection in pre-pubertal children. A link between TMPRSS2 expression and susceptibility to viral infection was discovered by a recent genome wide association study that identified genetic variants with higher TMPRSS2 expression also had more severe H1N1 and H7N9 influenza.[28] Notably, H1N1 and H7N9 influenza, but not all influenza subtypes, require TMPRSS2 for priming.[29,30]

Identification of drugs that suppress TMPRSS2 expression. Based on the aforementioned data that TMPRSS2 is hormonally regulated in the lung by the AR and estrogen receptor (ER), it is hypothesized that drugs that interfere with AR driven transcription or enhance ER driven transcription will reduce TMPRSS2 expression. An informatics analysis of publicly available gene expression data to identify drugs that affect TMPRSS2 expression levels identified several existing FDA approved drugs.[27] Unsurprisingly, these included antagonists of the AR an agonists of the ER. A possible protective effect from estrogenic agonism in the context of another coronavirus was suggested from murine models whereby female mice exhibited reduced susceptibility and mortality to SARS-CoV-1 infection as compared to males. Furthermore, anti-estrogen treatments of the female mice increased their susceptibility and mortality to SARS-CoV-1.[31]

Thus, either suppression of AR or activation of ER may reduce expression of TMPRSS2. We chose to focus on suppression of AR, rather than activation of ER, because we are concerned that a potential initial estrogen-induced surge in TMPRSS2 expression could acutely worsen viral infections. Fortuitously, a wealth of FDA approved drugs block AR signaling. These include the GnRH analogs that reduce pituitary release of luteinizing hormone (LH) and follicle-stimulating hormone (FSH), thereby potently suppressing testosterone production, and anti-androgens that interfere with binding of

androgens to the AR. These drugs are predominantly used in the treatment of prostate cancer, have well known and generally tolerable side effect profiles, and exhibit reversibility of their biologic effects. As such, we contend that androgen suppression will reduce TMPRSS2 expression in the target cells of SARS-CoV-2 and reduce the severity of illness.

Hypothesis and Overarching Objective

- ***Our overarching objective*** *is to investigate if temporary androgen suppression that suppresses expression of TMPRSS2 will reduce severity of COVID-19 disease due to SARS-CoV-2 infection.*

- ***Our specific hypothesis*** *is that the GnRH antagonist degarelix reduces the mortality, the need for hospitalization and/or the need for intubation/ECMO at Day 15.*

Relevance to the VA and VA Population

The COVID-19 pandemic is affecting a wide swath of the US population. Mortality from COVID-19 is higher in older, male patients. Given the demographics of the Veteran population, there is a critical need to develop therapies that can reduce the severity, mortality and duration of hospitalization for our Veteran population.

Selection and Description of Study Drug

Degarelix is an FDA-approved drug for prostate cancer. Of all the FDA-approved agents that inhibit AR signaling, degarelix exhibits the most rapid and robust effects. One loading dose of 240 mg subcutaneously (SQ) serves as a 30-day depot. As a luteinizing hormone releasing hormone (LHRH) antagonist, degarelix acts at the level the pituitary to rapidly reduce LH secretion, thereby decreasing testosterone production within the testes. A rapid reduction in circulating total testosterone to castrate levels is achieved in the majority of patients within 24 hours and virtually all patients in 2-3 days (see attached degarelix package insert). The testosterone level recovers after the 30-day depot is cleared. Degarelix was selected among other GnRH analogs due to its rapid effect on circulating testosterone, safety profile, and availability. For example, LHRH agonists (e.g. leuprolide) achieve castrate levels of testosterone over 2-3 weeks; the potent androgen receptor antagonists (e.g. enzalutamide and apalutamide) achieve steady-state in the serum over approximately 4 weeks. Clearly, these time frames for LHRH agonists and AR antagonists are not suited to testing in acutely ill patients with COVID-19.

Importantly, although LHRH analogs, including LHRH agonists and antagonists are well-tolerated in patients with advanced prostate cancer who represent an older population (median age ~66-68), there may be increased risk of cardiovascular complications. Risk of cardiovascular complication is associated with duration of exposure. For example, in a randomized controlled trial involving degarelix for patients with pre-existing cardiovascular disease with a median age of 72, ischemic cardiovascular events occurred in 3.4% of patients over 12 months of continuous therapy (DOI:

10.1200/jco.2015.33.7_suppl.151 Journal of Clinical Oncology 33, no. 7_suppl (March 01, 2015) 151-151). In a single dose study such as the current study, cardiovascular complications are expected to occur at an even lower frequency. Given that the patients to be enrolled in this study are acutely ill, daily monitoring and correction of electrolytes and blood counts will be performed to mitigate risk.

2. STUDY OBJECTIVES AND ENDPOINTS

Objectives:

The primary objective of this study is to determine if temporary androgen suppression induced by degarelix improves the clinical outcomes of Veterans who are hospitalized to an acute care ward due to COVID-19 as defined by a reduction in mortality, ongoing need for hospitalization, or requirement for mechanical ventilation/extracorporeal membrane oxygenation (ECMO).

The secondary objectives of this study are to determine if temporary androgen suppression induced by degarelix reduces time to the clinical improvement, inpatient mortality, length of hospitalization, duration of intubation for mechanical ventilation, time to achieve a normal temperature, or the maximum severity of COVID-19 illness.

Endpoints:

Primary Endpoint:

Determine if degarelix + best supportive care (BSC) as compared to placebo + BSC reduces the composite endpoint of mortality, need for ongoing hospitalization, or requirement for mechanical ventilation/extracorporeal membrane oxygenation (ECMO) at Day 15 after randomization.

Secondary Endpoints:

- Determine if degarelix + best supportive care (BSC) as compared to placebo + BSC reduces the composite endpoint of mortality, ongoing need for hospitalization, or requirement for mechanical ventilation/extracorporeal membrane oxygenation (ECMO) at Day 30 after randomization.
- Determine if degarelix + BSC as compared to placebo + BSC reduces time to the clinical improvement as defined by a decline of 2 categories or more from the baseline on the modified 7-category ordinal scale of clinical status of hospitalized influenza patients (influenza scale, see Appendix A)[1], or hospital discharge, whichever comes first.
- Determine if degarelix + BSC as compared to placebo + BSC reduces inpatient mortality.
- Determine if degarelix + BSC as compared to placebo + BSC shortens the duration of hospitalization.

- Determine if degarelix + BSC as compared to placebo + BSC shortens the duration of intubation for mechanical ventilation.
- Determine if degarelix + BSC as compared to placebo + BSC reduces the time to normalization of temperature ($T < 37.5$℃ for ≥ 48 hours).
- Determine if degarelix + BSC as compared to placebo + BSC reduces the maximum severity of COVID-19 illness based on the influenza severity scale (see Appendix A).

Exploratory Endpoints

- Determine if age, use of angiotensin converting enzyme inhibitors, the duration of pre-hospitalization symptoms, or the presence or absence of hypertension, cardiovascular disease, asthma, diabetes mellitus, or COPD are predictive of clinical outcome defined by the primary endpoint or any secondary endpoint.

- Determine if baseline, Day 8 or Day 15 values for D-dimer, IL-6, LDH, ferritin, total WBC, absolute neutrophil count (ANC), absolute lymphocyte count (ALC), or testosterone are predictive of clinical outcome defined by the primary endpoint or any secondary endpoint.

- Determine if degarelix + BSC as compared to placebo + BSC reduces nasopharyngeal (NP) epithelial or peripheral blood mononuclear cell (PBMC) TMPRSS2 expression, NP and plasma viral load, or serum cytokine (TNFα, IL-1β, IL-6) levels.

- Determine whether germline genomic factors are predictive of clinical outcome defined by the primary endpoint or any secondary endpoint. This analysis is optional and is contingent on obtaining adequate number of samples.

- To determine the temporal onset of anti-viral antibodies.

Definition of Need for Ongoing Hospitalization (i.e. discharged): Patients who no longer require hospitalization on an acute bed ward for COVID-19 or its complications will be considered discharged (i.e. do not need ongoing hospitalization). For example, if a patient does not require ongoing hospitalization on an acute care ward due to COVID-19 but has remained on a non-acute inpatient unit (e.g. physical rehabilitation unit), the patient will be considered discharged for the purpose of the endpoints of this study. However, if the patient has a major complication of the hospitalization for COVID-19 (e.g. stroke) and requires ongoing hospitalization for this complication in an acute care bed, then this the patient will be considered as hospitalized for the purpose of endpoint definition even if the COVID-19 itself is deemed to have been adequately treated (i.e. no ongoing signs and symptoms that are directly due to COVID-19).

3. STUDY POPULATION

Rationale for Patient Selection

We have selected to study male patients, ≥18 - ≤ 85 years old (patients > 85 can be enrolled if there is no history of COPD, asthma, cardiovascular disease, hypertension, diabetes mellitus or active malignancy), who manifest symptoms and a disease severity that warrant hospitalization for supportive care. In this patient population, the virus itself still drives the severity of the disease, whereas a hyperinflammatory response is thought to mediate from ARDS, SIRS, and respiratory failure that underlie the severity of COVID-19 in critically ill, intubated ICU patients. (A separate study has been designed for patients with mild severity COVID-19). Female patients will not be included in this study because female androgen suppression *reduces* endogenous estrogenic activity and thereby induces TMPRSS2 expression, which in turn could exacerbate COVID-19. Studies of pre- and post-menopausal women are planned in other studies.

Although it is recognized that older patients are more susceptible to the most severe complications of COVID-19, younger patients (e.g. < 40 years old) were not excluded because such patients have a clinically significant risk of adverse outcomes including the requirement for mechanical ventilation and death. In addition, long-term complications of degarelix on fertility are not expected from the single dose of degarelix so that in balance and based on a risk-benefit profile of the degerilex, it was decided to include younger patients.

Recruitment

Patient Identification

Establishment and coordination of site-specific active surveillance mechanisms for patient identification are the responsibility of the LSI and local site study team members. The general recruitment strategy for all sites requires the early identification, screening, and contact of patients seen by the medical center's primary care and sub-specialty medical clinics, other clinics, and inpatient units responsible for the evaluation and treatment of patients with likely or confirmed diagnoses of COVID-19.

Male Veterans who are admitted or transferred from an outpatient clinic or the emergency department to an acute care bed will be identified by any of the following clinical services:

1. the admitting team,
2. the emergency department staff caring for a patient who is being admitted to acute care, non-ICU bed, or
3. the outpatient care team caring for a patient who is being admitted to acute care, non-ICU bed.

IRB approved flyers that indicate the contact information, including phone numbers, pager numbers, and email of study staff will be posted on the wards and in the emergency department and outpatient clinics.

The PI, one of the sub-investigators, or a study coordinator will be contacted about potential patients. Potential patients will then be approached by study staff about the study to initiate the informed consenting process.

Eligibility Criteria

Inclusion Criteria

1. Male Veterans admitted to a VA hospital.
2. Age ≥ 18.
3. Hospitalized on an acute care ward with a diagnosis of COVID-19 contributing to hospitalization.
4. Positive RT-PCR assay for SARS-CoV-2 on a nasopharyngeal swab sample.
5. Severity of COVID-19 illness of level 3, 4 or 5 on the influenza severity scale (see Appendix A) at the time of randomization.
6. The subject (or legally acceptable representative if applicable) must provide written informed consent for the trial.

Exclusion Criteria

2. History of severe hypersensitivity to degarelix or any component of its formulation.
3. History of congenital long QT syndrome or known history of prolonged QT interval OR Fridericia correction formula (QTcF) > 500 msec on electrocardiogram on electrocardiogram performed during screening .
4. Planned discharge within 24 hours of treatment initiation.
5. Subject is planning to conceive or father children within the projected duration of the study, starting with the screening visit through 120 days after the last dose of study treatment.
6. Ongoing usage of a Class IA or Class III antiarrhythmic agent (see Appendix D). At least 5 half-lives must elapse since any prior use of a Class IA or III antiarrhythmic agent prior to administration of study drug.
7. Baseline electrolyte abnormalities of Grade 3 or higher (based on CTCAE v5.0 criteria). Patients may be included if baseline electrolyte abnormalities are corrected to Grade 2 or lower prior to study drug administration.
8. Enrollment in another investigational study within 30 days of Day 1.
9. Myocardial infarction in the past 6 months, severe or unstable angina, or New York Heart Association (NYHA) Class III or IV heart disease.
10. Known psychiatric or substance abuse disorder that would interfere with the requirements of the trial.
11. Child-Pugh Class C liver disease.
12. Use of any of the following hormonal agents:
    a. Androgen receptor antagonists or agonists within 4 weeks of study enrollment,
    b. Ketoconazole or abiraterone acetate within 2 weeks of study enrollment,
    c. Estrogens or progestins within 2 weeks of study enrollment,

d. Herbal products that contain hormonally active agents within 2 weeks of study enrollment.
e. Any prior use of an LHRH analogue unless a serum total testosterone measured within 30 days of study enrollment is ≥ 150 ng/dL.
f. Other hormonal agents listed in Appendix B within one day of study enrollment.

13. Unwilling or unable to comply with the study protocol.
14. Any condition, which in the opinion of the investigator, would preclude participation in the trial.

NOTE: We will not be excluding patients based upon renal or hepatic dysfunction, except for patients with Child-Pugh Class C liver disease; according to the degarelix package insert, degarelix has been safely administered to patients with Child-Pugh class A and B liver disease. Moreover, grade 3 and higher liver enzyme abnormalities occur in <1% of patients treated with degarelix (degarelix package insert). In clinical practice, degarelix is used at full dose in the setting of creatinine clearance <50 ml/min and severe liver disease without excess toxicity. In addition, the study population will receive one and only one dose of degarelix, which will result in temporary androgen suppression. Most of the side effects of degarelix are attributable to the chronic suppression of serum testosterone as is typically done for prostate cancer patients, so it is predicted that study patients will likely have fewer side effects from degarelix than prostate cancer patients.

4. STUDY ENROLLMENT PROCEDURES

Screening

Patients will undergo a screening evaluation during a 72-hour window. The screening period begins when the patient or his legally authorized representative (LAR) has signed the informed consent form.

Informed Consent

The Investigator must obtain documented consent from each potential study patient prior to conducting any research activities. Patients will be provided complete informed consent that will be conducted by site PIs, designated sub-investigators, and/or study staff. Subjects will have the option of having family and/or anyone else they deem appropriate present during the informed consent process. The PI and/or co-investigators will verbally discuss the study with the patient and/or the patient's legally authorized representative (LAR). The patient or LAR will be given the informed consent form (ICF) and encouraged to carefully read and review the document. After reading the informed consent form, the patient or LAR will be encouraged to ask any and all questions. The investigators will in turn pose questions that will evaluate their understanding of the nature and risks of the study. The copy of the ICF can be an electronic iMED, DocuSign or hard copy.

Although the consenting process can be done in the patient's room, due to the nature of the risks of exposure to a COVID-19 patient, direct communication with the patient may not be feasible or safe. Accordingly, the investigator or delegated research staff will obtain the patient's phone number (e.g. bedside phone or cell phone), and arrange a three-way call or video conference with the patient (or LAR), an impartial witness, and if desired and feasible, additional patients requested by the patient or LAR (e.g. next of kin). To ensure that patients are approached in a consistent fashion, a standard process should be used when contacting the patient remotely that will accomplish the following:

- Identification of who is on the call.
- Review of the ICF with the patient by the investigator (or their designee) and response to any questions the patient may have,
- Confirmation by the witness that the patient's questions have been answered,
- Confirmation by the investigator that the patient is willing to participate in the trial and sign the informed consent document while the witness is listening on the phone,
- Verbal confirmation by the patient that they would like to participate in the trial and that they have signed and dated the informed consent document that is in their possession.

If the signed informed consent document will not be able to be collected from the patient's location and included in the study records, the following two options are acceptable to provide documentation that the patient signed the informed consent document:

- **Option #1:** Attestations by the witness who participated in the call and by the investigator that the patient confirmed that they agreed to participate in the study and signed the informed consent,

Please note: It is recommended that the documented verbal confirmation include information on the version of the IRB-approved informed consent document that was used, such as IRB-approved informed consent date and version 1.0, or other type of designation such as IRB-approval date.

- **Option #2**: A photograph of the informed consent document with attestation by the person entering the photograph into the study record that states how that photograph was obtained and that it is a photograph of the ICF signed by the patient.

A copy of the informed consent document signed by the investigator and witness should be placed in the patient's trial source documents, with a notation by the investigator of how the consent was obtained, e.g. telephone. The trial record at the investigational site should document how it was confirmed that the patient signed the ICF (i.e., either using attestation by the witness and investigator or the photograph of the signed consent). The note should include a statement of why the ICF signed by the patient was not retained, e.g., due to contamination of the document by infectious material.

If the patient is unable to provide informed consent and there is a LAR, investigators should obtain consent from the patient's LAR.

The ICF, any subsequent revised written ICF and any written information provided to the patient must receive the IRB approval in advance of use. The patient or his/her legally acceptable representative should be informed in a timely manner if new information becomes available that may be relevant to the patient's willingness to continue participation in the trial. The communication of this information will be provided and documented via a revised consent form or addendum to the original consent form that captures the patient's dated signature or by the patient's legally acceptable representative's dated signature. Specifics about a trial and the trial population will be added to the consent form template at the protocol level.

The informed consent will adhere to IRB requirements, applicable laws and regulations and Sponsor requirements.

Subjects will be informed that they may refuse participation in or withdraw from this study at any time without prejudice or any negative effect on subsequent care. In addition, subjects will be informed that their health care provider may be one of the investigators on this study and a conflict of interest may therefore exist in that the investigator may be interested in both the patient's welfare and in the conduct of the study.

LAR and Surrogate Consent

The study will utilitze the assent process for patients who are unable to give informed consent. The PI and/or co-investigators will verbally discuss the study with the patient and/or the patient's legally authorized representative (LAR)/surrogate. In the event that an LAR is needed, the study team will educate the LAR/surrogate on the consent process. It will be explained that the LAR/surrogate is being asked to act on behalf of the patient because he lacks capacity and is unable to consent on his own behalf in this situation. It will be emphasized to the LAR/surrogate that he/she should determine what he/she believes is best for the patient. It will also be explained that even though the LAR/surrogate consents to the patient's being in the study, if the patient expresses that he does not want to participate in the study, his dissent will be honored and respected, even if the LAR/surrogate wants him to participate in the study. The patient or LAR/surrogate will be given the informed consent form (ICF) and encouraged to carefully read and review the document. After reading the ICF, the patient or his LAR/surrogate will be encouraged to ask any and all questions. The investigators will in turn pose questions that will evaluate the LAR/surrogate's understanding of the nature and risks of the study. Before obtaining consent from the LAR/surrogate, study team will ask the patient if he wishes to participate in the study. If the patient agrees to participate in the study, then the informed consent process will be completed by the LAR/surrogate. If the patient does not want to participate in the study, then his dissent will be honored and respected.

However, there are situations where assent cannot be obtained due to either the patient's having disorders involving disturbances of cognitive or intellectual functioning (for instance, in mentally challenged patients, or in patients with dementia or patients in confused states), rendering them incapable of understanding the disclosed information at that time.

For patients who are diagnosed with a cognitive disorder (e.g. dementia, Alzheimer's Disease, bi-polar, schizophrenia or mentally challenged), or in a state of delirium due to superimposed complications of COVID, every effort will be made to obtain assent. However, due to Legally Authorized Representatives (LARs)/surrogate not being allowed in the COVID ward, these individuals may not have an accurate perception of the patient's mental and physical capacity. In this situation, the study will solicit the help of an "honest broker" (a provider who is not part of the study team) within the unit to help the LAR/surrogate understand the patient's capacity to participate in the study.

If the investigator(s) conclude(s) that assent may not be possible, an honest broker will be asked to witness the process. The honest broker and the investigator(s) will provide input on the patient's capacity to participate in the study to the LAR/surrogate so he/she can make an informed decision as to whether the surrogate/LAR should provide consent for the patient to participate in the research study. If the LAR/surrogate provides consent for the patient to participate, then we study will only obtain consent from the LAR/surrogate if assent is not possible.

Randomization Procedure

Patients will be randomized after the investigator has verified that all eligibility criteria have been met and the patient has been enrolled. Patients will be randomized 2:1 to BCS + degarelix, or BCS + placebo using a block randomization scheme. Random block sizes of 3 and 6 will be used to reduce the chances of guessing future allocations. Randomization will be performed using the Interactive Web Response System (IWRS), a computerized system that will allow authorized study personnel to randomize patients and obtain study treatment assignments. Following randomization, the site coordinator will obtain a randomization certificate containing the assigned randomization number. Following randomization, the randomization certificate will be given to the site pharmacy. The site pharmacist will then log into the IWRS and use the information on the randomization certificate to obtain a treatment assignment certificate which will indicate whether the patient has been assigned to degarelix or placebo. The URL for the IWRS is:

https://vaww.abq.csp.va.gov/csp/ctsc/HITCH/main.cfm/logins/welcome

<u>All patients should commence assigned treatment as soon as possible and no later than 48 hours (2 calendar days) after randomization.</u>

Stratification Factors

Patients will be stratified according to 3 factors:

- Age: <65 vs. ≥65
- History of hypertension prior to hospitalization: yes vs. no

- Influenza severity scale: 3 vs 4/5

Study site is not a stratification factor because the participating sites will be in close communication to minimize variability in providing BSC. Other potential factors that can influence clinical outcome include COPD, asthma, and cardiovascular disease will be assessed for their relationship to clinical outcome in exploratory analyses.

Blinding

This is a randomized, double-blind study. Patient treatment assignments will remain blinded. The study biostatisticians and clinical research pharmacist may access treatment assignment information as necessary.

The investigational drug blind will be maintained using the IWRS. The drug and placebo injections will be dispensed by the research pharmacy and packaged to be made indiscernible from one another. To maintain the blind, the IWRS will ensure the investigator or designee is unaware of the treatment arm assigned to the subject. Prior to dispensing the study medications, the research pharmacist will verify the appropriate treatment arm assignment using the IWRS cross-referencing the patient's randomization number. The study drug will be delivered to an unblinded administrator of the study drug who is not part of the study team and who will not disclose information regarding study drug administration to any member of the study team, the patient or anyone else.

Unblinding Procedure

The CSPCRPCC will not provide Emergency Code Envelopes to the Research Pharmacy for HITCH. Emergency unblinding will be managed through the 24-hour emergency call service (505-248-3203). This number is also listed on the patient ID cards given to each patient participating in HITCH. The system managed by the CSPCRPCC will electronically capture up-to-date study drug assignment information gathered from the HITCH IWRS.

Authorization to Break the Blind

Under unusual circumstances, chiefly related to patient safety and emergency medical necessity, unblinding may be necessary if knowledge of the study treatment assignment will influence the medical treatment of the patient. This is usually done after consultation with the Study Chair.

If emergency unblinding becomes necessary during local business hours, a treating provider should contact the LSI. The LSI should then contact the Study Chair to discuss the situation before requesting that a patient be unblinded by the CSPCRPCC. Outside of local business hours, a treating provider or LSI may contact the CSPCRPCC directly at a number that is available 24 hours per day, 7 days per week (505-248-3203) to discuss the need for unblinding and to have the unblinding performed if indicated. Efforts should be made to avoid unblinding unless medically necessary for emergency treatment decisions. Emergency unblinding procedures are further detailed in the Study Operations Manual.

Patients will be provided with a Patient Identification Card that lists local study staffs' telephone numbers, a 24-hour PCC contact number, and contacts for the Study Chairs. The research facility's name and the study drugs are also provided on the card. The Patient Identification Card will include the patient's Randomization Number, which can be used to determine the patient's treatment assignment should emergency unblinding be required for patient safety.

5. STUDY DESIGN

Study Design Overview

This is a randomized phase 2, placebo-controlled, double blind clinical trial to compare the efficacy of degarelix + BSC to placebo + BSC on improving the clinical outcomes of male Veterans who have been hospitalized due to COVID-19. Enrolled patients must have documented infection with SARS-Cov-2 based on a positive RT-PCR result performed on a nasopharyngeal swab. Patients will be centrally randomized 2:1 to the study arms. BSC consists of supplemental oxygen, antibiotics, vasopressor support, peritoneal or hemodialysis, antibiotics, intravenous fluids, etc. The primary endpoint is to determine if degarelix + best supportive care (BSC) as compared to placebo + BSC reduces the composite endpoint of mortality, ongoing need for hospitalization, or requirement for mechanical ventilation/extracorporeal membrane oxygenation (ECMO) at Day 15 after randomization.

Study Schema

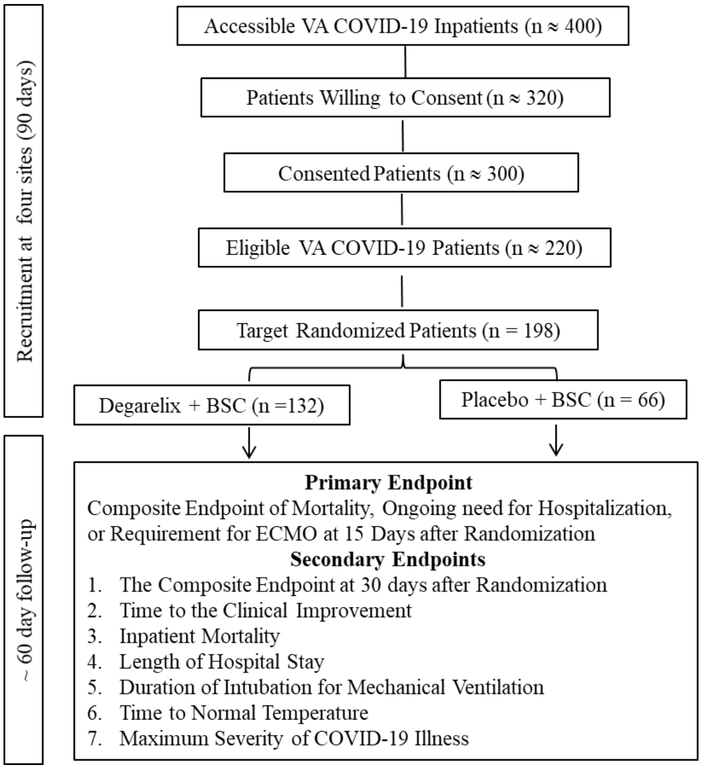

Sample Size

A total of 198 patients will be enrolled with 2:1 ratio for degarelix + BSC versus placebo + BSC (n = 132 for degarelix + BSC and n= 66 for placebo + BSC).

Study Duration

- Study enrollment = estimated 90 days (i.e. first patient in [FPI] → last patient in [LPI] = 90 days).
- Assessment of response and additional follow-up for primary endpoint = date of LPI + estimated -60 days.
- Completion of data analysis = date of LPI + estimated 60 days.
- Total study duration = estimated 210 days.

Feasibility of Accrual

The planned total accrual is 198 patients, including a 5% drop out rate. As of April 7, 2020, the sites have the following number of confirmed COVID-19 patients:

- Brooklyn: 83
- GLA: 22. There are an addition 11 patients whose COVID status is pending.
- Manhattan: 51
- Puget Sound: 9

The number of hospitalizations for confirmed COVID-19 is increasing. For example,

the Brooklyn VA had 54 patients as of 4/02/2020. Given the anticipated increase in number of hospitalized patients, we expect to reach our accrual goal within the planned 90-day period of enrollment.

Duration of Patient Follow-up

Patients will be followed until death or 60 days after randomization, whichever comes first. If a patient dies or refuses to continue participation, he will be terminated. A termination form will be used to record the termination information for these patients. If a patient remains hospitalized beyond Day 30, the patient will continue to be followed for clinical endpoints, but no additional research labs will be obtained.

6. STUDY TREATMENT

Degarelix

Degarelix (as the acetate) is formulated as a sterile lyophilized powder for injection. Degarelix forms a depot upon subcutaneous administration, from which it is released to the circulation. The long half-life after subcutaneous administration is a consequence of a very slow release of degarelix from the depot formed at the injection site(s). The 240 mg dosage is achieved by the administration of two 120 mg SQ doses in the deep abdominal subcutaneous tissue.

The 240 mg dosage was chosen because it results in rapid suppression of serum testosterone levels. This rapid testosterone suppression is predicted to result in downregulation of TMPRSS2 expression, which can inhibit further viral infection and propagation and thereby reduce the clinical severity of COVID-19. One and only one dose was selected because the suppressive effect on testosterone will endure for at least four weeks, which will allow for the potential disease remitting effects to be observed amongst hospitalized COVID-19 patients, who have a median time to clinical improvement of 15 days (Remdesivir study).

The normal target population for this drug is men. In this study, only men will receive the drug. The indication in the package insert is patients with advanced prostate cancer. The study population does not target men with prostate cancer. Men with prostate cancer get one loading dose and then are continued indefinitely on a lower dose of the medication. The study population will receive one and only one dose of degarelix (loading dose), which is expected to result in temporary androgen suppression. Most of the side effects of degarelix are attributable to the chronic suppression of serum testosterone as is typically done for prostate cancer patients, so it is predicted that study patients will likely have fewer side effects from degarelix than prostate cancer patients.

Degarelix is well tolerated. Most side effects are attributable to suppression of serum testosterone levels, such as hot flashes and erectile dysfunction. Degarelix and other LHRH analogues have been associated with an increase risk of cardiovascular complications, although these effects are observed with chronic administration of degarelix; the increased risk appears to be restricted to patients with pre-existing

cardiovascular disease. The current study involves one and only one dose of degarelix, which will temporarily suppress serum testosterone. In addition,

A prolonged QTcF interval may occur in patients on LHRH analogues, but is more frequent in patients receiving LHRH agonists as opposed to LHRH antagonists like degarelix. In a randomized controlled trial, the incidence of prolongation in the QTcF to >500 msec was <1% over 12 months of continuous therapy with degarelix; ventricular arrhythmias including torsades de pointes did not occur in any patient over 12 months. Thus, although cardiotoxicity represents a potential complication of degarelix, we expect that the potential benefit of degarelix is greater than the cardiovascular risk especially given the one-time nature of the degarelix administration in the current study. Nonetheless, we have included ongoing assessments of adverse events of special interest, including arrhythmias and thromboembolic events (please see Adverse Events and Serious Adverse Events, Safety Monitoring and Statistical Considerations sections for detailed discussion). In a phase 2 study involving two months of degarelix (one loading dose of 240 mg followed by one maintenance dose of 80 mg), no grade 3 toxicities were observed amongst 45 patients who were monitored until gonadal recovery (UCLA abstract).

Moreover, an 80 patient trial was conducted for the explicit purpose of determining if single dose degarelix at supratherapeutic concentrations has an intrinsic effect on cardiac repolarisation and the QT interval (Olsson et al. Clin Drug Investig . 2017 Sep;37(9):873-879. DOI: 10.1007/s40261-017-0547-7). The conclusion was that degarelix by itself does not have any effect on the QT interval and cardiac repolarization even at supratherapeutic concentrations.

Systemic allergic reactions from degarelix are rare. In a randomized controlled trial that included over 400 patients chronically treated with degarelix, no systemic allergic reaction including anaphylaxis was observed (BJU Int. 2008; 102(11):1531–1538)

Best Supportive Care

Best supportive care will include all treatments that would be applied irrespective of patient enrollment and includes but is not limited to supplemental oxygen, antibiotics, vasopressor support, peritoneal or hemodialysis, antibiotics, intravenous fluids. The treatment landscape for COVID-19 is rapidly evolving. Accordingly, the clinical trial will allow for updates to the allowed treatments. For example, emergency authorization of remdesivir was recently granted, so remdesivir usage is allowed prior to or during study enrollment. In addition, use of convalescent plasma is not uncommon, is available through an expanded access program and will be allowed prior to or during study enrollment. New treatments will have a drug utilization report assessment prior to use to identify overlapping toxicities prior to use of a new treatment.

Off-label use of other agents or interventions will be allowed. For example, anti-coagulation is recommended by various societies, including the American Society of

Hematology. Off-label use of IL-6 antibodies will also be allowed on study; however, formal enrollment to another investigational study involving IL-6 antibodies is prohibited.

The development of novel treatments and interventions for COVID-19 will be monitored by lead site investigators (Drs. Rettig and Nickols) as well as other study team members. Appropriate changes to allowed treatments and interventions will be instituted accordingly.

Description of Treatment Regimens

Patients will be randomly assigned to receive a one-time dose of degarelix 240 mg SQ or matching placebo on day 1. The matching placebo will contain equal volume 0.9% saline.

No dose modifications are allowed for degarelix***.***

Prescribing and Dispensing Study Products

Study medication (Degarelix (Firmagon) 240 mg or placebo) will be dispensed by the local research pharmacist at each site after careful confirmation of treatment assignment.

In addition to best supportive care, each patient will receive a one-time dose of study medications supplied as 2 – 3ml syringes for deep SQ administration on either side of the anterior abdomen in the general periumbilical area.

Treatment 1 - Active Degarelix (2 – prefilled syringes containing 3 ml of reconstituted Degarelix concentrated to 40mg/ml)

Treatment 2 - No active, only placebo (2 – prefilled syringes containing 3 ml of 0.9% saline)

The Local Research Pharmacist will dispense study medications according to the treatment assignment in IWRS. Upon receipt of an approved study medication order, the Local Research Pharmacist will use the IWRS to verify the correct treatment assignment prior to dispensing the study medication to the unblinded administrator. Each study medication order must be logged.

Sixty Minute Rule: If the study medication (degarlix or placebo) cannot be administered within 60 minutes from the time of reconstitution/preparation, the medication cannot be administered. Even though the placebo is not affected, procedures should be in place, that maintain the blind. In such cases, the Local Research Pharmacist will need to document the reasons for delay and replacement.

Method of Administration

Degarelix is administered as a subcutaneous injection in the abdominal region. Injections should be given in areas of the abdomen that will not be exposed to pressure, e.g., not close to waistband or belt nor close to the ribs.

Degarelix should *not* be administered IV.

The degarelix or placebo will be administered deep SQ in the abdominal area by pinching skin and elevating SQ tissue by a study-approved clinician;

- Insert the needle at a 45-degree angle.
- Gently pull plunger back to check for aspiration
    - (if blood is aspirated into syringe, do not inject; discard and reconstitute a new dose)
- Slowly inject over 30 seconds, remove needle and then release skin.
- Repeat this procedure for both doses.

Each dose should be administered on either side of the anterior abdomen in the general periumbilical area.

Source and Handling of Study Drug

The local VA pharmacy will be supplying the degarelix (Study Drug) and matched SQ placebo. The placebo will consist of normal saline. The local VA Pharmacy will purchase a supply of degarlix for exclusive use in the study. The Pharmacy will be reimbursed for the cost of degarlix from by the study. The placebo (normal saline) will be supplied by the pharmacy.

Degarelix for injection (Firmagon ®) 240 mg for subcutaneous use can be obtained from VA National Prime Vendor. The site pharmacy must ensure that adequate supplies of degarlix are available for dispensing and/or administering to study patients and should account for lead times in drug procurement. A minimum amount of degarelix will be kept on hand at all times (enough drug to treat 20 patients with degarelix for the maximum duration of treatment = degarelix x 1 dose administered within 24 hours of randomization).

Degarelix is a commercially available product with expiration dating assigned by the manufacturer. The research pharmacy will monitor the dating of all study drug to ensure that the drug will not expire through the anticipated administration and use by the patient.

Study drug must be stored in a secure, limited-access location under the storage conditions specified on the label. Site pharmacies are responsible for providing secure storage for study drugs in the Pharmacy or elsewhere as directed by federal and local laws, regulations, policies, and procedures. Investigational drugs must be stored separately from non-study drug inventory to avoid co-mingling of investigational and clinical drug products.

Receipt and dispensing of trial medication must be recorded by an authorized person at the trial site. Drug will be stored per manufacturers' recommendations in the VA Clinical Research Pharmacy. The VA Clinical Research Pharmacy will maintain an inventory of the study drug, including the lot number, expiration date, amount shipped, date of shipment, dates/amounts dispensed, and remaining amounts of drug. Site pharmacists should consult VHA Handbook 1108.04 "Investigational Drugs and supplies.

Degarelix requires special hazardous agent handling. (NIOSH 2016 [group 1]). Use appropriate precautions for receiving, handling, administration, and disposal. Gloves (single) should be worn during receiving, unpacking, and placing in storage.

Degarelix supplies may not be used for any purpose other than that stated in the protocol. The investigational drug will be dispensed and administered only for the purposes of HITCH. The LSI is responsible for assuring that the HITCH investigational drug is administered only to HITCH study patients by qualified and approved study personnel. The LSI is ultimately responsible for all study drug at their clinical site.

If any dispensing errors or discrepancies are discovered, the sponsor must be notified immediately. The investigator or designee must record the current inventory of all study medications (degarelix 240 mg) on a sponsor-approved drug accountability log. The following information will be recorded at a minimum: protocol number and title, name of investigator, site identifier and number, description of study medications, expiry date and amount dispensed including initials, seal, or signature of the person dispensing the drug, and the date and amount returned to the pharmacy by the site (if applicable), including the initials, seal, or signature of the person receiving the study medications. The log should include all required information as a separate entry for each subject to whom study medications is dispensed. Prior to site closure or at appropriate intervals, a representative from the sponsor or its designee will perform study medications accountability and reconciliation before study medications are returned or locally destroyed. The investigator or designee will retain a copy of the documentation regarding study medications accountability, return, and/or destruction, and originals will be sent to the sponsor or designee.

Packaging and Labeling Information

Drugs or matched placebos will be supplied to the research staff in a manner that does not identify the active versus placebo drug. The research staff will remain blinded to the treatment group assignment.

Storage and Handling Requirements

Clinical supplies must be stored in a secure, limited-access location under the storage conditions specified on the label. Site pharmacies are responsible for providing secure storage for study drugs in the Pharmacy or elsewhere as directed by federal and local laws, regulations, policies, and procedures. Investigational drugs must be stored separately from non-study drug inventory to avoid co-mingling of investigational and

clinical drug products. *Site pharmacists should consult VHA Handbook 1108.04 "Investigational Drugs and supplies"*

Clinical supplies may not be used for any purpose other than that stated in the protocol. The investigational drug will be dispensed and administered only for the purposes of HITCH. The LSI is responsible for assuring that the HITCH investigational drug is administered only to HITCH study patients by qualified and approved study personnel. The LSI is ultimately responsible for all study drug at their clinical site. Receipt and dispensing of trial medication must be recorded by an authorized person at the trial site. Drug will be stored per manufacturers' recommendations in the VA Clinical Research Pharmacy. The VA Clinical Research Pharmacy will maintain an inventory of the study drug, including the lot number, amount shipped, date of shipment, dates/amounts dispensed, and remaining amounts of drug. A minimum amount of drug will be kept on hand at all times (enough drug to treat 20 patients with degarelix for the maximum duration of treatment = degarelix x 1 dose administered within 24 hours of randomization).

The study drugs are commercially available products with expiration dating assigned by the manufacturer. The research pharmacy will monitor the dating of all study drug to ensure that the drug will not expire through the anticipated administration and use by the patient.

Hazardous Drugs Handling Considerations

Degarelix requires special hazardous agent handling. (NIOSH 2016 [group 1]). Use appropriate precautions for receiving, handling, administration, and disposal. Gloves (single) should be worn during receiving, unpacking, and placing in storage.

Adverse Reactions

Degarelix (see Appendix F)

- >10%:
  - Central nervous system: Fatigue (3% to ≥10%)
  - Endocrine & metabolic: Hot flash (26%), increased gamma-glutamyl transferase (≥10%), weight loss (≥10%), weight gain (9% to ≥10%)
  - Hepatic: Increased serum transaminases (47%)
  - Local: Injection site reactions (35%, grade 3: ≤2%; pain at injection site [28%], erythema/bruising at injection site [17%], swelling at injection site [6%], induration at injection site [4%], injection site nodule [3%], injection site infection [including abscess, 1%])
  - Miscellaneous: Fever (1% to ≥10%)
- 1% to 10%:
  - Cardiovascular: Hypertension (6%)
  - Central nervous system: Chills (5%), dizziness (1% to 5%), headache (1% to 5%), insomnia (1% to 5%)
  - Dermatologic: Diaphoresis
  - Endocrine & metabolic: Hypercholesterolemia (3%), gynecomastia
  - Gastrointestinal: Constipation (5%), nausea (1% to 5%), diarrhea

Genitourinary: Urinary tract infection (5%), erectile dysfunction, testicular atrophy
Hepatic: Increased serum ALT (10%; grade 3: <1%), increased serum AST (5%; grade 3: <1%)
Immunologic: Antibody development (antidegarelix: 10%)
Neuromuscular & skeletal: Back pain (6%), arthralgia (5%), weakness (1% to 5%)
Miscellaneous: Night sweats (1% to 5%)

- <1%, postmarketing, and/or case reports.

  Bone metastases (worsening), cerebrovascular accident, depression, hypersensitivity reaction (including anaphylaxis, urticaria, and angioedema), itching at injection site, local soreness/soreness at injection site, malignant lymphoma, mental status changes, myocardial infarction, osteoarthritis, prolonged Q-T interval on ECG, squamous cell carcinoma, unstable angina pectoris.

Dose Modification

No dose modifications will be allowed. Degarelix will be administered as a one-time dose, so dose modification does not apply.

Prohibited Concurrent Treatments and Medications

- Medications listed in the exclusion criteria.
- Any medication that can serve as pro- or anti-androgen, -estrogen, or -progestin (See Appendix B). Dexamethasone was recently reported to reduce COVID-19 severity, so glucorticoids for the purpose of treating COVID-19 are permitted.
- Use of other investigational drugs for COVID-19 treatment within 28 days (or five half-lives whichever is shorter; with a minimum of 14 days from the last dose) preceding the Day 1 of treatment. Use of hydroxychloroquine or chloroquine is permitted.
- Use of hydroxychloroquine and chloroquine are prohibited
- Use of drugs known to prolong QT interval are prohibited

7. STUDY PROCEDURES

Table 1. Schedule of Events

Assessment	Screening[A]	Day 1	Day 8	Day 15	Day 22	Day 30	Day 60
Informed consent	X						
Eligibility	X						
Demographics	X						
Vital signs/Medical/interval history[B]	X	X	X	X	X	X	
Concomitant medications	X		X	X	X	X	
AEs	X	X	X	X	X	X	X
Clinical assessment of status[C]	X	X	X	X	X	X	
Serum total testosterone level[D]	X		X	X		X	
D-dimer, LDH[D]	X		X				
CBC with differential, chem panel and electrolytes, coagulation tests, cardiac blood tests, and LFTs[E]	X	Daily					
Inflammatory tests[D,K]	X	Weekly					
Research blood collection[D,F,G,H]		X	X	X		X	
NP swab[D,F,G,H]		X	X	X			
Degarelix or matched placebo		X					
EKG[I]	X						
Post Discharge Follow up[J]						X	X

[A] Screening studies should be performed within 72 hours of Day 1.
[B] Vital signs, medical history and physical exam data can be accessed from the electronic medical record so as to minimize exposure to patients. This assessment should continue so long as the patient is hospitalized.
[C] Based on modified 7-category ordinal scale of clinical status of hospitalized influenza patients (see Appendix A). This assessment should continue so long as the patient is hospitalized.
[D] All labs should be performed in the local VA laboratory. The window for labs is +/- 2 days. The collection of labs will discontinue when the participant is discharged.
[E] Chem panel = Sodium, potassium, chloride, CO2, blood urea nitrogen, creatinine. Electrolytes = Calcium, magnesium, phosphorus. LFTs = AST, ALT, albumin, total bilirubin, alkaline phosphatase, LDH. Cardiac blood tests = troponin, CPK. Coagulation tests = D-dimer, PT/INR and aPTT. Some of these labs are collected for the purposes of safety assessments and risk mitiagation and will not necessarily be collected in CRFs.
[F] Research blood collection will be performed at GLA only. Research blood should include 1 gold top and 1 Streck tube; each tube will contain 5 mL of blood. The Day 1 collection can be performed up to 72 hours prior to study drug administration. The window for research blood collection on Days 8, 15, and 30 is +/- 2 days,.

[G] NP swabs will be collected at GLA, only. The Day 1 NP swab can be obtained up to 24 hours prior to study drug administration. The window for the Day 8 and 15 NP swab is +/- 2 days. All NP swabs will be placed immediately in the supplied vial containing formalin.
[H] Collection of research blood and NP swabs will be attempted at the indicated time points, but it is understood that some of these samples may not be obtained due to logistical reasons, such as safety. Accordingly, the failure to collect the research blood or NP swabs will not be considered a protocol deviation.
[I] EKG. EKGs beyond the screening window will be performed per standard of care.
[J] Upon discharge, patients will be followed for vital status, need for re-hospitalization, and adverse events.
[K] Inflammatory blood tests = CRP, IL-6, ferritin and pro-calcitonin.

Laboratory abnormalities observed during study enrollment will be managed according to local hospital practices and good standard of care. Study teams will focus on electrolyte abnormalities that could impact risk for cardiac events.

Handling of Research Specimens

The research specimens will be transported to UCLA for analysis on the same day of collection whenever possible. If samples cannot be transported to UCLA on the same day of collection, then the samples will be stored within the GLA Medical Center Building 500 Clinical Research Center. In the Clinical Research Center, the NP swabs will be stored at room temperature, and blood specimens will be stored at -20℃.

8. ADVERSE EVENTS AND SERIOUS ADVERSE EVENTS

Overview of Adverse Event Reporting

Timely and complete reporting of safety information assists study management in identifying any untoward medical occurrence. This contributes to patient safety, regulatory compliance and improvements in study design or procedures. In addition, close attention to AEs provides valuable information about the safety and tolerability of study drugs. This study specific safety plan is designed to collect information on the safety and tolerability of the study medications; thus, reporting will be conducted in accordance with the requirements of GCP and other regulatory requirements governing clinical research in the US.

Definitions and recording requirements for this trial are based on the International Conference on Harmonisation Efficacy Guideline 2a: Pharmacovigilance: Clinical Safety Data Management: Definitions and Standards for Expedited Reporting (ICH-E2A), the Code of Federal Regulations (21 CFR 312.32) and CSP Global Standard Operation Procedure (SOP) 3.6.

In HITCH, patients will receive a regimen of FDA approved commercially available degarelix for injection (Firmagon®) 240 mg for subcutaneous use or matching placebo. Local Study Investigators, with assistance from their local SC, are responsible for collecting AE and SAE information regarding the patients at their sites.

Non-serious AEs and SAEs will be collected from the time of randomization until 60 days after the last dose of study medication. Throughout the specified duration of AE and SAE collection, data will be collected through spontaneous local SC/LSI/Co-I contact, during in-person study visits, and gathered during telephone contacts and medical record reviews when performed **(see Table of Scheduled Events)**. AEs will be collected based on a review of the electronic medical record. If a patient receives care at a non-VA facility for an AE or SAE, study staff should obtain the requisite release of information form(s) from the patient and acquire the pertinent medical records from the facility.

Adverse events that are open will require follow-up reports (if necessary) every 30 days until resolved or until the end of the trial.

Adverse events reported to study staff and deemed related to the study drug(s) and all SAEs will be recorded on an AE or SAE form (respectively) and documented in source records (e.g., the electronic VA medical record and/or the patient's study record). In this way, the site creates a permanent record that provides information on the patient's clinical course while in the study.

Adverse Events (AEs)

An AE is any untoward physical or psychological occurrence in a human participating in research or associated with the use of a drug in a human. An AE can be any unfavorable and unintended event, including an abnormal laboratory finding, symptom, or disease associated with the research or the use of a medical investigational test article. An AE does not necessarily have to have a causal relationship with the research or medical investigational test article. (See 21 CFR 312.32(a) and VHA Handbooks 1058.01 and 1200.05)

Severity of an Adverse Event

Adverse events will be graded according to CTCAE v5.0.

All adverse events that are related to the study intervention or procedures are recorded at each research assessment visit (description, severity, relationship to study intervention, date onset, date resolution).

The following categories are used to convey the severity of an adverse event:

- Grade 1 Mild; – Events require minimal or no treatment and do not interfere with the patient's daily activities.
- Grade 2 Moderate; – Events result in a low level of inconvenience or concern with the therapeutic measures. Interferes with normal daily activities to some extent.
- Grade 3 Severe; – Events interrupt a patient's usual daily activity and may require systemic drug therapy or other treatment. May severely interfere with or prevent normal daily activities
- Grade 4 Life-threatening consequences; – Events requiring urgent intervention
- Grade 5 Death related to AE.

Serious Adverse Event (SAEs)

An AE is considered an SAE if, in the view of either the investigator or sponsor, it results in any of the following outcomes:

- Death:
- A life-threatening AE;

NOTE: The term “life-threatening” in the definition of “serious” refers to an event in which the patient was at risk of death at the time of the event; it does not refer to an event which hypothetically might have caused death if it were more severe.

- Inpatient hospitalization or prolongation of existing hospitalization;
- A persistent, significant, or permanent incapacity or substantial disruption in the patient's body function/structure, physical activities and/or quality of life;
- Congenital anomaly/birth defect; or
- Important medical events that may not be immediately life-threatening, result in death, or require hospitalization may be considered serious when, based upon appropriate medical judgment, they may jeopardize the patient and may require medical or surgical intervention to prevent one of the outcomes listed in this definition. Such events include intensive treatment in an emergency room or at home for allergic bronchospasm; blood dyscrasias or convulsions that do not result in hospitalization; or development of drug dependency or drug abuse. (See 21 CFR 312.32(a) and VHA Handbooks 1058.01 and 1200.05)

A serious problem is a problem in human research or research information security that may reasonably be regarded as:

1. Presenting a genuine risk of substantive harm, to the safety, rights, or welfare of human research subjects, research personnel, or others, including their rights to privacy and confidentiality of identifiable private information; or
2. Substantively compromising a facility’s Human Research Protection Program (HRPP) or research information security program.

Relatedness

Relatedness involves an assessment of the degree of causality (attributability) between the study intervention and the event. Site investigators will be asked to provide an assessment of relatedness of the event to the study intervention. The assessment provided by the site investigator is part of the information used by the sponsor (CSP) to determine if the event represents an alteration in the safety profile of the study intervention. All events with a reasonable causal relationship to the study intervention should be considered “related”. A definite relationship does not need to be established. This study will use the following relatedness scale to categorize an event:

- *Definitely Related*: The event is clearly related to the study intervention – i.e. an event that follows a reasonable temporal sequence from administration of the study intervention follows a known or expected response pattern to the suspected intervention that is confirmed by improvement on stopping and reappearance of the event on repeated exposure and that could not be reasonably explained by the known characteristics of the patient’s clinical state.
- *Possibly Related*: An event that follows a reasonable temporal sequence from administration of the study intervention follows a known or expected response

pattern to the suspected intervention, but that could readily have been produced by a number of other factors.

- *Not Related*: The event is clearly not related to the investigational agent/procedure. - i.e. another cause of the event is most plausible; and/or a clinically plausible temporal sequence is inconsistent with the onset of the event and the study intervention and/or a causal relationship is considered biologically implausible.

Unanticipated Adverse Event:

An AE that is new or greater than previously known, in terms of nature, severity, or frequency of occurrence, or any other unanticipated serious problem associated with the investigation that relates to the rights, safety, or welfare of subjects, as documented in the protocol or other materials approved by the IRB of record or the characteristics of the study population. Such materials may include but are not limited to: the informed consent form, safety plan, clinical investigator's brochure (or CSPCPRCC's Drug Information Report), and product labeling (see VHA Handbook 1058.01). The Sponsor (Cooperative Studies Program) will concur or disagree with the site investigator's assessment as to whether an adverse event is anticipated or unanticipated (sometimes referred to as expected/unexpected, respectively).

Expedited Reporting of Serious Adverse Events

All SAEs will be reported by submission of the event into the HITCH reporting system *within 3 calendar days of the Study Site Personnel becoming aware of the event.* The reporting system will immediately inform the Study Biostatisticians, Clinical Research Pharmacists and Study Chairs. All SAEs found to be attributable or related to the study intervention will be documented as "possibly related" or "related" within the SAE Form.

Expedited Reporting of Adverse Events of Special Interest

Cardiac arrhythmias and thromboembolic events will be considered AEs of special interest. Any cardiac arrhythmia or thromboembolic event of grade 3-5 according to Common Terminology Criteria for Adverse Events version 5.0 (CTCAE v5.0; see Appendix C) will be reported in a manner similar to SAEs, whereby the event will be reported to the HITCH reporting system within 3 calendar days of the Study Site Personnel becoming aware of the event.

Reporting of Related and Unexpected Serious Adverse Events

All SAEs will be assessed by the study Sponsor to determine if an event is anticipated or unanticipated. The source of information that will be utilized in the determination of expectedness will include the HITCH Drug Information Report, the HITCH Informed Consent, published literature, and the FDA Adverse Event Reporting System (AERS). SAEs found by the Sponsor to be both related to the investigational treatment and unexpected will be reported to the VA Chief Research and Development Officer, the FDA, and site investigators after review by the Study Chairs, VA Central IRB, the CSPCRPCC Director and the Perry Point CSPCC Director.

Reporting of Adverse and Serious Adverse Events to the DMC

The HITCH Biostatisticians, with coordination from CSPCRPCC, will tabulate and summarize all study intervention related AEs and all SAEs (intervention related or not

related) for the DMC on a schedule set by the DMC, but no less than annually. The DMC will also determine when the committee should be unblinded to treatment assignment in reviewing AE/SAE data. The DMC will advise the CSP Director whether the study should continue or be stopped for safety reasons.

Reporting Requirements of the VA Central or Local IRB

Sites are additionally responsible for following the VA Central IRB policy in submitting safety data, unanticipated serious problems, and protocol deviations. The VA Central IRB's most recent policy including a Table of Reporting Requirements, instructions, and forms can be found at http://www.research.va.gov/vacentralirb/policies.cfm.

VHA Handbook 1058.01 requires that the VA CIRB must be notified orally and immediately upon becoming aware of any research death that is both unanticipated and related (includes possibly related) to the research. In addition to the oral notification, site personnel must also ensure that a written notification is sent to the VA CIRB within 5 business days of becoming aware of the death (Note: this is different than the 3 calendar day reporting requirement to CSP).

Reporting to the FDA

Any FDA reporting requirements will be handled by the sponsor (CSP), not by individual sites. In addition to prompt reporting to appropriate VA personnel, CSPCRPCC will report SAEs that are both related to study intervention and unexpected to the FDA no later than 15 days from when they are notified. If the SAE is a death or a life-threatening event, this notification will happen within 7 days. In addition, the FDA will be notified (1) for any subject who is withdrawn for safety reasons and (2) for any DMC decision to pause enrollment or terminate the study.

9. SAFETY MONITORING

Data and Safety Monitoring Plan

The VA Clinical Sciences Research and Development (CSR&D) centralized Data Monitoring Committee (DMC) will monitor this study. The CSR&D DMC provides an ongoing independent evaluation of the progress of studies, including patient accrual and retention, adverse events monitoring, and analysis plan. This is a service that is provided by CSR&D to ensure independent oversight of the safety and integrity of this project. No other DMC or Data and Safety Monitoring Board (DSMB) review is needed.

The DMC office is located within the Cooperative Studies Program Coordinating Center (CSPCC) at the Edward Hines, Jr. VA Hospital in Hines, IL. The DMC Office at Hines then communicates directly with the PI to provide DMC related information.

After the DMC has recommended approval for this trial to begin and the first patient has been randomized, a progress report will be due in approximately one month and then monthly thereafter. The PI typically will not need to call in for the DMC review after the initial review. The DMC makes recommendations to the director of CSR&D for

endorsement. The recommendations range from approval (unconditionally or with conditions to be addressed) to probation to termination.

Following each DMC meeting, the PI will receive meeting minutes containing any action items for which a response will be requested typically within 30 days. Then the process starts all over again for the next reporting period. In addition to the quarterly progress reports, all Serious Adverse Events are to be reported to the DMC.

Additional Safety Monitoring for AEs of Special Interest

Whereas the DMC serves as the entity with primary oversight responsibility for the study, additional real-time monitoring will be conducted to capture AEs of special interest, including cardiac arrhythmias and thromboembolic events that may result from degarelix. In particular, cardiac arrhythmias and thromboembolic complications of grades 3-5 according to CTCAE v5.0 (see Appendix C) will be closely monitored through not only ongoing, daily review of AE and SAE reports, but also through regular, planned teleconference calls to include:

- The study chair (Dr. Rettig), co-chair (Dr. Nickols), and the lead site coordinator (Ms. Samantha Tran),
- One or more investigators from each site and/or one or more site coordinators.

The teleconferences will occur on Tuesday of each week until the LPI + 30 days. In addition, study sites should report the onset arrhythmias of thromboembolic events of grade 3-5 according to CTCAE v5.0 within 72 hours by contacting the study chair or co-chair by email and voice call (see page 5 for contact information).

10. STATISTICAL CONSIDERATIONS

This is a randomized, placebo-controlled, double-blind phase 2 study that will compare the effects of degarelix + BCS to placebo + BCS on the clinical outcomes of patients hospitalized due to COVID-19.

Expected Treatment Effects

In a recent antiviral drug trial to treat adults hospitalized with severe COVID-19 published in the New England Journal of Medicine (NEJM) on March 18, 2020 (Cao *et al*. 2020), the results showed that a mortality rate of 17%, hospital stay rate of 50%, and ECMO or mechanical intubation rate of 11% among the patients managed with BSC after two-week follow-up. In the current study, we propose a composite endpoint of mortality, ongoing need for hospitalization, and ECMO or mechanical intubation at 15 days after randomization. We are expecting to reduce the composite endpoint rate to 35% after the degarelix treatment plus BSC from 60% for patients treated placebo plus BSC.

Sample Size Calculation and Power Analysis

The sample size estimation and power analysis shown in Table 2 are based on the hypothesis testing of the primary endpoint, which is the composite endpoint of mortality, hospital stay rate, and ECMO or mechanical intubation at 15 days after randomization (Day 15).

According to the composite endpoint outlined in the previous section, we assume that an effect size for the primary endpoint of 42% can be anticipated in the degarelix group. The other assumptions include a three-month accrual time and one-month follow-up time, and 2:1 sample allocation of degarelix: placebo treatment group. The sample size for the study is estimated based on a superiority trial design. To have 90% power of detecting the expected 42% reduction using a two-sided two proportion test with a significance level of 0.05 in the degarelix compared to the placebo group will require 186 evaluable patients total (i.e. 124 evaluable patients in the degarelix group and 62 evaluable patients in the placebo group). Based on an assumed 5% attrition rate, 198 patients will be required (i.e. 132 in the degarelix group and 66 in the placebo group) to achieve actual statistical significance at alpha level of 0.05 and power of 90% (see Table 2).

Table 2: Sample Size Estimation and Power Analysis

Effect Size	Treatment Arms	Testing Power		
		80 %	85 %	90 %
30 %	Degarelix + BSC	186	210	244
	Placebo + BSC	93	105	122
	Total	279	315	366
42%	Degarelix + BSC	96	108	124
	Placebo + BSC	48	54	62
	Total	144	162	186
50%	Degarelix + BSC	64	72	84
	Placebo + BSC	32	36	42
	Total	96	108	126

Duration of Study and Number of Participating Sites

To estimate the expected study duration and resources, we assume that the recruitment rate will be half of what we anticipate. Under this assumption, various scenarios were examined in order to identify an optimal combination between the number of sites, the study duration (which includes recruitment period and follow-up period) and the estimated budget. The study will include four VA medical centers and each center will randomize 17 patients per month on average; however, estimated recruitment rate will vary depending on the patient volume of each participating center as the disease evolves. Based on these assumptions, the recruitment period was found to be approximately three

months. This will be followed by approximately one month of follow up for the last patients randomized into the study followed by one month of data analysis. Thus, the study duration including start-up will be approximately five months.

Interim Analysis and Criteria for Study Termination

A mid-term interim analysis of the primary endpoint will be performed at half of the patients or 99 randomized patients complete or are terminated the study. If the mid-term analysis of the primary endpoint indicates that the null hypothesis can be rejected with a boundary value of 2.77 (standardized Z > 2.77 or < - 2.77) at an α-level of 0.006 or accepted with a boundary value of 0.44 (- 0.44 ≤ standardized Z ≤ 0.44) based on O'Brien and Fleming criteria, the study will be recommended for trial termination for efficacy and futility reasons (shown in the Figure 1). Enrollment will be paused if the interim analysis results indicate crossing of the threshold for futility or efficacy until the final DMC recommendation is rendered.

Meanwhile, the drug toxicity will be closely monitored and analyzed. As described in previous sections, arrythmia and thromboembolic complications are adverse events of special interest that will be monitored closely and graded according to on CTCAE 5.0. Once the toxicity markers reach a threshold of 25% (Ray and Rai 2011), a statistical analysis of the toxicity will be performed. If the experimental (degarelix) group has a significant higher toxicity compared to the placebo group at an α-level of 0.01, then results will be reported to the DMC for a recommendation for trial termination.

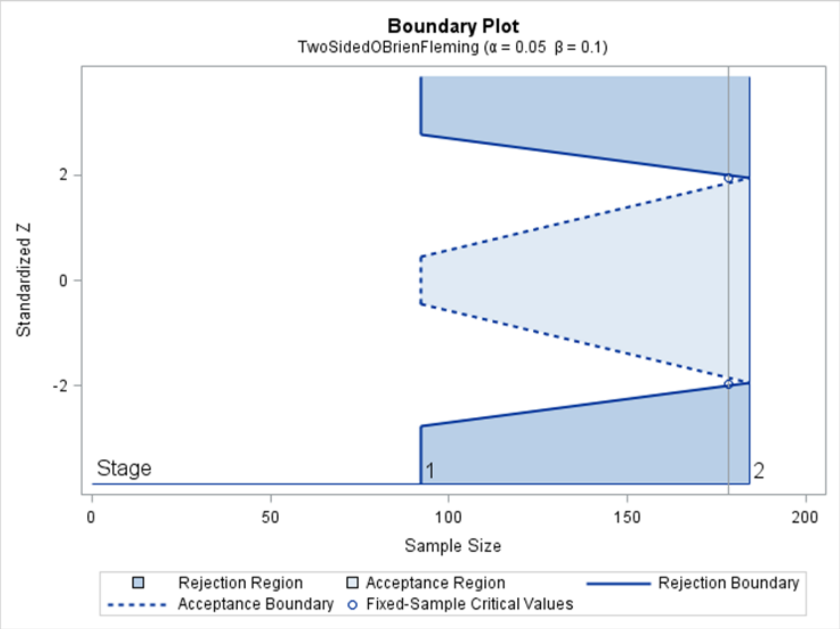

Figure 1. Interim analysis stopping boundaries for efficacy and futility

Data Analysis Plan

The primary analysis will be performed to test the null hypothesis of no difference in composite outcome of mortality, hospital stay rate, and ECMO or mechanical intubation at 15 days after randomization between the degarelix + BSC versus placebo + BSC, i.e. odds ratio equals one. If the null hypothesis is not rejected, a 95% confidence interval will be constructed about the difference observed to inform the medical community as to how large the difference is likely to be in either direction. All statistical tests will be two-sided, and the primary outcome will be tested at 5% level of significance. SAS 9.4 or higher will be used to conduct all the statistical analyses. A variety of analytic methods will be used for the primary endpoint, secondary endpoints, exploratory endpoints analyses, and other analyses (Table 3).

Analysis Populations

Intent-to-Treat (ITT) – This population is defined as the population of patients who will be randomized to either of the treatment groups – degarelix or placebo. The patients will be categorized (in terms of their treatment assignment) based on their initial randomized group and will be included in analyses irrespective of their status – completer or drop out of the study before completion. The testing power for the primary endpoint is estimated as 90% in this population.

Safety – This population includes all patients who will be randomized to either of the treatment groups – degarelix or placebo.

The primary analysis of the study will be performed on the primary endpoint on the ITT population. SAEs will be analyzed based on the Safety population.

Primary Endpoint Analysis

The primary study endpoint will be the composite outcome of mortality, ongoing need for hospitalization, and ECMO or mechanical intubation at 15 days after randomization. This outcome will be compared according to assigned treatment groups, using Pearson χ^2 test. The test for differences between the treatment groups in the primary outcome will be conducted at an overall α-level of 0.05 (i.e. α = 0.006 for mid-term interim analysis, α = 0.044 for the final analysis). If a patient drops out from the study, then he will be considered as a failure. Additional analyses will be conducted using logistic models to adjust for other clinical factors, such as age, hypertension, and COPD. Logistic regression will be used for the primary endpoint (y = 1 if a composite outcome, otherwise y = 0) analysis with the treatment group as the testing factor (x). The following covariates will be included in the model: age (z1), hypertension (z2), and COPD (z3), site (z4). Given the composite outcome probability $p = p\ (y = 1|x, z_1, z_2, z_3)$, the basic model is defined as follows:

$$Logit(p) = ln\left(\frac{p}{1-p}\right) = \beta_0 + \beta_1 x + \beta_2 z_1 + \beta_3 z_2 + \beta_4 z_3$$

Odds ratio and 95% confidence interval (CI) will be presented using SAS PROC GENMOD or PROC LOGSTIC. If the coefficient for treatment effect is significant, then

the null hypothesis will be rejected. Logistic models will be tested for goodness of fit. We will assess goodness-of-fit using the statistic -2 log likelihood, which has a χ^2 distribution under the null hypothesis that all the explanatory variables in the model are zero. We will also consider the Akaike Information Criterion statistic and the Schwartz Criterion statistic, both of which adjust the -2-log likelihood for the number of items in the model. Models that show lack of fit will be reconsidered for the inclusion of additional variables or use of alternate models with assumptions that are better met by the study data. One alternate model if model fit is poor for logistic regression is a log-linear model.

Further, Cochran Mantel-Haenszel method will be used to adjust for three randomization stratification factors (Age: <65 vs. ≥65, History of hypertension: yes vs. no, and Influenza severity scale: 3 vs 4/5) using SAS PROC FREQ as one of the sensitivity analyses for the primary efficacy endpoint.

Secondary Endpoint Analyses

Secondary endpoints included in the data analysis are the composit endpoint at 30 days after randomization, time to clinical improvement, inpatient mortality, length of hospital stay, length of intubation for mechanical ventilation, time to normal temperature, and the maximum severity of COVID-19 illness, which are defined in the endpoint section. The secondary endpoint analyses will be adjusted for multiplicity with a α-level of 0.0071 for each endpoint.

The composite outcome of mortality, ongoing need for hospitalization, and ECMO or mechanical intubation at 30 days after randomization as a secondary endpoint will be analyzed using the methods described in the the primary endpoint analysis.

For the time to clinical improvement as defined by a decline of 2 categories or more from the baseline on the modified 7-category ordinal scale of clinical status of hospitalized influenza patients (influenza scale, see Appendix A) or hospital discharge (see section 2 for definition of discharge) whichever comes first, survival analysis techniques will be used to analyze the time-to-event data for this endpoint. Patients whose conditions getting worse or died or withdraw from the study without clinical improvement will be censored. Kaplan-Meier analysis will be used to compare the two curves of the time to the clinical improvement over the patient follow-up time between the two treatment groups and a log-rank statistic will be used to test the equality of the survival function estimates of the two treatment groups using SAS PROC LIFETEST. Kaplan-Meier curves will be created. Additional analysis will be conducted using the Cox's Proportional Hazards model to test the treatment efficacy of the treatment intervention on the time until endpoint events adjusted for three prognostic factors: age, hypertension, and COPD. The hazard ratio and 95% confidence interval (CI) will be presented using SAS PROC PHREG. If the coefficient for treatment effect is significant, then the null hypothesis will be rejected. The regression model will be checked for the model assumption, adequacy, and the goodness of fit. If the model shows lack of fit, then alternate models with assumptions that are better met by the study data will be considered.

For mortality endpoint data analysis, the treatment effect will be analyzed initially with a Pearson χ^2 test and logistic regression will also be performed using SAS PROC

LOGISTIC by taking account of prognostic factors described in the primary endpoint analysis.

For the length of hospital stay data analysis, medians (interquartile ranges) will be presented and Wilcoxon tests, a nonparametric method, will be performed to compare the medians of the length of hospital stay between the two treatment groups using SAS PROC NPAR1WAY. In addition, a quantile regression will be used to test the effect of the treatment on the time until the clinical event adjusted for prognostic factors described in the primary endpoint analysis.

For the length of intubation for mechanical ventilation data analysis, nonparametric analyses will be performed as described in the length of hospital stay analysis.

For time to normal temperature data analysis, survival analysis techniques will be used as described in the time to the clinical endpoint analysis using SAS PROC LIFETEST and PROC PHREG.

For the maximum severity of COVID-19 illness data analysis, Pearson χ^2 test will be performed for this categorical endpoint using SAS PROC FREQ initially. Given the endpoint is also an ordinal variable, Cochran–Armitage test will be performed to test the ordinal trend tendency using SAS PROC FREQ. in addition to the frequency analysis, proportional odds logistic regression will also be performed using SAS PROC LOGISTIC by taking account of the factors of age, hypertension, COPD, and the baseline influenza scale (see Appendix A).

Exploratory Endpoint Analyses

<u>Clinical and Laboratory Prognostic Factors</u>

Exploratory analyses will be performed to identify the impact of clinical and laboratory prognostic factors on the primary endpoint or any secondary endpoint. Clinical factors include age, presence or absence of COPD, hypertension, or cardiovascular disease, or use of angiotensin converting enzyme inhibitors. Laboratory measurements including D-dimer, IL-6, LDH, ferritin, total WBC, absolute neutrophil count (ANC), absolute lymphocyte count (ALC), and testosterone are measured during the patient's hospital stay. Further germline genomic factors will also be analyzed if feasible as prognostic factors for the study outcomes for part of the patients.

For continuous variables, the sample size, mean, SD, median, minimum, and maximum values for each intervention will be presented for each parameter at each data collection time point and tested using SAS PROC TTEST between the treatment groups. If a variable is not normally distributed the non-parametric method will be applied using SAS PROC NPAR1WAY. For categorical variables, the number and percentage of patients by the treatment group will be tabulated and tested based on Pearson χ^2 test using SAS PROC FREQ.

Univariate correlation with the primary endpoint or any categorical secondary endpoint will be assessed for each clinical and laboratory factor based on logistic regressions using SAS PROC LOGISTIC. For the secondary endpoints of time to events data, univariate analysis will be performed based on Cox regressions using SAS PROC PHREG. For the secondary endpoints of length of hospital stay and length of intubation for mechanical ventilation, nonparametric univariate analysis will be performed based on Spearman correlation method using SAS PROC CORR for the continuous prognostic factors, whereas Mann–Whitney U or Wilcoxon rank-sum test will be performed for the categorical prognostic factors using SAS PROC NPAR1WAY. For those prognostic factors significantly associated with the primary and secondary outcomes from the univariate analyses, multiple regressions will be performed by adjusting some other prognostic factors identified from the univariate analyses. The multiple regressions will be performed as described in the primary analysis and each corresponding secondary endpoint analysis.

Viral Load, Cytokines Levels, and TMPRSS2 Expression

To explore the mechanisms that underlie treatment effect, nasopharyngeal (NP) epithelial or peripheral blood mononuclear cell (PBMC) TMPRSS2 expression, NP and plasma viral load, or serum cytokine (TNF-α, IL-1β, IL-6) levels will be tested among the patients from the Great Los Angeles site, only.

Viral load kinetics in the two treatment groups over patients' critical period will be monitored from baseline to Day 15. The viral load in plasma will be assessed by the viral RNA measured by a reverse-transcriptase–polymerase chain--reaction (RT-PCR) assay and quantified as log_{10} copies/ml. Mean change from baseline in SARS-CoV-2 viral RNA load across each sample collection time point will be compared by the treatment groups. The mean value for log_{10} copies/ml for each week will be calculated and summarized. Log_{10} copies/ml changes from baseline to each week will be summarized. For each week, the sample size, mean, SD, median, minimum, and maximum values for each treatment will be presented. A mixed-effect model of repeated measure (MMRM) for overall changes across all the time point between the two treatment groups using SAS PROC MIXED. A longitudinal graph of Log_{10} copies/ml will be created and presented by 95% confidence intervals of the means.

Serum cytokines TNF-α, IL-1β, and IL-6, as well as TMPRSS2 expression and anti-viral antibodies will be tested by immunoassays. Mean change from baseline in the cytokine and TMPRSS2 levels, and antibody titers across each sample collection time point will be compared by the treatment groups. The mean value for each week will be calculated and summarized. Mean changes from baseline to each week will be summarized. For each week, the sample size, mean, SD, median, minimum, and maximum values for each treatment will be presented. A mixed-effect model of repeated measure (MMRM) for overall changes across all the time point between the two treatment groups using SAS PROC MIXED. A longitudinal graph of each measurement level will be created and presented by 95% confidence intervals of the means. If a measurement level

is not normally distributed, then a non-parametric method will be used as an alternative analysis approach.

The exploratory analyses will not be adjusted for multiplicity.

AE and SAE analyses

Adverse events (AE) and serious adverse events (SAEs) are defined by the ICH for Clinical Safety Data Management (ICH-E2A), the Food and Drug administration as described in the section 9. Incidence of AE/SAEs will be summarized for each treatment group by body system and MedDRA term. The number and percentage of subjects with each body system and MedDRA term will be presented for each group. Pearson χ^2 test and/or Fisher Exact test were used to compare the frequency difference of AE and SAE between the treatment groups in System of Body (SOC) and Preferred Terms (PT) levels. Tables to summarize the incidence rates will be created for each of the following groups: Total AE and SAE, AE and SAE by relationship to study intervention, AE and SAE leading to premature discontinuation, AE and SAE presented in descending order of frequency by MedDRA term (no body systems shown). AE and SAE that led to premature discontinuation from the study will be listed. These listings will contain details about the SAEs such as outcome and relationship to study treatments. Other supportive data, such as the subject’s age, will be given. All AE and SAE will be coded with MedDRA (updated version) and listed by subject.

Other analyses

Baseline characteristics

The patient demographics and pre-treatment baseline characteristics will be summarized for each treatment group and for all patients. The patient demographics such as age, race, gender, ethnicity etc. and baseline test results such as D-dimer, IL-6, LDH, ferritin, total WBC, absolute neutrophil count (ANC), absolute lymphocyte count (ALC), and testosterone will be analyzed. For continuous variables, the sample size, mean, median, SD, minimum, and maximum values will be calculated and tested either by Student t or Wilcoxon test depending on data distributions using SAS PROC TTEST and SAS PROC NPAR1WAY, respectively. For categorical variables, the number and percentage of patients by the intervention group will be tabulated and tested based on Pearson χ^2 test using SAS PROC FREQ.

Disposition status

Subject disposition will be summarized for the ITT population. The number and percentage of patients who completed or discontinued prematurely from the study by the treatment group will be tabulated and tested using Pearson χ^2 test. The number and percentage of patients who discontinued for each reason will be presented for each intervention group. The number and percentage of patients who completed or discontinued prematurely in each intervention group will also be displayed graphically.

Adherence

Patients who change the treatment groups after randomization or have protocol violations will be identified as non-adherence to the treatment. The number and percentage of non-adherence patients will be summarized by the treatment group and tested using Pearson χ^2 test.

Site Effect

Site effect will be evaluated for four participating sites. Balances of the treatment of assignment and the primary and secondary outcomes will be assessed among the four sites. If there is significant associations between the site and treatment assignment and a study outcome at significance level of 0.05 without multiplicity adjustment, the site effect will be adjusted in the corresponding the primary or any secondary outcome along with the other prognostic factors described in the primary and secondary analyses.

Table 3: Summary of Data Analysis Plan

Endpoint Analyses	Statistical Methods	SAS Procedures
Primary Endpoint<br>A composite endpoint of mortality, hospital stay rate, and ECMO or mechanical intubation at 15 days after randomization	Pearson Chi-square tests or Fisher exact tests<br>Logistic regression adjusted for Age, Hypertension, and COPD	. PROC FREQ<br>PROC LOGISTC
Secondary Endpoints<br>1. A composite endpoint of mortality, hospital stay rate, and ECMO or mechanical intubation at 30 days after randomization<br>2. Time to the clinical improvement<br>3. Inpatient Mortality<br>4. Length of Hospital Stay<br>5. Duration of Intubation for Mechanical Ventilation<br>6. Time to Normal Temperature<br>7. Maximum Severity of COVID-19 Illness	1. Pearson Chi-square tests or Fisher exact tests<br>Logistic regression adjusted for Age, Hypertension, and COPD<br>2. Log-rank test, Kaplan-Meier curves<br>Cox regression adjusted for Age, Hypertension, and COPD<br>3. Pearson Chi-square tests or Fisher exact tests<br>Logistic regression adjusted for Age, Hypertension, and COPD<br>4. Wilcoxon test; quantile regression<br>5. Wilcoxon test; Quantile regression<br>6. Log-rank test, Kaplan-Meier curves<br>Cox regression adjusted for Age, Hypertension, and COPD<br>7. Pearson Chi-square test; Cochran–Armitage trend test<br>Proportional odds logistic regression	1. PROC FREQ<br>PROC LOGISTC<br>2. PROC LIFETEST<br>PROC PHREG<br>3. PROC FREQ<br>PROC GENMOD<br>4.5.PROC NPAR1WAY<br>PROC QUANTREG<br>6. PROC LIFETEST<br>PROC PHREG<br>7. PROC FREQ<br>PROC LOGISTC
Exploratory Analyses<br>Clinical Prognostic Factors<br>Laboratory Prognostic Factors<br>Viral Load<br>Cytokines Levels and TMPRSS2 Expression<br>Germline genomic factors	1. Time to event data<br>Log-rank tests, Kaplan-Meier curves and Cox regressions<br>2. Categorical data<br>Pearson Chi-square tests and Logistic regression<br>3. Interval data<br>Student t / Wilcoxon tests, linear or quantile regressions<br>4. Longitudinal data<br>Mixed-effect model repeated measure	1. PROC LIFETEST<br>PROC PHREG<br>2. PROC FREQ<br>PROC LOGISTC<br>3. PROC TTEST<br>PROC NPAR1WAY<br>4. PROC Mixed
AE/SAE Analyses<br>1. Incidence of AE/SAE<br>2. Frequency Difference of AE and SAE<br>3. AE /SAE by Relationship to the Treatment<br>4. AE/SAE leading to premature discontinuation	For All AE/SAE<br><br>Incidence rate estimation and testing<br>Pearson Chi-square tests or Fisher exact tests	PROC FREQ<br>SAS Macros
Other Analyses<br>1. Demographics and Baseline Characteristics<br>2. Disposition Status<br>3. Study Protocol Adherence<br>4. Site Effect	1. Student t or Wilcoxon tests,<br>Pearson Chi-square or Fisher Exact Tests<br>2. Pearson Chi-square Tests<br>3. Pearson Chi-square Tests<br>4. Pearson Chi-square Tests, Student t or Wilcoxon tests,<br>log-rank test	1. PROC TTEST,<br>PROC FREQ,<br>PROC NPAR1WAY<br>2. PROC FREQ<br>3. PROC FREQ<br>4. PROC FREQ<br>PROC LIFETEST<br>PROC NPAR1WAY

Handling of Missing Data

Every effort will be made to minimize the occurrence of missing data, particularly for the primary and the secondary endpoints. In the event of a potential drop out, every effort will be made to capture missed data from the patient record and VA databases. For patients who drop out during the study the missing values for the primary endpoint analysis will be considered as failures. However multiple imputation (MI) method may be used for certain secondary endpoint analyses. Multiple imputations will be performed using SAS PROC MI under missing at random assumption. If the assumption does not hold, then imputations will be performed. Sensitivity analysis will be performed to compare the results from the various imputation scenarios.

11. DATA COLLECTION AND QUALITY ASSURANCE

Data Collection Forms

Data will be collected for each patient as described in the Table of Schedule of Events. Electronic case report forms (eCRFs) will be completed for screening, Days 1, 8, 15, 22, and 30 and then weekly until discharge (discharge being defined as no longer requiring hospitalization for COVID-19) or death.

Data Management

Access to individual identifiable patient information will be available to the PI, the co-PIs, and study staff. eCRFs will be generated to track clinical, laboratory, and radiographic data. Each study site will maintain records for its patients. Each site will have access only to data entered at its respective site. Investigators will not have access to unblinded study data.

The Perry Point CSPCC will serve as the Data Coordinating Center for this trial. Data collection will be accomplished using the Clinical Data Management System (CDMS), *iDataFax*, licensed by DF/Net. *iDataFax* is a hybrid data management system that can collect data from both paper and electronic sources, as well as allow for direct data entry. Direct data entry will be permitted in this study. Data will be entered from corresponding source documentation and from paper case report forms (CRFs) designed for this trial. Patient medical records and case report forms will serve as the original sources of data for verification. Data from these sources will be entered and submitted and after submission will be reviewed by Perry Point Clinical Data Managers. The end users will access the CDMS with credentials issued to them from the Perry Point CSPCC; these credentials are dependent on their roles at the site and on their completion of required data management, privacy protection, human subjects protection (HSP), and good clinical practices (GCP) training.

Given the urgency of this trial and the impossibility of holding face-to-face meetings, there will be no formal kick-off meeting for HITCH. Moreover, due to the anticipated duration of the study, there will be no annual meeting. Rather, the study PI will have teleconference meetings with each of the site investigators, as a group or individually depending on availability, and the study staff. The study will be read in detail by all site investigators prior to the teleconferences. The study site investigators will complete online good clinical practices (GCP) and information security training. Data management training will take place via teleconference. A comprehensive Data Management Handbook will be furnished as part of the study's approved Operations Manual. These tools will be made available to site personnel via SharePoint, an online file sharing system selected for use in this study because of its acceptance within the VA and its ability to store study documents behind the security of the VA firewall. Blank original CRFs and related source document templates will also be furnished to participating clinical sites via Microsoft SharePoint, so that they may be accessed and duplicated on demand, dependent on individual sites' needs and rates of recruitment.

Site personnel are expected to enter source data into the CDMS on an ongoing basis in this study, timed as closely with the required visits and procedures as possible. The Perry Point CSPCC will monitor the timeliness and accuracy of data submission and will provide performance data to the study's Principal Investigator for routine review, or upon request.

Following submission to the CDMS, CRF data must pass through a series of quality filters before it is deemed acceptable into the main study database. First, a series of automated logic and range checks will be applied to the data upon data entry and submission and will result in automated requests for confirmation or correction by site personnel. A second series of Quality Checks (QCs) will occur after data submission and will consist of more sophisticated validity checks customized by the clinical data programming staff at the Perry Point CSPCC. These checks may compare values across several different CRFs for consistency; they may trigger requirements for additional forms; or they may calculate necessary values or scores. Reports and listings of data requiring clarifications may also be issued by the data management staff at the Perry Point CSPCC when potential data integrity issues are identified outside of automated means, through a centralized monitoring approach. The Clinical Research Monitors assigned to verify CRF data submissions with source documentation may also issue requests to site personnel for data confirmation, clarification or correction.

If incomplete or inaccurate data or data practices are found, site personnel will be notified and given the opportunity to confirm, correct or clarify the data at the earliest time possible following detection. Only data that have undergone these quality control procedures will be accepted into the study's final database. All confirmations, clarifications and corrections made to CRF data must accord with existing source documentation and must be made in accordance with the ICH guidelines for good clinical practice (GCP). The procedures for confirming, clarifying, or correcting CRF data will be demonstrated at the study's teleconference training meetings and will be detailed within the Data Management Handbook, part of the study's Operations Manual.

All local site investigators (LSI) are responsible for maintaining accurate, legible, complete and up-to-date source documentation and corresponding CRF entries for each patient, including any films, lab reports, ECG tracings, and electronic storage media. All known deviations from the approved study protocol must be documented by site personnel on the protocol deviation CRF and must be acknowledged with a signature from the LSI. Corrections or other changes to the CRFs must made in line with GCP guidelines using established Perry Point CSPCC procedures.

Upon the completion of this clinical trial when all data have been entered and all queries and requests for data clarifications have been resolved, or the attempts to resolve them have been exhausted, the CDMS will be locked from further editing. Following lock of the trial database, data management staff at the Perry Point CSPCC will furnish final analytical datasets to the study's biostatistician for analysis. Statistical analysis of the data will be performed in accordance with the study's protocol.

Patient Discontinuation:

Patients will be discontinued from study participation under the following circumstances:

- Death or Day 60 (whichever comes first)
- Patient withdraws consent for any reason

Quality Assurance

Training

All staff has and will continue to undergo appropriate training related to human subjects. Access to study data is limited to only personnel listed as research staff. All study staff must be compliant with all required VA human subjects' trainings (Privacy and HIPAA, Information Security Awareness & Rules of Behavior, CITI GCP).

Protocol Deviations

Protocol deviations will be recorded by site personnel at the site at which they occur and will be documented both with a note to chart and on the Protocol Deviation CRF. Deviations will be submitted via CRF to the VA CSPCC, Perry Point and will become part of the study's database. The VA CSPCC will report out protocol deviations to the PI at the coordinating site (VA GLA) on a routine (monthly) basis, or upon request.

12. PATIENT RIGHTS AND CONFIDENTIALITY

This study will be conducted in accordance with the ethical principles that have their origin in the Declaration of Helsinki as stated in 21 CFR §312.120(c)(4); consistent with GCP and all applicable regulatory requirements. The Investigator must comply with the applicable regulations in Title 21 of the Code of Federal Regulations (21 CFR §50, §54, and §312), GCP/ICH guidelines, and all applicable regulatory requirements. The IRB must comply with the regulations in 21 CFR §56 and applicable regulatory requirements.

Institutional Review Board (IRB) Review

This protocol and the informed consent document and any subsequent modifications will be reviewed and approved by the VA IRB responsible for oversight of the study. Subject recruitment materials and any other written information to be provided to subjects are also reviewed and approved by the IRB before any protocol related procedures are performed on any subjects.

Informed Consent Form

A signed research consent form (VA Form 10-1086) must be obtained from each patient, or from his legally authorized representative (LAR), if the patient is unable to provide informed consent. The consent form will describe the purpose of the study, the procedures to be followed, and the risks and benefits of participation as well as the required elements per VHA regulation. A copy will be given to each patient or LAR and this fact will be documented in the patient's record.

The legally authorized representative (LAR) may only provide informed consent for the patient to participate in the study. Examples of a LAR include, but may not be limited to, a spouse, parent, adult child over 18 years of age, or court-appointed individual (i.e. legal guardian, POA).

Patient Confidentiality

All patient reports and clinical samples will be identified by an assigned coded number/letter to maintain patient confidentiality (e.g. sample 1B). The PI will maintain a

log of patients' codes, names, and contact information, which will be kept in a locked cabinet in his office, which in turn will remain locked in his absence. The PI has a large, heavy-duty combination code protected safe in his office for the storage of these documents. Every effort will be made to keep all documents with patient identifiers under the strictest confidentiality. Information that is collected during the study will be stored at the research site: paper copies will be kept in the safe, and computer files will be protected by passwords.

The study was granted a waiver of HIPAA Authorization for the entire study, authorizing the use and disclosure of identifiable health information relevant to this study. This information comes from medical records and research study-specific information that is obtained for the purposes of this study.

Patient records, the research information, and the informed consent forms may be inspected by a representative of the Department of Veteran Affairs, Office of Human Research Protections, the Government Accountability Office, the Office of the Inspector General, the VA Office of Research Oversight, the VA Central IRB, local Research and Development Committee, Food and Drug Administration, and U.S. Governmental Agencies. Thus, because of the possibility that information may be released to one of these regulatory institutions, absolute confidentiality cannot be guaranteed. Results of this study may be published, but the names or identities of subjects will not be revealed, and medical records will remain confidential unless the disclosure is required by law.

Research study files will be kept in accordance with the VA Records Control Scheduled (RCS 10-1). At study closure, data and samples will be deidentified. Remaining samples will be stored indefinitely for other, potential research, unless a patient indicates in the informed consent form that remaining samples must be destroyed upon completion of the research study.

Study Discontinuation

The study may be discontinued at any time by the IRB, the OHRP, the FDA, or other government agencies as part of their duties to ensure that research patients are protected.

Clinical Criteria for Early Trial Termination

The VA Data Monitoring Committee will oversee the trial and make determinations related to early trial termination.

13. FUNDING AND INSURANCE

This study is funded by the Department of Veterans Affairs through a Merit Review grant. This grant and the VA cover the costs of executing this study.

14. PUBLICATION OF RESEARCH FINDINGS

Publication of the results of this trial will be governed by the policies and

procedures of the Human Research Protections Programs (HRPPs) of the participating sites. Patient confidentiality will be maintained in any presentation, abstract, or manuscript.

15. REFERENCES

1. Wang Y, Fan G, Salam A, Horby P, Hayden FG, Chen C, Pan J, Zheng J, Lu B, Guo L, Wang C, Cao B, CAP-China Network. Comparative effectiveness of combined favipiravir and oseltamivir therapy versus oseltamivir monotherapy in critically ill patients with influenza virus infection. J Infect Dis. 2019 Dec 11; PMID: 31822885

2. Zhu N, Zhang D, Wang W, Li X, Yang B, Song J, Zhao X, Huang B, Shi W, Lu R, Niu P, Zhan F, Ma X, Wang D, Xu W, Wu G, Gao GF, Tan W, China Novel Coronavirus Investigating and Research Team. A Novel Coronavirus from Patients with Pneumonia in China, 2019. N Engl J Med. 2020 20;382(8):727–733. PMID: 31978945

3. Wu A, Peng Y, Huang B, Ding X, Wang X, Niu P, Meng J, Zhu Z, Zhang Z, Wang J, Sheng J, Quan L, Xia Z, Tan W, Cheng G, Jiang T. Genome Composition and Divergence of the Novel Coronavirus (2019-nCoV) Originating in China. Cell Host Microbe. 2020 11;27(3):325–328. PMID: 32035028

4. Wang C, Liu Z, Chen Z, Huang X, Xu M, He T, Zhang Z. The establishment of reference sequence for SARS-CoV-2 and variation analysis. J Med Virol. 2020 Mar 13; PMID: 32167180

5. Li Q, Guan X, Wu P, Wang X, Zhou L, Tong Y, Ren R, Leung KSM, Lau EHY, Wong JY, Xing X, Xiang N, Wu Y, Li C, Chen Q, Li D, Liu T, Zhao J, Liu M, Tu W, Chen C, Jin L, Yang R, Wang Q, Zhou S, Wang R, Liu H, Luo Y, Liu Y, Shao G, Li H, Tao Z, Yang Y, Deng Z, Liu B, Ma Z, Zhang Y, Shi G, Lam TTY, Wu JT, Gao GF, Cowling BJ, Yang B, Leung GM, Feng Z. Early Transmission Dynamics in Wuhan, China, of Novel Coronavirus-Infected Pneumonia. N Engl J Med. 2020 26;382(13):1199–1207. PMID: 31995857

6. van Doremalen N, Bushmaker T, Morris DH, Holbrook MG, Gamble A, Williamson BN, Tamin A, Harcourt JL, Thornburg NJ, Gerber SI, Lloyd-Smith JO, de Wit E, Munster VJ. Aerosol and Surface Stability of SARS-CoV-2 as Compared with SARS-CoV-1. N Engl J Med. 2020 Mar 17;0(0):null.

7. Wang D, Hu B, Hu C, Zhu F, Liu X, Zhang J, Wang B, Xiang H, Cheng Z, Xiong Y, Zhao Y, Li Y, Wang X, Peng Z. Clinical Characteristics of 138 Hospitalized Patients With 2019 Novel Coronavirus-Infected Pneumonia in Wuhan, China. JAMA. 2020 Feb 7; PMCID: PMC7042881

8. Madjid M, Safavi-Naeini P, Solomon SD, Vardeny O. Potential Effects of Coronaviruses on the Cardiovascular System: A Review. JAMA Cardiol. 2020 Mar 27; PMID: 32219363

9. Patrick Walker. The Global Impact of COVID-19 and Strategies for Mitigation and Suppression.WHO Collaborating Centre for Infectious Disease Modeling. Institute for Disease and Emergency Analytics, Imperial College London; 2020.

10. Chen W-H, Strych U, Hotez PJ, Bottazzi ME. The SARS-CoV-2 Vaccine Pipeline: an Overview. Curr Trop Med Rep. 2020 Mar 3;1–4. PMCID: PMC7094941

11. Zhang C, Huang S, Zheng F, Dai Y. Controversial treatments: an updated understanding of the Coronavirus Disease 2019. J Med Virol. 2020 Mar 26; PMID: 32219882

12. Gautret P, Lagier J-C, Parola P, Hoang VT, Meddeb L, Mailhe M, Doudier B, Courjon J, Giordanengo V, Vieira VE, Dupont HT, Honoré S, Colson P, Chabrière E, La Scola B, Rolain J-M, Brouqui P, Raoult D. Hydroxychloroquine and azithromycin as a treatment of COVID-19: results of an open-label non-randomized clinical trial. Int J Antimicrob Agents. 2020 Mar 20;105949. PMID: 32205204

13. Walls AC, Park Y-J, Tortorici MA, Wall A, McGuire AT, Veesler D. Structure, Function, and Antigenicity of the SARS-CoV-2 Spike Glycoprotein. Cell. 2020 Mar 6; PMID: 32155444

14. Hoffmann M, Kleine-Weber H, Schroeder S, Krüger N, Herrler T, Erichsen S, Schiergens TS, Herrler G, Wu N-H, Nitsche A, Müller MA, Drosten C, Pöhlmann S. SARS-CoV-2 Cell Entry Depends on ACE2 and TMPRSS2 and Is Blocked by a Clinically Proven Protease Inhibitor. Cell. 2020 Mar 4; PMID: 32142651

15. Zhou Y, Vedantham P, Lu K, Agudelo J, Carrion R, Nunneley JW, Barnard D, Pöhlmann S, McKerrow JH, Renslo AR, Simmons G. Protease inhibitors targeting coronavirus and filovirus entry. Antiviral Res. 2015 Apr;116:76–84. PMCID: PMC4774534

16. Bertram S, Heurich A, Lavender H, Gierer S, Danisch S, Perin P, Lucas JM, Nelson PS, Pöhlmann S, Soilleux EJ. Influenza and SARS-coronavirus activating proteases TMPRSS2 and HAT are expressed at multiple sites in human respiratory and gastrointestinal tracts. PloS One. 2012;7(4):e35876. PMCID: PMC3340400

17. Yamaya M, Shimotai Y, Hatachi Y, Lusamba Kalonji N, Tando Y, Kitajima Y, Matsuo K, Kubo H, Nagatomi R, Hongo S, Homma M, Nishimura H. The serine protease inhibitor camostat inhibits influenza virus replication and cytokine production in primary cultures of human tracheal epithelial cells. Pulm Pharmacol Ther. 2015 Aug;33:66–74. PMID: 26166259

18. Vaarala MH, Porvari KS, Kellokumpu S, Kyllönen AP, Vihko PT. Expression of transmembrane serine protease TMPRSS2 in mouse and human tissues. J Pathol. 2001 Jan;193(1):134–140. PMID: 11169526

19. Iwata-Yoshikawa N, Okamura T, Shimizu Y, Hasegawa H, Takeda M, Nagata N. TMPRSS2 Contributes to Virus Spread and Immunopathology in the Airways of

Murine Models after Coronavirus Infection. J Virol. 2019 15;93(6). PMCID: PMC6401451

20. Lucas JM, Heinlein C, Kim T, Hernandez SA, Malik MS, True LD, Morrissey C, Corey E, Montgomery B, Mostaghel E, Clegg N, Coleman I, Brown CM, Schneider EL, Craik C, Simon JA, Bedalov A, Nelson PS. The androgen-regulated protease TMPRSS2 activates a proteolytic cascade involving components of the tumor microenvironment and promotes prostate cancer metastasis. Cancer Discov. 2014 Nov;4(11):1310–1325. PMCID: PMC4409786

21. Lek M, Karczewski KJ, Minikel EV, Samocha KE, Banks E, Fennell T, O'Donnell-Luria AH, Ware JS, Hill AJ, Cummings BB, Tukiainen T, Birnbaum DP, Kosmicki JA, Duncan LE, Estrada K, Zhao F, Zou J, Pierce-Hoffman E, Berghout J, Cooper DN, Deflaux N, DePristo M, Do R, Flannick J, Fromer M, Gauthier L, Goldstein J, Gupta N, Howrigan D, Kiezun A, Kurki MI, Moonshine AL, Natarajan P, Orozco L, Peloso GM, Poplin R, Rivas MA, Ruano-Rubio V, Rose SA, Ruderfer DM, Shakir K, Stenson PD, Stevens C, Thomas BP, Tiao G, Tusie-Luna MT, Weisburd B, Won H-H, Yu D, Altshuler DM, Ardissino D, Boehnke M, Danesh J, Donnelly S, Elosua R, Florez JC, Gabriel SB, Getz G, Glatt SJ, Hultman CM, Kathiresan S, Laakso M, McCarroll S, McCarthy MI, McGovern D, McPherson R, Neale BM, Palotie A, Purcell SM, Saleheen D, Scharf JM, Sklar P, Sullivan PF, Tuomilehto J, Tsuang MT, Watkins HC, Wilson JG, Daly MJ, MacArthur DG, Exome Aggregation Consortium. Analysis of protein-coding genetic variation in 60,706 humans. Nature. 2016 18;536(7616):285–291. PMCID: PMC5018207

22. Kuba K, Imai Y, Rao S, Gao H, Guo F, Guan B, Huan Y, Yang P, Zhang Y, Deng W, Bao L, Zhang B, Liu G, Wang Z, Chappell M, Liu Y, Zheng D, Leibbrandt A, Wada T, Slutsky AS, Liu D, Qin C, Jiang C, Penninger JM. A crucial role of angiotensin converting enzyme 2 (ACE2) in SARS coronavirus-induced lung injury. Nat Med. 2005 Aug;11(8):875–879. PMCID: PMC7095783

23. Clinckemalie L, Spans L, Dubois V, Laurent M, Helsen C, Joniau S, Claessens F. Androgen regulation of the TMPRSS2 gene and the effect of a SNP in an androgen response element. Mol Endocrinol Baltim Md. 2013 Dec;27(12):2028–2040. PMCID: PMC5426606

24. Chen Z, Song X, Li Q, Xie L, Guo T, Su T, Tang C, Chang X, Liang B, Huang D. Androgen Receptor-Activated Enhancers Simultaneously Regulate Oncogene TMPRSS2 and lncRNA PRCAT38 in Prostate Cancer. Cells [Internet]. 2019 Aug 9 [cited 2020 Mar 29];8(8). Available from: https://www.ncbi.nlm.nih.gov/pmc/articles/PMC6721761/ PMCID: PMC6721761

25. Lin B, Ferguson C, White JT, Wang S, Vessella R, True LD, Hood L, Nelson PS. Prostate-localized and androgen-regulated expression of the membrane-bound serine protease TMPRSS2. Cancer Res. 1999 Sep 1;59(17):4180–4184. PMID: 10485450

26. Mikkonen L, Pihlajamaa P, Sahu B, Zhang F-P, Jänne OA. Androgen receptor and androgen-dependent gene expression in lung. Mol Cell Endocrinol. 2010 Apr 12;317(1–2):14–24. PMID: 20035825

27. Wang, X et al. Transcriptional Inhibition of Host Viral Entry Proteins as a Therapeutic Strategy for SARS-CoV-2. Preprints 2020, 2020030360 (doi: 10.20944/preprints202003.0360.v1).; 2020.

28. Cheng Z, Zhou J, To KK-W, Chu H, Li C, Wang D, Yang D, Zheng S, Hao K, Bossé Y, Obeidat M, Brandsma C-A, Song Y-Q, Chen Y, Zheng B-J, Li L, Yuen K-Y. Identification of TMPRSS2 as a Susceptibility Gene for Severe 2009 Pandemic A(H1N1) Influenza and A(H7N9) Influenza. J Infect Dis. 2015 Oct 15;212(8):1214–1221. PMID: 25904605

29. Hatesuer B, Bertram S, Mehnert N, Bahgat MM, Nelson PS, Pöhlmann S, Pöhlman S, Schughart K. Tmprss2 is essential for influenza H1N1 virus pathogenesis in mice. PLoS Pathog. 2013;9(12):e1003774. PMCID: PMC3857797

30. Tarnow C, Engels G, Arendt A, Schwalm F, Sediri H, Preuss A, Nelson PS, Garten W, Klenk H-D, Gabriel G, Böttcher-Friebertshäuser E. TMPRSS2 is a host factor that is essential for pneumotropism and pathogenicity of H7N9 influenza A virus in mice. J Virol. 2014 May;88(9):4744–4751. PMCID: PMC3993819

31. Channappanavar R, Fett C, Mack M, Ten Eyck PP, Meyerholz DK, Perlman S. Sex-Based Differences in Susceptibility to Severe Acute Respiratory Syndrome Coronavirus Infection. J Immunol Baltim Md 1950. 2017 15;198(10):4046–4053. PMCID: PMC5450662

32. Cao B, Wang Y, Wen D, Liu W, Wang J, Fan G, Ruan L, Song B, Cai Y, Wei M, Li X, Xia J, Chen N, Xiang J, Yu T, Bai T, Xie X, Zhang L, Li C, Yuan Y, Chen H, Li H, Huang H, Tu S, Gong F, Liu Y, Wei Y, Dong C, Zhou F, Gu X, Xu J, Liu Z, Zhang Y, Li H, Shang L, Wang K, Li K, Zhou X, Dong X, Qu Z, Lu S, Hu X, Ruan S, Luo S, Wu J, Peng L, Cheng F, Pan L, Zou J, Jia C, Wang J, Liu X, Wang S, Wu X, Ge Q, He J, Zhan H, Qiu F, Guo L, Huang C, Jaki T, Hayden FG, Horby PW, Zhang D, Wang C. A Trial of Lopinavir-Ritonavir in Adults Hospitalized with Severe Covid-19. N Engl J Med. 2020 Mar 18. doi: 10.1056/NEJMoa2001282. [Epub ahead of print] PMID: 32187464

33. Ray HE and Rai SN. An evaluation of a Simon 2-Stage phase II clinical trial design incorporating toxicity monitoring. Contemporary Clinical Trials 2011 32; 428–436

16. APPENDICES

APPENDIX A: 7-category Ordinal Scale of Clinical Status of Hospitalized Influenza Patients

1: Not hospitalized with resumption of normal activities.

2: Not hospitalized, but unable to resume normal activities,

3: Hospitalization, not requiring supplemental oxygen.

4: Hospitalization, requiring supplemental oxygen.

5: Hospitalization, requiring nasal high-flow oxygen therapy and/or noninvasive mechanical ventilation.

6: Hospitalization, requiring extracorporeal membrane oxygenation and/or invasive mechanical ventilation.

7: Death.

APPENDIX B: Prohibited agents/treatments that can affect androgen, estrogen and progestin signaling

Androgen receptor antagonists
- Flutamide
- Bicalutamide
- Nilutamide
- Apalutamide
- Darolutamide
- Enzalutamide

LHRH antagonists
- Degarelix
- Abarelix
- Cetrorelix

LHRH agonists
- Leuprolide
- Goserelin
- Triptorelin
- Historelin

CYP17 inhibitors
- Abiraterone
- Ketoconazole

-

Estrogenic agents
- Oral contraceptives
- Post-menopausal hormone replacement therapy

Progestins
- Cyproterone acetate
- Medroxyprogesterone acetate
- Progesterone

Androgens
- Testosterone (enanthate, cypionate and propionate)
- Testosterone undecanoate
- DHT

Aromatase inhibitors
- Formestane
- Exemestane
- Anastrozole

- Letrozole

Miscellaneous
- Spironolactone
- Cimetidine
- Genistein
- Orchiectomy

More info: https://www.cancer.org/cancer/prostate-cancer/treating/hormone-therapy.html

APPENDIX C: CTCAE v5.0

https://ctep.cancer.gov/protocoldevelopment/electronic_applications/docs/CTCAE_v5_Quick_Reference_5x7.pdf

Degarelix Prescribing Information (see attached separate document).

APPENDIX D: Class IA and III Antiarrhythmic Agents

Class IA

Quinidine
Disopyramide
Procainamide
Ajmaline

Class III

Ambasilide
Amiodarone
Dronedarone
Dofetilide
Ibutilide
Sotalol
Vernakalant
Nicorandil
Pinacidil

Overview of Changes from V1.0 to V4.0		
**Version 1.0 (4/7/2020)**	**Version 2.0**	**Section, Page #**
4/20/2020 changes	Updates to contact information	Study Team Roster (page 3/82 v 4.0 track changes all markup)
	Updated Primary, Secondary and Exploratory Outcomes<br>• Primary Outcome changes<br>○ Added "a reduction in mortality, ongoing need for hospitalization, or requirement for mechanical ventilation/extracorporeal membrane oxygenation (ECMO)"<br>○ removed influenza scale or time to discharge<br>• Secondary Outcome changes<br>○ Added "determine if temporary androgen suppression induced by degarelix reduces time to clinical improvement, inpatient mortality, length of hospitalization, duration of intubation for mechanical ventilation, time to achieve a normal temperature, or the maximum severity of COVID-19 illness."<br>○ Removed mortality, the duration of hospitalization, the duration of intubation for mechanical ventilation, the time to achieve a normal temperature, and maximum severity COVID_19 illness<br>○ Added "Determine if degarelix + BSC as compared to placebo + BSC reduces time to clinical improvement as defined by a decline of 2 categories or more from the baseline on the modified 7-category ordinal scale of clinical status of hospitalized influenza patients (influenza scale, see Appendix A)."<br>• Updated Exploratory Outcomes<br>○ Added Determine if age, use of angiotensin converting enzyme inhibitors, the duration of pre-hospitalization symptoms, or the presence or absence of hypertension, cardiovascular disease, asthma, diabetes mellitus, or COPD are prognostically associate with clinical outcome defined by the primary endpoint or any secondary endpoint.<br>○ Added Determine if baseline, Day 8 or Day 15 values for D-dimer, IL-6, LDH, ferritin, total WBC, absolute neutrophil count (ANC), absolute lymphocyte count (ALC), or testosterone are predictive of clinical outcome defined by the primary endpoint or any secondary endpoint.<br>○ Added Determine if degarelix + BSC as compared to placebo + BSC reduces	Summary – Objectives (page 11-13/82 v 4.0 track changes all markup) and Section 2. Study objectives and Endpoints (page 24-27/82 v 4.0 track changes all markup)

	nasopharyngeal (NP) epithelial or peripheral blood mononuclear cell (PBMC) TMPRSS2 expression, NP and plasma viral load, or serum cytokine (TNF[illegible], IL-1[illegible], IL-6) levels.<br>o Added Determine whether germline genomic factors are predictive of clinical outcome defined by the primary endpoint or any secondary endpoint. This analysis is optional and is contingent on obtaining adequate number of samples.<br>o Added To determine the temporal onset of anti-viral antibodies."	
	Added Expected Treatment Effects	Summary – (page 14/82 v 4.0 track changes all markup), Section 4. Study Enrollment Procedures (page 37-38/82 v 4.0 track changes all markup) and Section 10. Statistical Considerations - Sample Size Calculation and Power Analysis (page 54/82 v 4.0 track changes all markup
	Updated Sample Size Calculation and Power Analysis	
	Deleted Duration of Study and Number of Participating Sites	
	Updated Study Schematic	
	Updated inclusion / Exclusion Criteria<br>• Updated Inclusion (patients > 85 can be enrolled if there is no history of chronic obstructive pulmonary disease (COPD), asthma, cardiovascular disease, hypertension, diabetes mellitus or active malignancy)<br>• Added Exclusion for systemic glucocorticoids, hydroxychloroquine, chloroquine, or azithromycin within 30 days of day of study drug administration.	Summary - Eligibility Criteria (page 16-17/82 v 4.0 track changes all markup) and Section 3. Study Population - Eligibility Criteria (page 28-30/82 v 4.0 track changes all markup)
	Updated Glossary	Glossary (page 18/82 v 4.0 track changes all markup)
	Updated Study Design Overview with new Primary Endpoint<br>• Added "The primary endpoint is to determine if degarelix + best supportive care (BSC) as compared to placebo + BSC reduces the composite endpoint of mortality, ongoing need for hospitalization, or requirement for mechanical ventilation/extracorporeal membrane oxygenation (ECMO) at Day 15 after randomization."<br>• Deleted "The primary endpoint is the time to clinical improvement as defined by a decline of two categories or more from the baseline on the modified seven category ordinal scale of clinical status of hospitalized influenzas patients (influenza scale, Appendix A) or hospital discharge, whichever comes first."	Section 6. Study Design – Study Design Overview (page 34/82 v 4.0 track changes all markup)

	Added stratifation factors<br>• Age <65 vs. >65<br>• History of Hypertension: yes vs. no<br>Influenzas severity scale: 3 vs. 4/5	Section 4. Study Enrollment Procedures - Randomization Procedures (page 36/82 v 4.0 track changes)
	Study Schema added	Section 5. Study Design - Study Schema (page 37/82 v 4.0 track changes all markup)
	Updated Duration of Patient Follow-up<br>• Deleted "if patient discharged prior to day 30, patients will be followed by remote telephone contact every 7+/3 days for vital status (i.e. death) until Day 30"	Section 5. Study Design - Duration of Patient Follow-up (page 38/82 v 4.0 track changes all markup)
	Clarified Documentation requirement for Sixty Minute Rule replacement	Section 6. Study Treatment - Sixty Minute Rule (page 41/82 v 4.0 track changes all markup)
	Updates to Source and Handling of Study Drug<br>• Including study medication and supplies accountability, Receipt and Dispensing records, Special handling (NIOSH group 1),	Section 6. Study Treatment - Source and Handling of Study Drug (page 42-44/82 v 4.0 track changes all markup)
	Updates to Schedule of Events Table 1<br>• Added - Collection of research blood and NP swabs will be attempted at the indicated time points, but it is understood that some of these samples may not be obtained due to logistical reasons, such as safety. Accordingly, the failure to collect the research blood or NP swabs will not be considered a protocol deviation.<br>• Laboratory abnormalities observed during study enrollment will be managed according to local hospital practices and good standard of care. Study teams will focus on electrolyte abnormalities that could impact risk for cardiac events.	Section 7. Study Procedure – Table 1. Schedule of Events (page 46-47/82 v 4.0 track changes)
	Added Handling of Research Specimens<br>• The research specimens will be transported to UCLA for analysis on the same day of collection whenever possible. If samples cannot be transported to UCLA on the same day of collection, then the samples will be stored within the GLA Medical Center Building 500 Clinical Research Center. In the Clinical Research Center, the NP swabs will be stored at room temperature, and blood specimens will be stored at -20°C.	Section 7. Study Procedure – Handling of Research Specimens (page 48/82 v 4.0 track changes)

	Updated Adverse event severity scale	Section 8. Adverse Events and Serious Adverse Events – Severity of an Adverse event (page 50/82 v 4.0 track changes)
	Added Expedited Reporting of Adverse Events of Special Interest<br>• Cardiac arrhythmias and thromboembolic events will be considered AEs of special interest. Any cardiac arrhythmia or thromboembolic event of grade 3-5 according to Common Terminology Criteria for Adverse Events version 5.0 (CTCAE v5.0; see Appendix C) will be reported in a manner similar to SAEs, whereby the event will be reported to the HITCH reporting system within 3 calendar days of the Study Site Personnel becoming aware of the event	Section 8. Adverse Events and Serious Adverse Events – Severity of an Adverse event (page 52/82 v 4.0 track changes)
	DMA frequency updated<br>• From every 4 months to monthly	Section 9. Safety Monitoring – Data and Safety Monitoring Plan (page 53/82 v 4.0 track changes)
	Added Additional Safety Monitoring for AEs of Special Interest<br>• Whereas the DMC serves as the entity with primary oversight responsibility for the study, additional real-time monitoring will be conducted to capture AEs of special interest, including cardiac arrhythmias and thromboembolic events that may result from degarelix. In particular, cardiac arrhythmias and thromboembolic complications of grades 3-5 according to CTCAE v5.0 (see Appendix C) will be closely monitored through not only ongoing, daily review of AE and SAE reports, but also through regular, planned teleconference calls to include: The study chair (Dr. Rettig), co-chair (Dr. Nickols), and the lead site coordinator (Ms. Samantha Tran),<br>○ One or more investigators from each site and/or one or more site coordinators.<br>○ The teleconferences will occur on Monday, Wednesday and Friday of each week until the LPI + 30 days. In addition, study sites should report the onset arrhythmias of thromboembolic events of grade 3-5 according to CTCAE v5.0 within 72 hours by contacting the study chair or co-chair by email and voice call (see page 5 for contact information).	Section 9. Safety Monitoring Additional Safety Monitoring for AEs of Special Interest (page 53/82 v 4.0 track changes)
	Updated to Statistical Considerations<br>• Added to Expected Treatment Effects	Section 10. Statistical Considerations - Sample Size Calculation and Power

	• Figure 1: Interim analysis stopping boundaries for efficacy and futility<br>• Table 2: Sample Size Estimation and Power Analysis<br>• Interim Analysis and Criteria for Study Termination<br>• Data Analysis Plan<br>• Table 3: Summary of Data Analysis Plan	Analysis (page 54-71/82 v 4.0 track changes all markup)
	Removed Appendix C: ECOG Performance Status	(page 82/82 v 4.0 track changes all markup)
Submitted Protocol Version 2.0 (4/23/2020) to the FDA for Exemption Determination Request		
4/23/2020	Updates to sample size and power analysis<br>• 40% changed to 42%<br>• 85% power changed to 90% power<br>• 162 evaluable patients changed to 186 evaluable patients<br>○ 108 to 124 active, 57 to 62 placebo<br>• 171 patients required changed to 198<br>○ 114 to 132 active, 57 to 66 placebo	Sample Size Calculation and Power Analysis summary and Section 10. Statistical Considerations - Sample Size Calculation and Power Analysis (page 54/82 v 4.0 track changes all markup)
	Clarified timeframe for administration of study medication from randomization<br>• within 24 hours of randomization (i.e. within 24 hours of	Study Treatments summary<br>Section 6. Study Treatment – Source and Handling of Study Drug and Storage and Handling Requirements (page 42-44/82 v 4.0 track changes all markup)
	Clarified exploratory endpoint, germline genomic factors, as optional	Section 2. Study objectives and Endpoints - Exploratory endpoints (page 24/82 v 4.0 track changes all markup)
	Added exclusion<br>• Patients who have been hospitalized for COVID-19 for more than 72 hours, including the hospitalization time at another hospital for patients who were transferred.	Section 3. Study Population - Eligibility Criteria (page 28-30/82 v 4.0 track changes all markup)
	Updated study schema	Section 5. Study Design - Study Schema (page 37/82 v 4.0 track changes all markup)

	Updated sample size<br>• 171 patients required changed to 198<br>○ 114 to 132 active, 57 to 66 placebo	Section 5. Study Design – Sample Size (page 37/82 v 4.0 track changes all markup)
	Updates to Table 2: Sample Size Estimation and Power Analysis	Section 10. Statistical Considerations - Sample Size Calculation and Power Analysis (page 54-55/82 v 4.0 track changes all markup)
	Updates to Interim Analysis and Criteria for Study Termination<br>• 86 changed to 99 randomized patients complete<br>• Boundary values adjusted from 2.75 to 2.77 and 0.59 to 44 respectively<br>• Toxicity markers from 33% to 25% and alpha from 0.05 to 0.01	Section 10. Statistical Considerations - Interim Analysis and Criteria for Study Termination (page 56/82 v 4.0 track changes all markup)
	Updates to Figure 1: Interim analysis stopping boundaries for efficacy and futility<br>• boundary plot	Section 10. Statistical Considerations - Figure 1: Interim analysis stopping boundaries for efficacy and futility (page 57/82 v 4.0 track changes all markup)
	Updates to testing power<br>• 85% power changed to 90% power	Section 10. Statistical Considerations - Data Analysis Plan – Analysis population
4/27/2020	Removed reference to obtaining HIPAA authorization<br>• The study was granted a waiver of HIPAA Authorization for the entire study, revised protocol to be consistent	Section 4. Study Enrollment Procedures - Informed Consent (page 31-32/82 v 4.0 track changes) and Section12. Patient Rights and Confidentiality – Patient Confidentiality (page 74/82 v4.0 track changes
**Version 2.0**	**Version 3.0**	
5/5/2020 changes reflect CIRB amendment - updates	Added definition of “discharge” as being no longer requiring hospitalization for COVID-19 in the endpoints to protocol<br>• Patients could be discharged to home, rehab center or nursing facility; wanted to capture all scenarios	Section 2. Study Objectives and Endpoints (page 26/82 v 4.0 track changes) and Section 11. Data Collection and Quality Assurance –

identified during Kick-off training		Data Collection Forms (page 74/82 v 4.0 track changes)
	Added EKG will be needed 60 days (-60 days from Day 1) prior to drug administration to protocol<br>• One of the exclusion criteria is history of congenital long QT syndrome of prolong QT interval. An EKG is necessary to determine that exclusion criteria and if one has not been done for standard of care prior to drug administration, patient will undergo one strictly for research purposes	Exclusion criteria (page 16-17/82 and pages 28-29/82 v 4.0 track changes)
	Added "remdesivir at discretion of treating physician" as being an exception to anti-viral treatments in the exclusion criteria to protocol<br>• After the study received initial approval, the FDA approved remdesivir under an Emergency Use Authorization (EUA) for the treatment of COVID-19	
	Added the names and contact information of the CSP staff to the protocol<br>• Wanted the protocol to identify all those involved in the study	Study Team Roster (page 5/82 v 4.0 track changes)
	Added information about EKG and associated risks<br>• Added the possibility of EKG test if one is not already done within 60 days of randomization to ICF and assent form	Exclusion criteria (page 16-17/82 and pages 28-29/82 v 4.0 track changes)
**Version 3.0**	**Version 4.0**	
Changes include items requested and recommended by FDA (PIND: 150380 Meeting Request - Written Responses - Ref:4612064)	15 day time point based on placebo arm from 2 RCT, remdesivir and Lopinivir/rotinivir studies	Synopsis – Endpoints (page 11/82 v 4.0 track changes)
	reconcile the hydroxychloroquine or chloroquine use discrepancy:<br>• removed "Use of hydroxychloroquine or chloroquine is permitted" (page 46/82 v 4.0 track changes)	Exclusion criteria (page 16-17/82 and pages 28-30/82 v 4.0 track changes)
	Added use of drugs known to increase QT	
	Added baseline electrolyte abnormalities of grade 3 or higher	

	Added MI in the past 6 month, severe angina or unstable angina or NYHA class III or IV heart disease	
	Added Child-Pugh class C liver disease. Note added to justify Child-Pugh class A and B	
	Updated the prohibition of concomitant medication to include:<br>• hydroxychloroquine and chloroquine,<br>• drugs known to prolong QT interval	Section 6. Study Treatment – Prohibited Concurrent Treatment and Medications ” (page 46/82 v 4.0 track changes)
	Added Justification /rational for LHRH drug choice and dose in study population. As well as expected CV risks	Selection and description of study drug (page 24/82 v 4.0 track changes)
	Study population justification for younger patients (<40 yrs)	Study Population – Rational for Patient Selection (page 27/82 v 4.0 track changes)
	Added use of unblinded administrator to account for active drug vs placebo formation differences and risk for unblinding	Section 4. Study Enrollment Procedures - Blinding (page 33/82 v 4.0 track changes)
	Clarified follow-up for patients who remain hospitalized beyond 30 days	Section 5. Study Design – Duration of Patient Follow-up (page 38/82 v 4.0 track changes)
	Added Justification /rational for degarelix 240 mg dose for study endpoint<br>• Clinical safety experience with degarelix	Section 6. Study Treatment (page 38/82 v 4.0 track changes)
	Removed language describing rational for FDA exemption	Section 6. Study Treatment (page 38/82 v 4.0 track changes)
	Added expected risks/ benefits associated with one time use of degarelix 240 mg dose for study population to include anticipated labeled and potential safety concerns of degarelix administration in the COVID-19 population based on one time dose of degarelix 240 mg.	Section 6. Study Treatment (page 39-40/82 v 4.0 track changes)
	Clarified Best Supportive Care (BSC) will be adaptive as novel treatments emerge<br>• address anticipated off-label use of any other drugs, devices, or interventions that might be used to manage COVID-19<br>• DUR prior to use to identify overlapping toxicities	Section 6. Study Treatment (page 40/82 v 4.0 track changes)

	Added daily lab collections and ECG<br>• CBC with differential, chem panel and electrolytes, coagulation tests, cardiac blood tests, inflammatory tests, and LFTs - at screening and daily<br>o "Chem panel = Sodium, potassium, chloride, CO_2, blood urea nitrogen, creatinine. Electrolytes = Calcium, magnesium, phosphorus. LFTs = AST, ALT, albumin, total bilirubin, alkaline phosphatase, LDH. Cardiac blood tests = troponin, CPK. Coagulation tests = D-dimer, PT/INR and aPTT. Inflammatory blood tests = CRP, IL-6, ferritin and pro-calcitonin. Some of these labs are collected for the purposes of safety assessments and risk mitigation and will not necessarily be collected in CRFs."<br>• EKG.<br>o EKGs beyond the screening window will be performed per standard of care.	Section 7. Study Procedure – Table 1. Schedule of Events (page 47-48/82 v 4.0 track changes)
	Clarified Adverse events will be graded according to CTCAE v5.0	Section 8. Adverse Events and Serious Adverse Events – Severity of an Adverse event (page 50/82 v 4.0 track changes)
	Included language "FDA should be notified (1) for any subject who is withdrawn for safety reasons and (2) for any DMC decision to pause enrollment or terminate the study".	Section 8. Adverse Events and Serious Adverse Events – Reporting to FDA (page 52-53/82 v 4.0 track changes)
	Added enrollment pause from time of analysis until final DMC recommendations	Section 10. Statistical Considerations – Interim analysis and Criteria for Study termination (page 56/82 v 4.0 track changes)
	Added a stratified Cochran Mantel-Haenszel method to adjust for three stratification factors as one of the sensitivity analyses for the primary efficacy endpoint.	Section 10. Statistical Considerations – Data Analysis plan (page 59/82 v 4.0 track changes)
**Version 4.0**	**Version 5.1**	**Section, Page #**
Changes include items recommended by FDA in the "Study may	Increased time to secondary endpoint from 15 days to 30 days<br>Corrected discrepancy	Endpoints (page 12/68 and pages 25-22-23/68 v5.1 track changes)

Proceed" letter (Reference ID: 4625728)	• reducing time to clinical improvement or hospital discharge whichever comes first	
	Updated exclusions<br>• EKG requirement<br>• Medication exceptions<br>• Electrolyte abnormalities<br>• Heart disease<br>• Liver disease	Exclusion criteria (page 15-16/68 pages 25-26/68 v5.1 track changes)
	Updated study schema diagram and duration to include the extended follow up<br>• Assessment of response and additional follow-up for primary endpoint = date of LPI + estimated – from 30 days to 60 days.<br>• Total study duration = estimated from 150 days to 210 days.	Section 5. Study Design – Study Duration (page 32/68 v5.1 track changes)
	Clarified follow-up for patients<br>• Until death or 60 days after randomization	Section 5. Study Design – Duration of Patient Follow-up (page 32/68 v5.1 track changes)
	Updates to Best Supportive Care	Section 5. Study Design – (page 33-34/68 v5.1 track changes)
	Added use of unblinded administrator to account for active drug vs placebo formation differences and risk for unblinding<br>Clarified responsibilities of the Local Research Pharmacist	Section 6. Study Treatment – Prescribing and dispensing Study Products (page 35/68 v 5.1 track changes)
	Added "bruising" sat injection site	Section 6. Study Treatment – Adverse reactions (page 39/68 v 5.1 track changes)
	Updated the prohibition of concomitant medication to include:<br>• Dexamethasone was recently reported to reduce COVID-19 severity, so glucocorticoids for the purpose of treating COVID-19 are permitted.	Section 6. Study Treatment – Prohibited Concurrent Treatment

	• Use of hydroxychloroquine and chloroquine are prohibited<br>• Use of drugs known to prolong QT interval are prohibited	and Medications ” (page 39/68 v 5.1 track changes)
	Updated to schedule of events table<br>• Daily lab collection will include chem panel and electrolytes, coagulation tests, cardiac blood tests, inflammatory tests, and LFTs<br>• EKGs beyond the screening window will be performed per standard of care.<br>• Upon discharge, patients will be followed for vital status, need for re-hospitalization, and adverse events.<br>• Laboratory abnormalities observed during study enrollment will be managed according to local hospital practices and good standard of care. Study teams will focus on electrolyte abnormalities that could impact risk for cardiac events.	Section 7. Study Procedure – Handling of Research Specimens (page 40-41/68 v5.1 track changes)
	Updated timeframe for reporting<br>• LPI+30 days changed to LPI+60 days	Section 8. Adverse Events and Serious Adverse Events – Reporting to FDA (page 47/68 v 5.1 track changes)
	Added<br>• Enrollment will be paused if the interim analysis results indicate crossing of the threshold for futility or efficacy until the final DMC recommendation is rendered.	Section 10. Statistical Considerations - Interim Analysis and Criteria for Study Termination (page 48/68 v 5.1 track changes all markup)
	Added<br>• Further, Cochran Mantel-Haenszel method will be used to adjust for three randomization stratification factors (Age: <65 vs. ≥ 65, History of hypertension: yes vs. no, and Influenza severity scale: 3 vs 4/5) using SAS PROC FREQ as one of the sensitivity analyses for the primary efficacy endpoint.<br>• Updated α-level from 0.0083 to 0.0071 for each endpoint.<br>• The composite outcome of mortality, ongoing need for hospitalization, and ECMO or mechanical intubation at 30 days after randomization as a secondary endpoint will be analyzed using the methods described in the primary endpoint analysis.	Section 10. Statistical Considerations - Primary and Secondary Endpoint Analysis (page 50/68 v 5.1 track changes all markup)

	Updated Table 3 Summary of Data Analysis Plan	Section 10. Statistical Considerations – Table 3 Summary of Data Analysis Plan (page 56/68 v 5.1 track changes all markup)
**Version 5.1**	**Version 5.2**	
Changes include removing use of azithromycin as an exclusion criteria and added contact information for other sites that will be part of the trial	Added other participating sites and LSI contact information	"Study Team Roster" (p. 8 - 10)
	Removed azithromycin as an exclusion criteria	"Synopsis" section, under "Exclusion" subsection (p. 17) and "Exclusion" section (p. 27)
**Version 5.2**	**Version 5.3**	**Section, Page #**
	Removed – unblinded administrator will prepare the study medication	Preparing and dispensing study products (p. 36)
	Added Day 60, added AE requirement at day 60, added post discharge follow up at day 30 and day 60.<br>Added footnote (b) "assessment should continue as patient is hospitalized<br>Added footnote (d) "the collection of labs will discontinue when the participant is discharged"	Study procedures, Schedule of Events (p.41)
	Updated 30 days to 60 days	Overview of Adverse Event Reporting (p.42)
	Updated 29 to 30	Data Collection Forms (p. 57)
	Removed 5 alpha reductase inhibitors, dutasteride, finasteride	Appendix B (pg. 66)
	Added Greg Orshansky, MD as a co-investigator	Pg. 6

	Change exclusion criteria of "Use of any drug known to prolong QT intervals within 30 days of Day 1" to "Chronic use of any drug known to prolong QT intervals within 30 days of Day 1. One time use will be acceptable as long as there is sufficient time between drug administration and initiation of the study drug. This will be determined on a case of case basis under the discretion of the PI and/or co-investigators".	Pg. 17 & 28
	Protocol "Study Procedures", Schedule of Events table (page 41) – removed IL-6 and ferritin from the list of study assessments. IL-6 and ferritin are test that are ordered for inflammatory blood tests and this is collected daily, so it was removed to prevent duplicate testing	Schedule of Events table (p.41)

**Protocol Version**	**Changes made**
Version 5.3, August 04, 2020	**Study Team Roster:** added Greg Orshansky, MD, FACP as a co-investigator at GLA<br><br>**Synopsis and Section 3 - Exclusion Criteria:** revise exclusion #8 to allow use of any drug known to prolong QT intervals within 30 days of Day 1. One-time use will be acceptable as long as there is sufficient time between drug administration and initiation of the study drug. This will be determined on a case by case basis under the discretion of the PI and/or co-investigators. An electrocardiogram will be performed to ensure a normal QTc value (QT interval corrected by the Fridericia correction formula (QTcF) < 500))<br><br>**Section 6. Study Treatment - Prescribing and Dispensing Study Products:**<br>• removed the following statement, "The unblinded administrator will prepare the study medications, reconstitution of degarelix or saline, and affix the study drug label to each syringe prior to entering the room of the participant"<br><br>• revised the baseline collection of blood and nasal swab out of Day 1 to up to 72 hours from Day 1<br>• revised to include post discharge follow-up assessments to the Schedule of Events table and added Column for Day 60<br>• removed IL-6 and ferritin from the list of study assessments as these tests are already part of the labs ordered for inflammatory blood tests (prevent duplicate testing/labs)

	**Section 8. Adverse Events and Serious Adverse Events:** revised that AEs and SAEs will be collected until 60 days after drug administration<br><br>**Section 16. Appendices - Appendix B:** removed Dutasteride and Finasteride (5 alpha reductase inhibitors) off the prohibited treatments as they do not reduce serum total testosterone and is not medically necessary to exclude patients on these drugs
Version 5.4, November 3, 2020	**Synopsis and Section 3 – Exclusion Criteria:** removed exclusion #2 that excludes active use of anti-viral therapies directed at SARS-CoV-2, except for remdesivir at the discretion of the treating physician.<br><br>**RATIONALE:** The exclusion criterion that relates to concurrent anti-viral antibiotics and other antibiotics is no longer relevant to the study. There are no anti-virals or anti-infective agents that are exclusionary. As a consequence, this exclusion criterion only serves to create confusion around the determination of patient eligibility.<br><br>**Synopsis and Section 3 – Exclusion Criteria:** removed exclusion #6 that excludes any concurrent use of systemic glucocorticoids within 30 days of study drug administration, except when used for the treatment of COVID-19 (e.g. dexamethasone).<br><br>**RATIONALE:** Adrenal androgens contribute only about 5% to the total circulating androgen pool. That is, only 5% of androgens produced in an adult male are derived from the adrenal gland. Glucocorticoids can partially suppress adrenal androgen production, but the net effect on the total circulating androgen levels is negligible. Given this information and the fact that many COVID-19 patients are on glucocorticoids for the treatment of COVID-19 as well as for other indications, the study is eliminating the exclusion criterion related to concurrent use of glucocorticoids.<br>**Synopsis and Section 3 – Exclusion Criteria:** removed exclusion #7 that excluded the use of hydroxychloroquine and chloroquine within 30 days of Day 1<br>**RATIONALE:** The exclusion criterion related to concurrent use of hydroxychloroquine or chloroquine is no longer relevant to the study. These agents were originally excluded because there was uncertainty about the efficacy of hydroxychloroquine or chloroquine for the treatment of COVID-19. Now that multiple controlled trials have been reported on this subject and these agents are NOT effective for the treatment of COVID-19, the study is eliminating the exclusion related to hydroxychloroquine or chloroquine.

	**Synopsis and Section 3 – Exclusion Criteria:** revised exclusion #10 to indicate that patients cannot have a myocardial infarction (MI) in the past 6-months instead of 60-months. The time between last MI and study drug administration does not need to be 60-months.<br><br>**Section 11, Patient Discontinuation:** removed 2nd bullet as patients will not be discontinued from study at time of discharge<br><br>• Revised 1st bullet to include that patients will be discontinued from study after death or Day 60 (whichever comes first)
Version 5.4.5, January 15, 2021	**Synopsis and Section 3 – Inclusion Criteria:** revised inclusion criteria #2 to remove age limit of 85 years old to have no age limit.<br><br>**RATIONALE:** in clinical care setting, degarelix is given to patients older than 85 to treat their prostate cancer and the drug package insert does not limit the maximum age<br><br>**Synopsis and Section 3 – Inclusion Criteria:** revised inclusion criteria #3 to clarify that main cause for hospitalization does not have to be COVID-19, only that COVID-19 contributes to hospitalization<br><br>**RATIONALE:** to clarify that even if a patient is hospitalized for another reason but has a positive COVID-19 diagnosis, patient may be eligible for trial if COVID symptoms worsen during hospitalization<br><br>**Synopsis and Section 3 – Exclusion Criteria:** added exclusion criteria of under the age of 18<br><br>**Synopsis and Section 3 – Exclusion Criteria:** added exclusion criteria of severity of COVID-19 illness level of < 3 on the influenza severity scale<br><br>**RATIONALE:** added to clarify exclusion criterion regarding severity of COVID-19 illness<br>**Synopsis and Section 3 – Exclusion Criteria:** added exclusion criteria that explicitly excludes QTcF > 500 msec on screening ECG

	**Synopsis and Section 3 – Exclusion Criteria:** revised exclusion criteria regarding use of drugs known to prolong QT intervals to match the FDA package label for degarelix (Firmagon). Excluded agents are now Class IA and Class III antiarrhythmics (listed in Appendix D)<br><br>**RATIONALE:** Given other exclusion criteria that reduce cardiac risk, including requirements for measurement and correction of electrolyte abnormalities (exclusion criterion 9), the exclusion of patients with history of prolonged QT or prolonged QT on screening ECG (exclusion criteria 4 and 5), and the exclusion of patients with myocardial infarction in the past 6 months, severe or unstable angina, or New York Heart Association (NYHA) Class III or IV heart disease (exclusion criterion 11), our exclusion criteria overall will remain more stringent than that which is recommended in the FDA package label for degarelix.<br><br>**Synopsis and Section 3 – Exclusion Criteria:** revised to clarify use of:<br>• Androgen receptor antagonists or agonists withing 4-weeks of study enrollment<br>• Ketoconazole or abirterone acetate within 2-weeks of study enrollment<br>• Estrogens or progestins within 2-weeks of study enrollment<br>• Any prior use of an LHRH analogue unless a serum total testosterone measured within 30 days of study enrollment is ≥ 150 ng/dL.<br>• Other hormonal agents listed in Appendix B within one day of study enrollment.<br><br>**RATIONALE:** Clarified the exclusion criterion related to prohibited use of hormonal agents.<br><br>**Study Enrollment Procedures:** added a description on the assent and LAR/surrogate consent process and use of an "an honest broker" to help LAR/surrogate get a better understanding of patient's capacity to understand the study<br><br>**Study Procedures, Schedule of Events Table** – revised inflammatory tests (CRP, IL-6, ferritin and pro-calcitonin) to be drawn on Screening and weekly only<br><br>**RATIONALE:** Clarified the frequency of blood tests of inflammation for exploratory analyses from daily to weekly

Protocol 5.5, Date TBD	Removes drugs known to cause prolong QT

<u>Version control dates:</u>

Version 1.0 - April 7, 2020 – submitted to CIRB
Version 2.0, April 27, 2020 – CIRB approved 05/01/2020 under initial PI/SC application 2020 (submitted to FDA) – 4/27/2020
Version 3.0, May 05, 2020 – CIRB approved 05/07/2020 under PI/SC Amendment 01

Version 4.0 - 5/25/2020 – 5/27/2020 (submitted to FDA)– 6/5/2020
Version 5.1, June 18, 2020 – CIRB approved 06/23/2020 under PI/SC Amendment 02

Version 5.2, July 14, 2020 – CIRB approved 07/17/2020 under PI/SC Amendment 04 (submitted to FDA) – 8/3/2020

Version 5.3, August 04, 2020 – CIRB approved 09/14/2020 under PI/SC Amendment 05 (submitted to FDA) – 8/14/2020

Version 5.4, November 03, 2020 – CIRB approved 11/17/2020 under PI/SC Amendment 08 (submitted to FDA) – 11/23/2020

Version 5.4.5, January 15, 2021 – submission under PI/SC Amendment 10

Version 5.5, TBD – submission under PI/SC Amendment 11 (submitted to FDA) – 11/30/2020
