# Supplementary material for: Effect of Androgen Suppression on Clinical Outcomes in Hospitalized Men With COVID-19: The HITCH Randomized Clinical Trial
Source: JAMA Netw Open. 2022 Apr 19;5(4):e227852. doi: 10.1001/jamanetworkopen.2022.7852 (PMC9020208; doi:10.1001/jamanetworkopen.2022.7852)
Supplement: Supplement 2. — eTable 1. Inclusion and Exclusion Criteria eTable 2. Reasons for Study Exclusion eTable 3. Testosterone Levels After Degarelix Treatment eTable 4. Adverse Events for Randomized Participants by Treatment Groups eTable 5. Serious Adverse Events for Randomized Participants by Treatment Groups [file jamanetwopen-e227852-s002.pdf]

Supplemental Online Content

Nickols NG, Mi Z, DeMatt E, et al. Effect of androgen suppression on clinical outcomes in hospitalized men with COVID-19: the HITCH randomized clinical trial. *JAMA Netw Open*. 2022;5(4):e227852. doi:10.1001/jamanetworkopen.2022.7852

eTable 1. Inclusion and Exclusion Criteria

eTable 2. Reasons for Study Exclusion

eTable 3. Testosterone Levels After Degarelix Treatment

eTable 4. Adverse Events for Randomized Participants by Treatment Groups

eTable 5. Serious Adverse Events for Randomized Participants by Treatment Groups

This supplemental material has been provided by the authors to give readers additional information about their work.

© 2022 Nickols NG et al. *JAMA Network Open.*

eTable 1. Inclusion and Exclusion Criteria

Inclusion Criteria	Exclusion Criteria
Male Veterans admitted to a VA hospital.	History of severe hypersensitivity to degarelix or any component of their respective formulation
Age ≥ 18	History of congenital long QT syndrome or known history of prolonged QT interval OR Fridericia correction formula (QTcF) > 500 msec on electrocardiogram performed at screening
Hospitalized on an acute care ward with a diagnosis of COVID-19 contributing to hospitalization.	Planned discharge within 24 hours of treatment initiation
Positive RT-PCR assay for SARS-CoV-2 on a nasopharyngeal swab sample.	Subject is planning to conceive or father children within the projected duration of the study, starting with the screening visit through 120 days after the last dose of study treatment.
Severity of illness of level 3, 4 or 5 on the influenza severity scale (hospitalized but not requiring invasive mechanical ventilation) at the time of randomization.	Ongoing usage of a Class IA or Class III antiarrhythmic agent (see Appendix D). At least 5 half-lives must elapse since any prior use of a Class IA or III antiarrhythmic agent prior to administration of study drug
The subject (or legally acceptable representative if applicable) must provide informed consent for the trial.	Baseline electrolyte abnormalities of Grade 3 or higher (based on CTCAE v5.0 criteria). Patients may be included if baseline electrolyte abnormalities are corrected to Grade 2 or lower prior to study drug administration
	Myocardial infarction in the past 6 months, severe or unstable angina, or New York Heart Association (NYHA) Class III or IV heart disease
	Enrollment in another investigational study within 30 days of Day 1
	Known psychiatric or substance abuse disorder that would interfere with the requirements of the trial
	Child-Pugh Class C liver disease.
	Use of any of the following hormonal agents within Day 1 of treatment:<br><br>Androgen receptor antagonists or agonists within 4 weeks, Ketoconazole or abiraterone acetate within 2 weeks, Estrogens or progestins within 2 weeks, Herbal products that contain hormonally active agents within 2 weeks, Any prior use of an LHRH analogue unless a serum total testosterone measured within 30 days of study enrollment is ≥ 150 ng/dL

© 2022 Nickols NG et al. *JAMA Network Open.*

	Unwilling or unable to comply with the study protocol.
	Any condition, which in the opinion of the Investigator, would preclude participation in the trial

© 2022 Nickols NG et al. *JAMA Network Open.*

eTable 2. Reasons for Study Exclusion

	Total
**Screened**	**2154**
Excluded Prior to Consent	**2024**
Consented	**130**
Excluded After Consent	**29**
Declined	**5**
Randomized	**96**
**Reasons for Exclusion:**	
Unwilling or unable to provide informed consent	**632**
Not a veteran	**2**
Not male	**34**
Age < 18 or age > 85 with history of COPD, asthma, cardiovascular disease, hypertension, diabetes mellitus or active malignancy	**157**
Not hospitalized on an acute care ward with a diagnosis of Covid-19 contributing to hospitalization	**408**
No positive RT-PCR assay for SARS-CoV-2 on a nasopharyngeal swab sample	**182**
Severity of COVID-19 illness level not a 3, 4, or 5 on the 7-Category Ordinal Scale of Clinical Status of Hospitalized Influenza Patients	**275**
History of severe hypersensitivity to degarelix or any component of its respective formulation	**10**
Actively using anti-viral therapies directed at SARS-CoV-2, except remdesivir at the direction of the treating physician	**8**
History of congenital long QT syndrome or known history of prolonged QT interval or Fridericia correction formula (QTcF) > 500 msec on electrocardiogram pe	**137**
Planned discharge within 24 hours of treatment initiation	**383**
Planning to conceive or father children within the projected duration of the study	**5**
Concurrent use of systemic glucocorticoids within the past 30 days, except when used for the treatment of COVID-19	**47**
Use of hydroxychloroquine or chloroquine within the past 30 days	**3**
Chronic use of a drug known to prolong QT interval within the past 30 days	**467**
Ongoing use of a Class IA or Class III antiarrhythmic agent	**9**
Baseline electrolyte abnormalities of Grade 3 or higher (based on CTCAE v5.0 criteria)	**90**

© 2022 Nickols NG et al. *JAMA Network Open.*

Enrolled in another investigational study within the past 30 days	**50**
Diagnosed with a myocardial infarction in the past 6 months, severe or unstable angina, or NYHA Class III or IV heart disease	**153**
Psychiatric or substance abuse disorder that would interfere with the requirements of the trial	**220**
Diagnosed with Child-Pugh Class C liver disease	**13**
Use of androgen receptor antagonists or agonists in the past 4 weeks	**73**
Use of ketoconazole or abiraterone acetate in the past 2 weeks	**8**
Use of estrogens or progestins in the past 2 weeks	**3**
Use of herbal products that contain hormonally active agents in the past 2 weeks	**4**
Any prior use of an LHRH analogue unless a serum total testosterone measured within 30 days of study enrollment is greater than or equal to 150 ng/dL	**2**
Use of other hormonal agents listing in Appendix B of the protocol in the past 1 day	**3**
Unwilling or unable to comply with the study protocol	**268**
Has a condition, which in the opinion of the investigator, would preclude participation in the trial	**457**
**Total**	**4103**
**Note: Can have multiple reasons for exclusion per participant.*	

© 2022 Nickols NG et al. *JAMA Network Open.*

eTable 3. Testosterone Levels After Degarelix Treatment

	Statistics	Degarelix (N=62)	Placebo (N=34)	Total (N=96)	P-value
Baseline	n	51	27	78	0.648
	Mean (SD)	165.3 (174.7)	148.6 (98.33)	159.5 (152.1)	
	LS Mean (SE)	165.3 (21.41)	148.6 (29.43)	157.0 (18.20)	
	Median	111.0	133.0	120.5	
	Min, Max	1, 913	0, 399	0, 913	
Day 8	n	28	10	38	0.007
	Mean (SD)	40.3 (74.23)	87.2 (96.56)	52.7 (82.02)	
	LS Mean (SE)	40.4 (13.18)	119.6 (23.36)	80.0 (13.41)	
	Median	15.0	31.5	16.0	
	Min, Max	0, 348	9, 268	0, 348	
Day 15	n	9	4	13	0.241
	Mean (SD)	52.1 (90.24)	67.0 (106.2)	56.7 (91.11)	
	LS Mean (SE)	24.5 (30.14)	87.8 (37.39)	56.1 (23.27)	
	Median	5.0	21.5	9.0	
	Min, Max	0, 239	0, 225	0, 239	
Day 30	n	2	1	3	.
	Mean (SD)	153.0 (117.4)	0.0	102.0 (121.2)	
	LS Mean (SE)		0.0		
	Median	153.0	0.0	70.0	
	Min, Max	70, 236	0, 0	0, 236	

LS Means presented at Day 8, Day 15 and Day 30 are adjusted for Baseline
Overall P-value is 0.003 for the treatment from mixed model with week and treatment interaction and adjusted for the baseline.

© 2022 Nickols NG et al. *JAMA Network Open.*

eTable 4. Adverse Events for Randomized Participants by Treatment Groups

Body System and Preferred Term	Degarelix (N= 62)			Placebo (N= 34)			Total (N= 96)			P-value
	N	%	Events	N	%	Events	N	%	Events	
**All Adverse Events**	**13**	**21**	**19**	**8**	**23.5**	**12**	**21**	**21.9**	**31**	**0.772**
**Cardiac disorders**	**2**	**3.2**	**2**	**3**	**8.8**	**3**	**5**	**5.2**	**5**	**0.343**
Atrial fibrillation	1	1.6	1	2	5.9	2	3	3.1	3	0.285
Accelerated idioventricular rhythm	0	0	0	1	2.9	1	1	1	1	0.354
Arrhythmia	1	1.6	1	0	0	0	1	1	1	>0.999
**Gastrointestinal disorders**	**4**	**6.5**	**5**	**0**	**0**	**0**	**4**	**4.2**	**5**	**0.294**
Nausea	2	3.2	2	0	0	0	2	2.1	2	0.538
Abdominal pain	1	1.6	1	0	0	0	1	1	1	>0.999
Constipation	1	1.6	1	0	0	0	1	1	1	>0.999
Diarrhoea	1	1.6	1	0	0	0	1	1	1	>0.999
**General disorders and administration site conditions**	**2**	**3.2**	**2**	**1**	**2.9**	**1**	**3**	**3.1**	**3**	**>0.999**
Fatigue	1	1.6	1	0	0	0	1	1	1	>0.999
Peripheral swelling	0	0	0	1	2.9	1	1	1	1	0.354
Pyrexia	1	1.6	1	0	0	0	1	1	1	>0.999
**Infections and infestations**	**1**	**1.6**	**1**	**1**	**2.9**	**2**	**2**	**2.1**	**3**	**>0.999**
Urinary tract infection	1	1.6	1	1	2.9	1	2	2.1	2	>0.999
Bacteraemia	0	0	0	1	2.9	1	1	1	1	0.354
**Injury, poisoning and procedural complications**	**2**	**3.2**	**2**	**1**	**2.9**	**1**	**3**	**3.1**	**3**	**>0.999**
Injection site pain	1	1.6	1	0	0	0	1	1	1	>0.999
Injection site reaction	1	1.6	1	0	0	0	1	1	1	>0.999
Tooth avulsion	0	0	0	1	2.9	1	1	1	1	0.354
**Vascular disorders**	**3**	**4.8**	**3**	**0**	**0**	**0**	**3**	**3.1**	**3**	**0.55**
Hot flush	3	4.8	3	0	0	0	3	3.1	3	0.55
**Nervous system disorders**	**1**	**1.6**	**1**	**1**	**2.9**	**1**	**2**	**2.1**	**2**	**>0.999**
Seizure	1	1.6	1	0	0	0	1	1	1	>0.999
Syncope	0	0	0	1	2.9	1	1	1	1	0.354
**Reproductive system and breast disorders**	**1**	**1.6**	**1**	**1**	**2.9**	**1**	**2**	**2.1**	**2**	**>0.999**
Erectile dysfunction	1	1.6	1	0	0	0	1	1	1	>0.999
Gynaecomastia	0	0	0	1	2.9	1	1	1	1	0.354
**Skin and subcutaneous tissue disorders**	**1**	**1.6**	**2**	**0**	**0**	**0**	**1**	**1**	**2**	**>0.999**
Rash	1	1.6	2	0	0	0	1	1	2	>0.999
**Metabolism and nutrition disorders**	**0**	**0**	**0**	**1**	**2.9**	**1**	**1**	**1**	**1**	**0.354**

© 2022 Nickols NG et al. *JAMA Network Open.*

Hyperglycaemia	0	0	0	1	2.9	1	1	1	1	0.354
**Musculoskeletal and connective tissue disorders**	**0**	**0**	**0**	**1**	**2.9**	**1**	**1**	**1**	**1**	**0.354**
Pain in extremity	0	0	0	1	2.9	1	1	1	1	0.354
**Respiratory, thoracic and mediastinal disorders**	**0**	**0**	**0**	**1**	**2.9**	**1**	**1**	**1**	**1**	**0.354**
Dyspnoea	0	0	0	1	2.9	1	1	1	1	0.354

© 2022 Nickols NG et al. *JAMA Network Open.*

eTable 5. Serious Adverse Events for Randomized Participants by Treatment Groups

Body System and Preferred Term	Degarelix (N= 62)			Placebo (N= 34)			Total (N= 96)			P-value
	N	%	Events	N	%	Events	N	%	Events	
**All Serious Adverse Events**	**19**	**30.6**	**30**	**11**	**32.4**	**13**	**30**	**31.3**	**43**	**0.863**
**Infections and infestations**	**11**	**17.7**	**13**	**5**	**14.7**	**5**	**16**	**16.7**	**18**	**0.703**
COVID-19 pneumonia	5	8.1	5	4	11.8	4	9	9.4	9	0.716
Septic shock	4	6.5	4	0	0	0	4	4.2	4	0.294
COVID-19	3	4.8	3	0	0	0	3	3.1	3	0.55
Gastroenteritis	0	0	0	1	2.9	1	1	1	1	0.354
SARS-CoV-2 sepsis	1	1.6	1	0	0	0	1	1	1	>0.999
**Respiratory, thoracic and mediastinal disorders**	**6**	**9.7**	**8**	**5**	**14.7**	**5**	**11**	**11.5**	**13**	**0.512**
Respiratory failure	3	4.8	3	4	11.8	4	7	7.3	7	0.24
Dyspnoea	1	1.6	1	1	2.9	1	2	2.1	2	>0.999
Acute respiratory distress syndrome	1	1.6	1	0	0	0	1	1	1	>0.999
Pleuritic pain	1	1.6	1	0	0	0	1	1	1	>0.999
Pneumomediastinum	1	1.6	1	0	0	0	1	1	1	>0.999
Pulmonary embolism	1	1.6	1	0	0	0	1	1	1	>0.999
**Renal and urinary disorders**	**4**	**6.5**	**4**	**1**	**2.9**	**1**	**5**	**5.2**	**5**	**0.653**
Acute kidney injury	1	1.6	1	1	2.9	1	2	2.1	2	>0.999
Renal failure	2	3.2	2	0	0	0	2	2.1	2	0.538
Chronic kidney disease	1	1.6	1	0	0	0	1	1	1	>0.999
**Blood and lymphatic system disorders**	**2**	**3.2**	**2**	**0**	**0**	**0**	**2**	**2.1**	**2**	**0.538**
Iron deficiency anaemia	1	1.6	1	0	0	0	1	1	1	>0.999
Leukocytosis	1	1.6	1	0	0	0	1	1	1	>0.999
**Vascular disorders**	**0**	**0**	**0**	**2**	**5.9**	**2**	**2**	**2.1**	**2**	**0.123**
Mouth haemorrhage	0	0	0	1	2.9	1	1	1	1	0.354
Orthostatic hypotension	0	0	0	1	2.9	1	1	1	1	0.354
**Cardiac disorders**	**1**	**1.6**	**1**	**0**	**0**	**0**	**1**	**1**	**1**	**>0.999**
Supraventricular tachycardia	1	1.6	1	0	0	0	1	1	1	>0.999
**Gastrointestinal disorders**	**1**	**1.6**	**1**	**0**	**0**	**0**	**1**	**1**	**1**	**>0.999**
Diarrhoea	1	1.6	1	0	0	0	1	1	1	>0.999
**Metabolism and nutrition disorders**	**1**	**1.6**	**1**	**0**	**0**	**0**	**1**	**1**	**1**	**>0.999**
Hyperglycaemia	1	1.6	1	0	0	0	1	1	1	>0.999

© 2022 Nickols NG et al. *JAMA Network Open.*
