# Supplementary material for: Effect of Androgen Suppression on Clinical Outcomes in Hospitalized Men With COVID-19: The HITCH Randomized Clinical Trial
Source: JAMA Netw Open. 2022 Apr 19;5(4):e227852. doi: 10.1001/jamanetworkopen.2022.7852 (PMC9020208; doi:10.1001/jamanetworkopen.2022.7852)
Supplement: Supplement 3. — Data Sharing Statement [file jamanetwopen-e227852-s003.pdf]

Data Sharing Statement

Nickols. Effect of Androgen Suppression on Clinical Outcomes in Hospitalized Men With COVID-19. *JAMA Netw Open*. Published April 19, 2022.
doi:10.1001/jamanetworkopen.2022.7852

Data
Data available: Yes
Data types: Deidentified participant data
How to access data: Per Department of Veterans Affairs Office of Research & Development Data Management and Access Plan (DMAP), Final data sets that are de-identified resulting from this research will be shared upon written request. Individually identifiable private information, protected health information (PHI), and other VA sensitive data will not be shared.
When available: With publication

Supporting Documents
Document types: None

Additional Information
Who can access the data: Per Department of Veterans Affairs Office of Research & Development Data Management and Access Plan (DMAP), Final data sets that are de-identified resulting from this research will be shared upon written request. Individually identifiable private information, protected health information (PHI), and other VA sensitive data will not be shared.
Types of analyses: Per Department of Veterans Affairs Office of Research & Development Data Management and Access Plan (DMAP), Final data sets that are de-identified resulting from this research will be shared upon written request. Individually identifiable private information, protected health information (PHI), and other VA sensitive data will not be shared.
Mechanisms of data availability: Per Department of Veterans Affairs Office of Research & Development Data Management and Access Plan (DMAP), Final data sets that are de-identified resulting from this research will be shared upon written request. Individually identifiable private information, protected health information (PHI), and other VA sensitive data will not be shared.
Any additional restrictions: Per Department of Veterans Affairs Office of Research & Development Data Management and Access Plan (DMAP), Final data sets that are de-identified resulting from this research will be shared upon written request. Individually identifiable private information, protected health information (PHI), and other VA sensitive data will not be shared.
